# Australian voters’ attitudes to climate action and their social-political determinants

**DOI:** 10.1371/journal.pone.0248268

**Published:** 2021-03-24

**Authors:** R. M. Colvin, Frank Jotzo

**Affiliations:** Crawford School of Public Policy, Australian National University, Acton, Australian Capital Territory, Australia; Shenzhen University, CHINA

## Abstract

Australia is a relative laggard on climate policy, amidst social and political fractures despite rising support for climate policy in opinion polls. In the 2019 Australian federal election, which was dubbed the ‘climate election’, the opposition campaigned on comparatively ambitious climate action but the government was returned on a status quo policy. We explore the social-political determinants of climate attitudes and how they are positioned in relation to voting behaviour, in the context of the 2019 election. We use a large nationally representative survey of Australian voters (n = 2,033), and employ univariate and multivariate ordinal logistic regression models to uncover correlates. We find that a large majority of voters think it is important for Australia to reduce greenhouse gas emissions. However, the importance given to emissions reductions is sharply divided along lines of political party preference. Holding pro-climate action attitudes consistently correlates with voting for progressive political parties and having higher levels of education. We also find a strong age cohort divide, with younger people holding stronger pro-climate attitudes than older people, raising the question whether we are seeing the emergence of a new generation expressing strong pro-climate action and progressive political attitudes that will persist over time. We conduct population ageing scenarios to project changes to public opinion, by age group, into the future. These indicate that strong support for climate action would increase by about four percentage points over the coming decade as younger voters replace the old, if attitudes within cohorts remained fixed. We conclude that while cleavages in climate attitudes in Australia are set to continue, efforts to promote climate delay are bound to have a limited shelf life as a growing majority of voters accepts the need for climate action.

## Introduction

Australia, politically and socially, has been failing to achieve, or even seek, a consensus approach to climate policy for over a decade. Climate change has been a prominent and contentious political issue, was central to several election campaigns, and implicated in the overturn of multiple Prime Ministers [1–3]. These political fractures are reflected in Australian public opinion, with social divides on climate change following demographic and political cleavages [4]. Research from the mid-2010s positions Australia as second only to the United States in terms of how divided along left-right political lines are attitudes toward climate change [5]. The social-political divide has been fuelled by personalities prominent in politics and the media [6] and well-funded campaigns by the fossil fuel industry [7, 8].

In this politically polarised context, Australia remains a laggard on climate policy [9, 10] while being one of the largest per capita greenhouse gas emitters [11]. Australia was the first country to repeal a price on emissions, in 2014, after implementation in a turbulent political context two years prior [1, 12]. However, public opinion polling data has shifted, dramatically, in recent years suggesting a step change in Australian public attitudes toward climate change [13–17]. According to 14 years of public polling data conducted by a prominent Australian think tank [16], as of 2020 the proportion of the Australian population that considers climate change to be a “serious and pressing problem” requiring immediate action is 56%, whereas this figure was at its lowest, 36%, just eight years prior in 2012. In the same time period, sub-national action, for example targets set by states, territories, and local governments, has well exceeded national-level commitments [18] and substantial changes in the private sector have led to a meaningful engagement with climate risk [19, 20]. These developments indicate that climate change and the need for climate action in Australia is becoming increasingly accepted for many voters, sub-national governments and businesses.

In this context of Australian public opinion, private sector engagement, and sub-national commitments indicating a social setting in which ambitious climate policy would be supported, Australia’s 2019 federal election was viewed by many as an opening climate policy window. Accordingly, the May 2019 federal election once again featured “a fierce battle over climate change policy” [21, writing for Nature News]. The climate policies of the two major parties were night and day, with the Labor Party (centre-left; progressive) campaigning on ambitious mitigation targets and the incumbent Liberal-National Coalition (centre-right/right; conservative) maintaining the status quo of very limited climate policy [22]. Other policy positions, for example on taxation, also differed strongly [23]. Pre-election polling suggested a strong likelihood of a change of government [24], and a prominent online bookmaker went so far as to make early pay outs on bets for a change of government [25].

There was widespread perception of Australia’s 2019 federal election being the decisive ‘climate election’ that would see federal policy overhauled to align with prevailing Australian public opinion [e.g. 26–29]. The ‘climate election’ narrative was particularly pronounced among pro-climate political actors and policy elites. Despite these expectations, the Coalition retained Government with a two-party preferred vote of 51.5%, and a positive swing of 1.2% from the 2016 election result [30]. The returning Coalition Government affirmed no increase in the ambition of Australian climate policy. Post-election survey data suggest that voters in aggregate preferred Labor’s policies on education, health and environment while they preferred the Coalition’s policies on the economy, taxation and immigration, and that climate change and the environment were more important issues for voters than in previous elections [31].

In this paper, we explore the social-political determinants of Australian voters’ attitudes toward climate change in the context of the 2019 federal Australian election via analysis of a large (n = 2,033) probability sample of survey data. We unpack the most significant correlates of measures of aggregate public opinion toward climate change and explore via population ageing scenarios how patterns of Australian public opinion may shift in the decades ahead. From this, we consider the implications for Australian climate policy and politics.

### The Australian electoral system

Australia’s federal election system is based on the Westminster system, where two major parties–the Coalition of the Liberal Party of Australia and The Nationals, and the Australian Labor Party–compete for government. Each of the 151 seats in the lower House of Parliament corresponds to an electorate which is a defined geographical area. The winner of the electorate is whichever candidate wins 50% plus one vote in a preferential voting system. Minor parties and independents hold some seats and otherwise influence the flow of preferences to the major parties. Marginal or swing seats matter more for determining the outcome of an election than do safely held seats. Importantly, patterns in aggregate public opinion do not necessarily translate to election outcomes if these patterns are unevenly distributed across the electorates. The second federal house of government, the Senate, uses state based proportional voting, however, unlike the House of Representatives, a majority in the Senate does not in itself lead to forming government.

As a consequence of these factors, election campaigning routinely focuses on marginal seats, sometimes requiring a balance between messaging on local and national issues. The Coalition victory in the 2019 federal election benefitted from a strong result in the state of Queensland, where the Coalition won 23 out of 30 seats (up from 21 in the 2016 election) [32, 33]. The Coalition’s campaigning in Queensland included narratives about economic and employment costs of climate action [23, 24, 34, 35]. However, economic policy was a dominant point of comparison between the two parties’ policy platforms nationally and in Queensland, as were dynamics of party leadership dynamics and personalities.

An exploration of the social-political determinants of Australians’ attitudes toward climate change and how these attitudes are positioned in relation to voting behaviour yields potentially important insights. For Australia, there are questions about whether climate change is an issue of significance to a large enough proportion of the voting population that it may come to decide future election outcomes based on demand for ambitious action, rather than fear of the social and economic impacts of climate policy [e.g. 2]. Australia’s experience may offer insights for other nations, particularly Anglophone countries with similar culture and systems of governance.

## Materials and methods

We analysed quantitative survey data canvassing climate opinion, political voting behaviour, and a range of demographic measures. These data were collected via inclusion of specific questions in the *Life In Australia* online panel [36] in July 2019 along with a range of questions administered for the Australian Election Study (AES) [31]. The online panel includes a large set of information on respondents’ personal, social and economic characteristics. Our exploration of climate opinion in the context of the 2019 federal election was informed by a combination of the significance of the election outcome for climate policy in Australia, and the vast body of evidence that implicates political affiliation and ideology as a significant driver of climate opinion in Australia [4, 5, 37–40].

To identify the determinants of climate opinion and voting preferences, we analysed our data using ordinal logistic regression (OLR) models, a suitable analytical approach for ordinal survey data [41]. Following this, we created simplified population ageing scenarios to project an approximation of how aggregate public opinion may shift in the future.

### Survey instrument

The survey contained three sections: climate opinion, voting preferences at the May 2019 federal election, and a number of standard demographic variables. Questions and response options are available in Supporting information A.

On climate opinion, we asked participants four questions interrogating various dimensions of climate opinion and voting preferences:

1. In your view, how important or unimportant is it that Australia takes action to reduce greenhouse gas emissions in order to help limit future climate change? (Measures of importance)
2. In your view, which energy source or mix of energy sources should provide Australia’s electricity in 2050? (Various fuel sources and combinations of sources)
3. To what extent are you prepared to accept a personal cost in order to support action to reduce Australia’s emissions? (Personal costs and implications for others)
4. How much did the issue of climate change influence your vote in the 2019 Federal election? For you personally, would you say climate change was…? (Measures of importance to vote)

Participants responded to each climate opinion question on an ordinal scale (except for Q2 which was categorical). For each question, we were interested in both describing broad trends and interrogating the determinants of variation among the respondents. Question 1 was intended to verify trends from public polling in aggregate Australian public opinion on climate change. In this way, we did not examine trends in specific or swing electorates [e.g. 42] but instead focused on aggregate public opinion in recognition of the policy relevance of such a measure [43, 44]. Question 2 sought to describe preferences for energy sources, given the centrality of the energy sector to climate policy debates in Australia [34, 45]. In question 3 we considered the willingness for individuals to make a sacrifice in order to support climate policy initiatives. This is in recognition of the pernicious attitude-behaviour gap that has seen limited climate-related behaviour change even in settings where opinion is broadly aligned with such goals [38, 46, 47]. Question 4 placed climate opinion in the voting context, to examine to what extent Australian voters factored climate change into their decisions for allocating their primary vote in the House of Representatives in the May 2019 federal election. Question 4, then, was intended to examine whether the 2019 election (held around two months before the survey) was indeed the ‘climate election’ in the minds of Australian voters.

Voting preference at the May 2019 federal election was measured categorically in line with the Australian Election Study approach. Participants were asked “In the Federal election for the House of Representatives on Saturday 18 May, which party did you vote for first in the House of Representatives?”, and provided with a list of all political parties. For the purposes of our analysis, we aggregated into the major parties (plus independent/other): Liberal Party (centre-right/right; conservative), Labor Party (centre-left; progressive), The Greens (left; progressive), The Nationals (centre-right/regional issues; conservative), The Liberal-National Party (centre-right/right; conservative; present in Queensland only as a formal merging of the two Coalition partner parties into the LNP). While the Liberal and National Parties co-govern as a Coalition, due to differences between the results corresponding to the two parties we maintained the distinction in the analysis. The formal merger of the two parties in Queensland complicates the analysis as we did not want to aggregate LNP voters into either Liberal or National Party voters. Therefore, we also analysed the LNP separately alongside the Liberal and National Parties.

We accessed the standard demographic variables collected for the Australian Election Study. These were: state of residence (categorical), residence in capital city or non-capital city (binary categorical), socio-economic index for area of residence (ordinal), gender (categorical), age (numerical), country of birth (categorical), citizenship (binary categorical), language (binary categorical), income (ordinal).

### Sample

The sample was accessed via the *Life In Australia* online panel [36], a national probability-based online panel comprising of Australian residents aged 18 and older. The survey was administered via telephone and online between 8 and 22 July 2019; 2,620 panel members were invited and 2,033 of these completed the survey. The sample broadly reflects the Australian population across key demographic variables, though the age profile of our sample is older than the Australian population. Of the sample, 52.7% were female and the median age was 55; the sample is therefore somewhat skewed towards older respondents as the median age of Australian adults (18 and over) is 45 [48]. Full demographic descriptive data on the sample is available in Supporting information C.

### Data analysis

Analyses of the ordinal climate opinion questions (Qs 1, 3, 4) were conducted using ordinal logistic regression (OLR) models. OLR analysis is appropriate for examining ordinal dependent variables with one or more independent variables [41], e.g. as used extensively by Tranter [4, 35, 40, 49–53]. We conducted univariate OLR analyses, then multivariate OLR analyses including the variables that were significant in univariate OLR analyses [following 54].

Analysis of the categorical question (Q2), regarding preferences for Australia’s future energy mix, was conducted using standard parametric (i.e. ANOVA) and nonparametric (i.e. Pearson’s chi-squared test of independence) analyses as appropriate based on data type. By dividing the sample into sub-groups according to their response to Q2, we were able to assess for differences in demographic and voting variables within each of the Q2 response options.

Following analysis of the trends and determinants of climate opinion in the context of the 2019 Australian federal election, we then constructed two basic population ageing scenarios to predict how aggregate opinion trends (related to Q1; importance of Australia reducing GHG emissions) might change in the future in line with the entry of younger voters and exit of older voters, if attitudes within each cohort remained stable. For this analysis, we used Australian Bureau of Statistics (ABS) population projections for 2017–2066 [48] from which we drew age distribution projections at the 5-year age bracket (18–22; 23–27 etc.) level. We then explored what would be the implications for the distribution of the opinion that climate action is ‘extremely important’ (Q1) based on a growing proportion of the voter base holding opinion in line with or stronger than current young people.

An extended description of all analytical methods is available in Supporting information B.

## Results

Here we present a summary of our key findings. Full results, including all statistical details, are available in Supporting information C.

### On the importance of Australia reducing greenhouse gas emissions

**Fig 1:**
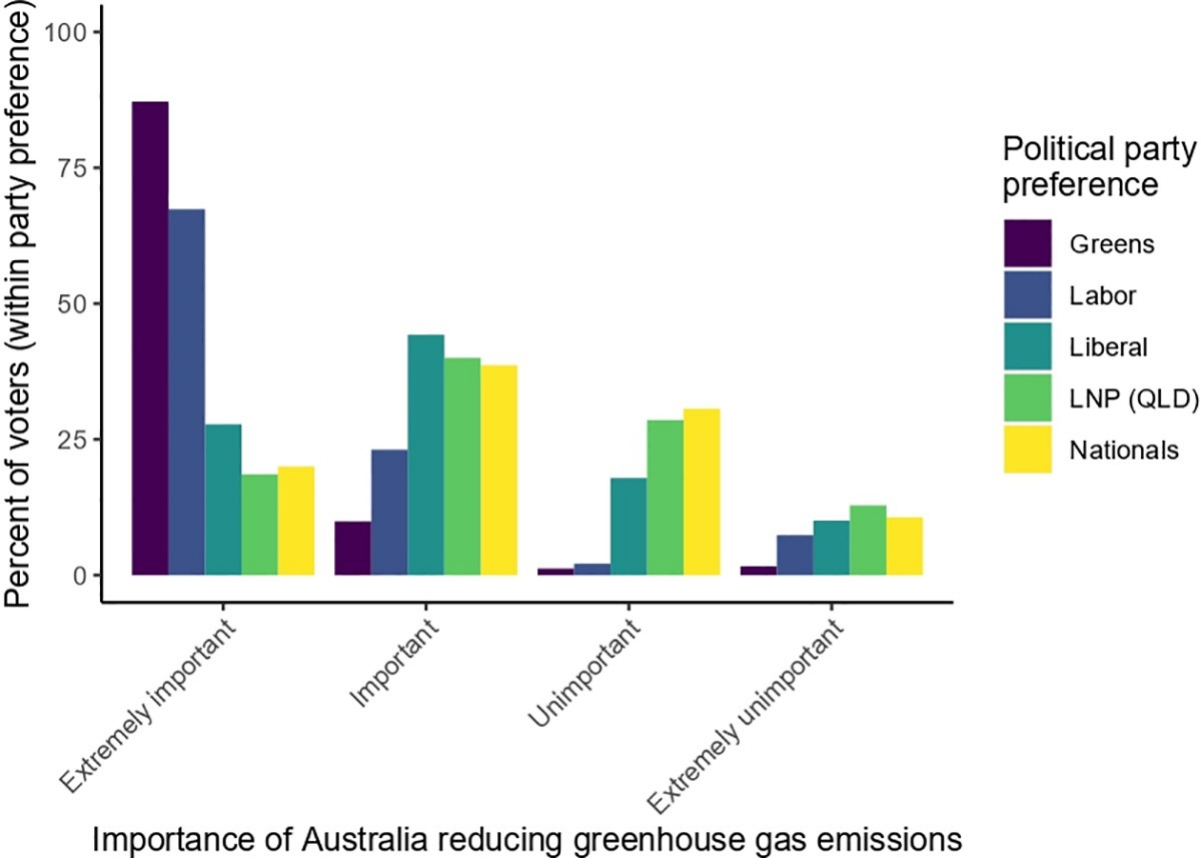
Distribution (by percent of all voters for each of the major political parties) of opinion about the importance of Australia reducing greenhouse gas emissions.

Our analysis showed that over 80% of Australians in the survey profess that they view action to reduce Australia’s emissions as important, including almost 70% of conservative (Liberal, National, Liberal-National Coalition) voters. But they are sharply divided along party lines about the importance of the issue. Three quarters (73%) of progressive voters see Australian action to reduce emissions as extremely important, while only one quarter (26%) of conservative voters say it is extremely important (Fig 1).

**Fig 2:**
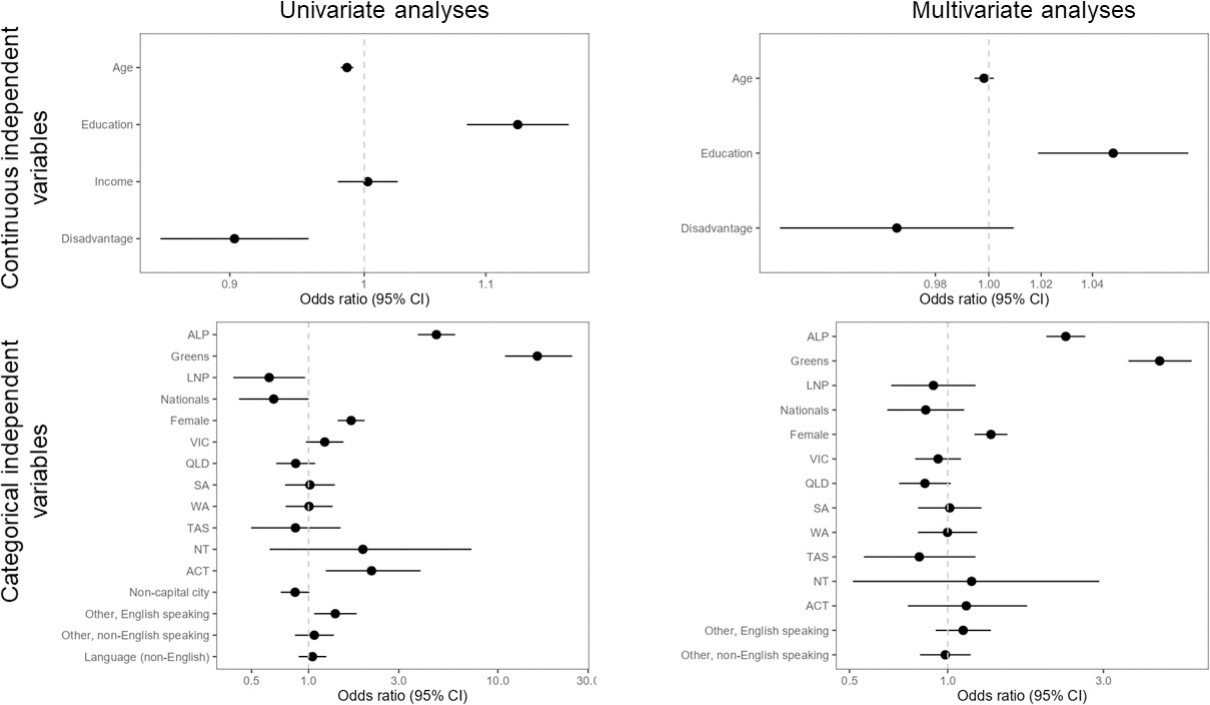
Forest plots illustrating odds ratios and 95% confidence interval of the odds ratio for Q1: “In your view, how important or unimportant is it that Australia takes action to reduce greenhouse gas emissions in order to help limit future climate change?” Left panels are univariate analyses; right panels are multivariate analyses; upper panels are continuous independent variables; lower panels are categorical independent variables. For continuous independent variables, the variables have been modified where necessary so that the OR is measuring more quantity of the variable listed on the y axis (i.e., more income, more education, more disadvantage in the area of residence, more age). For categorical variables, comparisons are to the default baseline category only (baseline category not displayed in plots: Liberal; Male; NSW; Capital city; English speaking background; Language (English)). Variables that are statistically significant at (α = 0.05) do not cross the OR value of 1, indicated by dashed vertical line, with their 95% confidence interval. Note the differing scales on the x-axes across plots.

Using multivariate ordinal logistic regression (proportional odds), we identify characteristics that are statistically significant and meaningful in explaining the variation in stated support for Australia reducing greenhouse gases (Fig 2). These variables were (factors predicting higher support in parentheses): political party preference (Greens & Labor p < 0.0001), educational attainment (more education p = 0.0012), and gender (women p < 0.0001).

Age is also highly significant (younger p < 0.0001 in univariate analysis), as is the prevalence of socioeconomic disadvantage in the local area where respondents live (less disadvantage p = 0.0006 in univariate analysis), but both become non-significant in multivariate analysis, i.e. when analysed in the context of the other independent variables.

If we view age in isolation from other variables such as political party preference, based on PEW generational categories [55] “Baby Boomers” (born 1946–1964) are half as likely as “Gen Z” (born 1996–2012) to consider it important to reduce greenhouse gas emissions (OR of generational gap = 0.52, p < 0.0001). However, there is high covariance between age and political party preference, and in our analysis which treats all independent variables as equal (i.e. it does not recognise causal or path relationships between independent variables) political party preference is better at explaining differences in attitudes toward the importance of Australia reducing GHG emissions. The average age is older among those people who vote for Australia’s major conservative parties, and younger for those who vote for the progressive parties (ANOVA of age: p < 0.001; Liberal voter = 58.1^a^ (mean age), ALP voter = 53^b,c^, Nationals voter = 62.8^a^, Greens voter = 46.5^d^, LNP voter = 58.2^a,c^; superscript letters indicate similarity/difference using Tukey’s multiple comparisons of means at α = 95%).

Which State respondents live in, and whether they live in capital cities or outside, is not a statistically significant determinant of stated support for climate change action, though these variables became significant in other climate opinion questions.

### On how important climate change was to one’s vote: The ‘climate election’ that wasn’t

**Fig 3:**
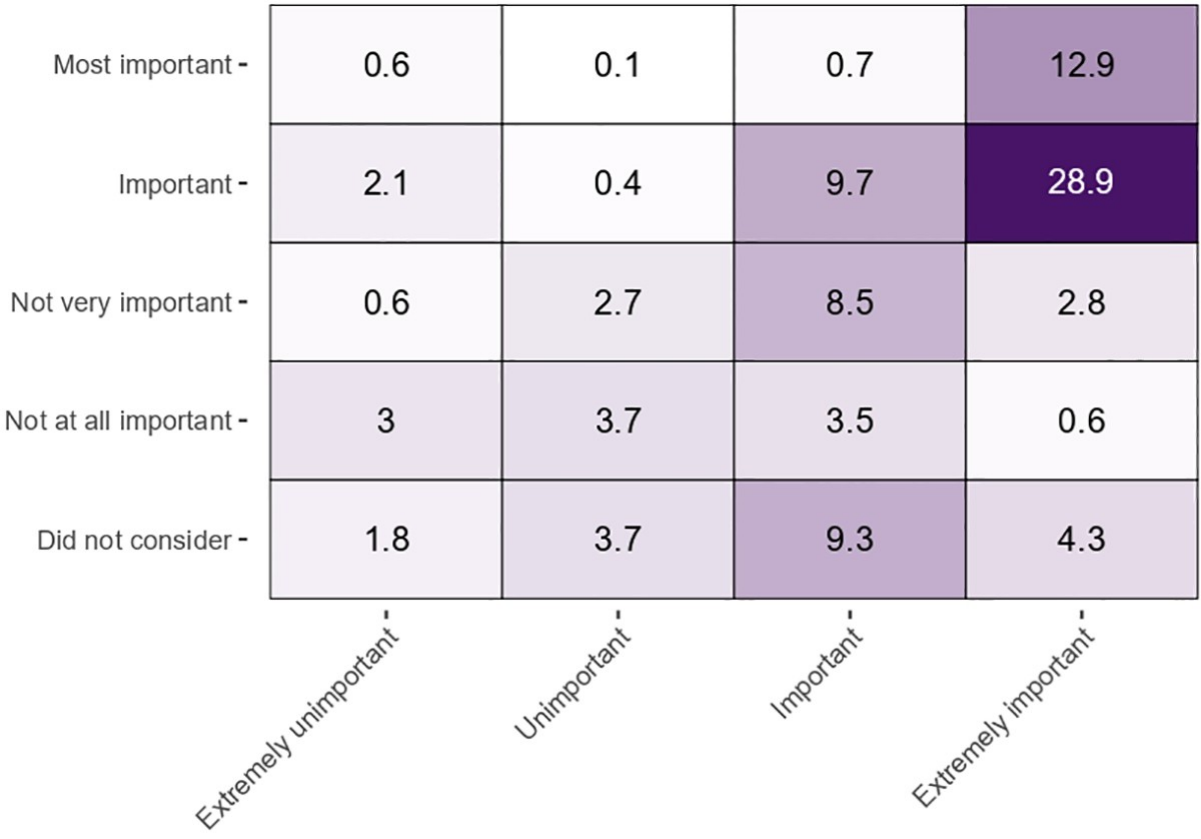
Cross-tabulation of responses to the questions about importance of action and importance to vote, showing percent of all responses. “In your view, how important or unimportant is it that Australia takes action to reduce greenhouse gas emissions in order to help limit future climate change?” (x axis) and “How much did the issue of climate change influence your vote in the 2019 Federal election? For you personally, would you say climate change was…?” (y axis).

About half of the respondents (52%) reported that climate change was important when deciding their vote in the 2019 Australian federal election. However, it was the most important issue for only 13%. Among the respondents who considered greenhouse gas reductions to be ‘extremely important’, the majority indicated that climate change was an ‘important’, but not the ‘most important’, issue when deciding their vote at the 2019 federal election (Fig 3). This suggests that climate change was an important factor, but not the decisive factor for a large majority of voters.

**Fig 4:**
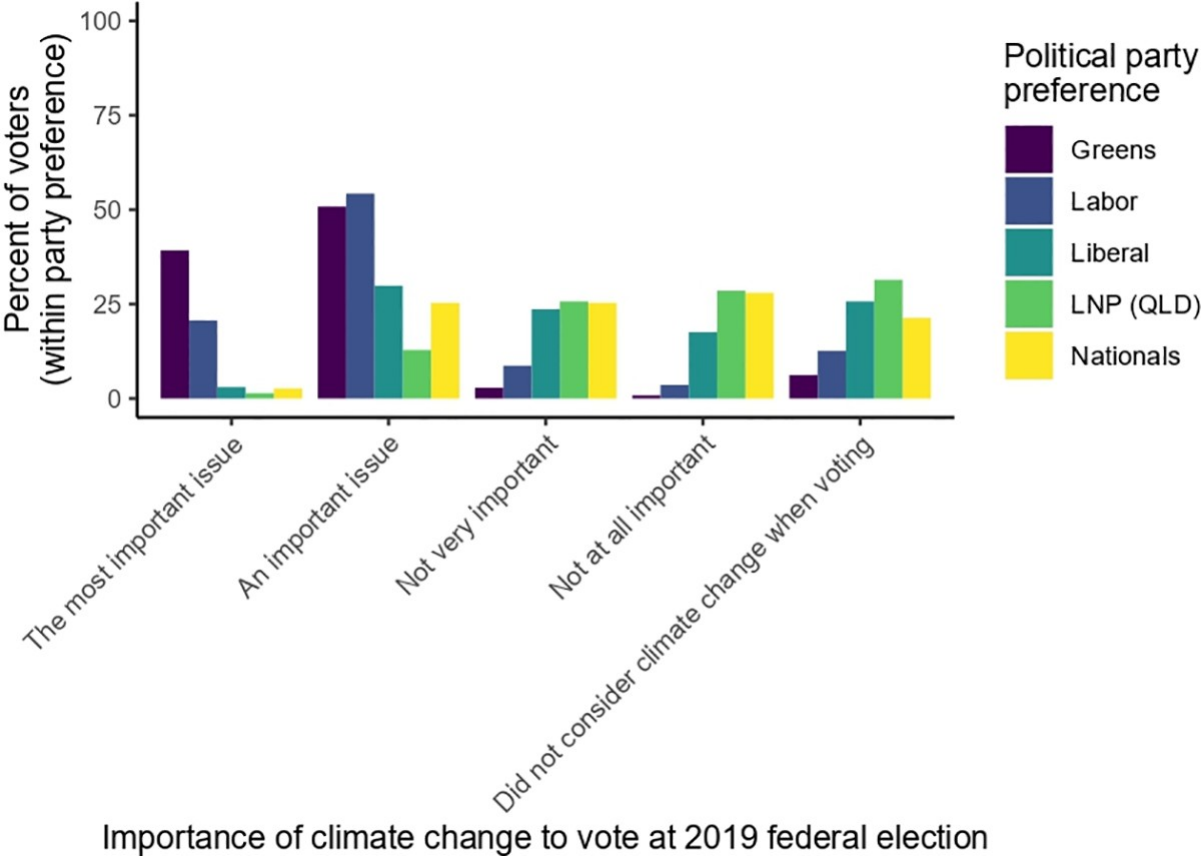
Distribution (by percent of all voters for each of the major political parties) of opinion about the importance of climate change when determining their vote in the House of Representatives at the 2019 federal election.

Climate change was a more important issue for progressive voters (79% stated "most important" or "important") than for conservative voters (30% stated “most important” or “important”) (Fig 4).

Climate change was the most important issue for 21% of Labor voters and 39% of Greens voters, but the most important issue for only 3% of Liberal Party voters, 2.6% of National Party voters, and 1.4% of LNP voters (Table 1). These patterns were broadly mirrored among those who did not consider climate change: 12.6% of Labor and 6.2% of Greens voters, compared to 25.6% of Liberal, 20.8% of National, and 31% of Liberal-National Party voters.

**Table 1:** Percent of voters of each of the main parties indicating the importance of climate change to their vote at the 2019 federal election (Q4). Percentages are within party (within columns).

Importance of climate change to vote	Political party preference				
	Liberal Party	Labor Party	Greens	National Party	Liberal-National Party
Most important	3.0	20.7	39.3	2.6	1.4
Important	29.7	54.3	50.8	24.7	12.7
Not very important	23.5	8.7	2.9	24.7	25.4
Not at all important	17.5	3.6	0.8	27.3	28.2
Did not consider	25.6	12.6	6.2	20.8	31.0

**Fig 5:**
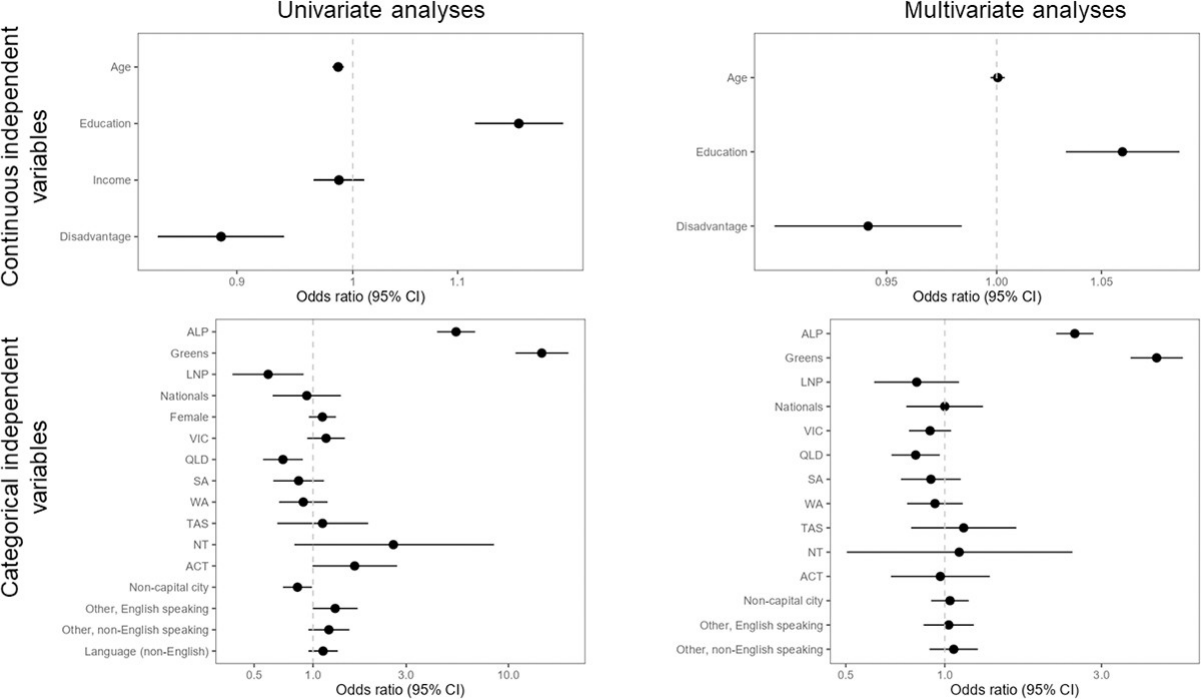
Forest plots illustrating odds ratios and 95% confidence interval of the odds ratio for Q4: “How much did the issue of climate change influence your vote in the 2019 Federal election? **For you personally, would you say climate change was…?”** Left panels are univariate analyses; right panels are multivariate analyses; upper panels are continuous independent variables; lower panels are categorical independent variables. For continuous independent variables, the variables have been modified where necessary so that the OR is measuring more quantity of the variable listed on the y axis (i.e., more income, more education, more disadvantage in the area of residence, more age). For categorical variables, comparisons are to the default baseline category only (baseline category not displayed in plots: Liberal; Male; NSW; Capital city; English speaking background; Language (English)). Variables that are statistically significant at (α = 0.05) do not cross the OR value of 1, indicated by dashed vertical line, with their 95% confidence interval. Note the differing scales on the x-axes across plots.

Ordinal logistic regression (multivariate) shows as significant political party preference (Greens & Labor p < 0.0001), educational attainment (more education p < 0.0001), and living in an area with lower socio-economic disadvantage (regardless of personal status) (less disadvantage p = 0.0072) (Fig 5). Additionally a difference was found between residents of NSW and QLD, with NSW residents more likely than residents of QLD to consider climate change in their voting decision (p = 0.0184). However, there were no other differences between residents of any other states/territories, including all other states/territories compared to both QLD and NSW.

In univariate analysis, age was significant (p < 0.0001), though it lost significance in multivariate analysis when it was considered in the context of the other independent variables. This can be explained, once again, by the strong relationship between age and political party preference, and the multivariate OLR treating all independent variables as equal (i.e. not recognising causal/path relationships between independent variables).

### On the willingness to incur a personal cost in efforts to address climate change

**Fig 6:**
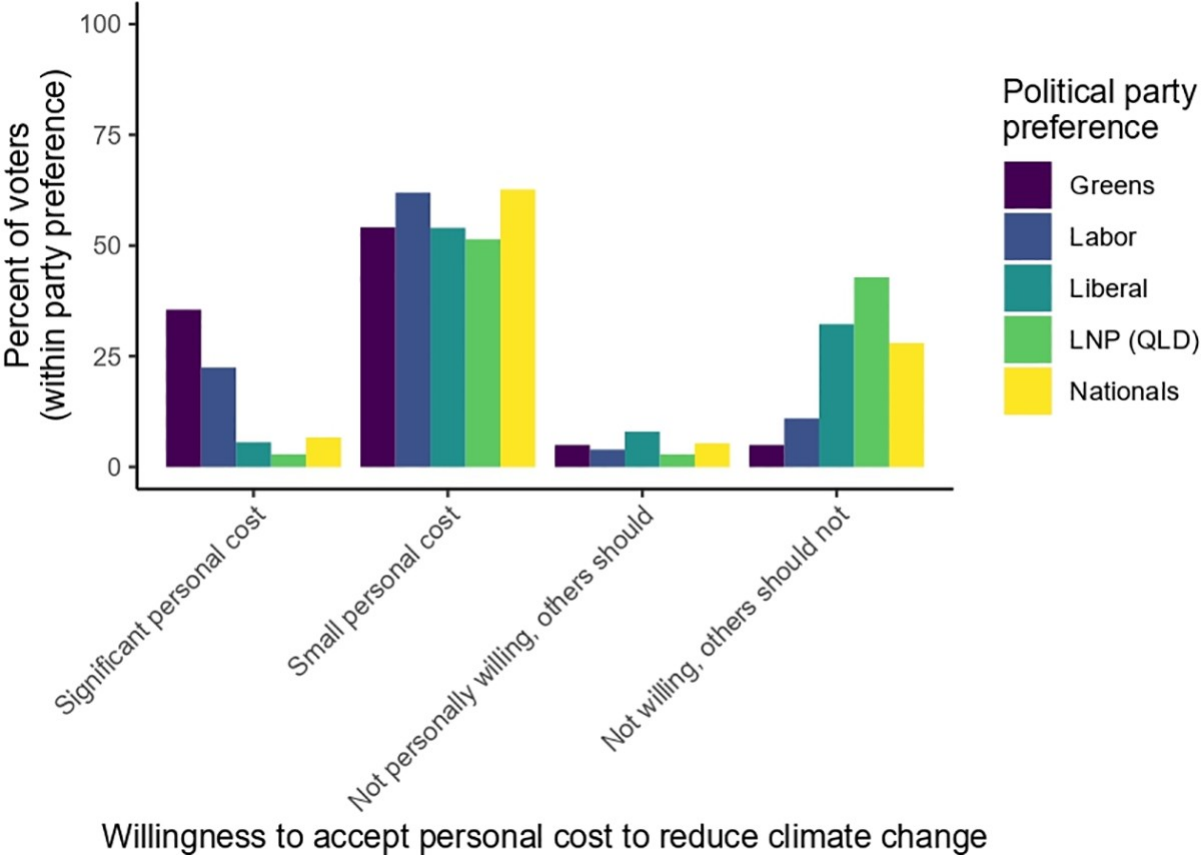
Distribution (by percent of all voters for each of the major political parties) of opinion about willingness to accept a personal cost to reduce climate change.

A majority of respondents (72%) reported that they would be willing to incur some personal cost in return for emissions reductions, but only 15% indicated they would be willing to wear ‘significant’ personal cost. Across political party preference, the proportion of voters who are willing to accept a small personal cost is relatively similar (60% of progressive voters, 55% of conservative voters), but there are differences in distributions in those who are willing to incur a ‘significant’ personal cost, and those who are not willing to incur any personal cost at all (Fig 6). While 26% of progressive voters are willing to incur a significant personal cost, only 5% of conservative voters feel similarly. At the other end of the spectrum, 40% of conservative voters are unwilling to incur any personal cost, but only 13% of progressive voters feel the same.

**Fig 7:**
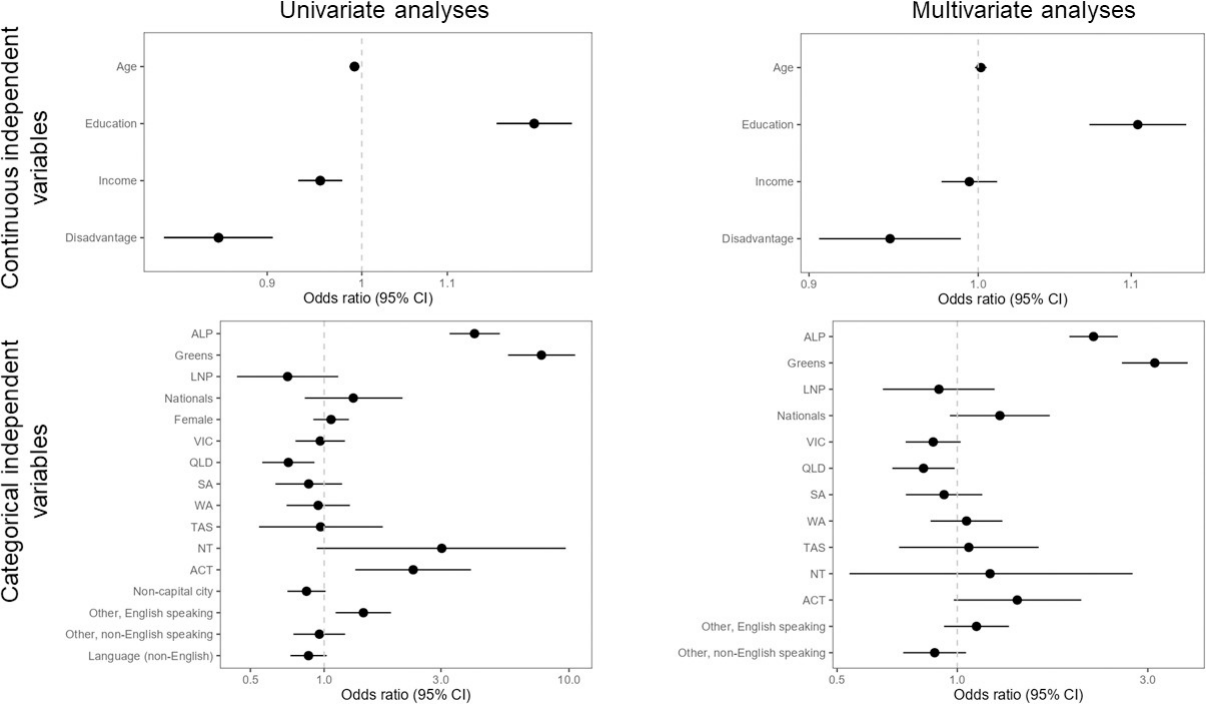
Forest plots illustrating odds ratios and 95% confidence interval of the odds ratio for Q3: “To what extent are you prepared to accept a personal cost in order to support action to reduce Australia’s emissions?”. Left panels are univariate analyses; right panels are multivariate analyses; upper panels are continuous independent variables; lower panels are categorical independent variables. For continuous independent variables, the variables have been modified where necessary so that the OR is measuring more quantity of the variable listed on the y axis (i.e., more income, more education, more disadvantage in the area of residence, more age). For categorical variables, comparisons are to the default baseline category only (baseline category not displayed in plots: Liberal; Male; NSW; Capital city; English speaking background; Language (English)). Variables that are statistically significant at (α = 0.05) do not cross the OR value of 1, indicated by dashed vertical line, with their 95% confidence interval. Note the differing scales on the x-axes across plots.

Ordinal logistic regression (multivariate) analysis shows that the variation in responses to the question of willingness to incur a personal cost is best explained by political party preference (Greens & Labor p < 0.0001), educational attainment (more education p < 0.0001), and living in an area with lower socio-economic disadvantage (regardless of personal status) (less disadvantage p = 0.0148) (Fig 7). We also found some differences between residents of different states/territories. Residents of Queensland were less inclined than residents of the Australian Capital Territory (p = 0.0051), Western Australia (p = 0.0394), and New South Wales (p = 0.0338) to accept a personal cost. Residents of the Australian Capital Territory were more inclined to accept a personal cost than residents of Victoria (p = 0.0085) and South Australia (p = 0.0369), as well as residents of Queensland.

As with the prior questions, age was highly significant in univariate analysis (with younger people more willing than older people to accept a personal cost: OR 1.0017, p < 0.0001) but non-significant in multivariate analysis (again, note that multivariate OLR does not recognise causal/path relationships between the independent variables). While women were more likely than men to state that national GHG reductions are important (Q1), the gendered difference did not emerge in response to the question about taking on a personal cost.

### Views on future energy sources: Renewable energy future broadly accepted

Renewable energy is seen as the future for Australia’s energy system by a large majority; over 85% see renewable energy contributing at minimum half of Australia’s future electricity. Thirty percent think that the electricity grid should be supplied *mostly* by renewables. A sizeable minority (10%) sees nuclear power play an important role in the future. We used parametric (ANOVA) and non-parametric (Pearson’s chi-squared test of independence) analyses to examine for associations between energy preferences and demographic variables. We found a strong signal of influence from political party preference, age, gender, and education. Women (p < 0.001), younger people (p <0.001), people living in cities (p = 0.006), and people with higher educational attainment (p < 0.001) show the strongest positive association with support for renewable energy. Meanwhile, men, (p < 0.001) people from NSW and QLD (p = 0.017), and LNP and Liberal voters (p < 0.001) show the strongest positive association with support for fossil fuels. With regard to nuclear energy, men (p < 0.001), Liberal voters (p < 0.001), and less educated people (p = 0.022 compared to mostly renewables and some fossil fuels & p < 0.001 compared to all renewables) show the strongest positive association with support.

### Demographics: Generational differences are pronounced but change is slow

Age was not found to be a significant variable in the multivariate analyses. But, due to its significance in univariate analyses and the fact that ageing is a guaranteed change over time among the population (compared to, for example, distribution of political party preference) we did further analysis on the potential for age-related opinion trends to change aggregate public opinion over time. In an analysis of the possible effect of demographic change over time, we focus on the share of respondents who indicate they believe it is ‘extremely important’ to reduce greenhouse gas emissions (50% in our sample, or 52% after weighting our survey data by age to reflect the age distribution in the Australian population). We considered both what may happen if today’s young people (18–28 year olds) will be representative of future young people with regard to attitudes about climate change (scenario 1), and what may happen if future young people will hold views even stronger than today’s young people (scenario 2). We assumed there is no change in views of the current adult population, that is, the proportion of people who consider it ‘extremely important’ to reduce GHG emissions will remain constant for the cohorts surveyed as they age (e.g. 65.2% of 18–22 year olds in 2019 will become 65.2% of 23–27 year olds in 2024).

**Fig 8:**
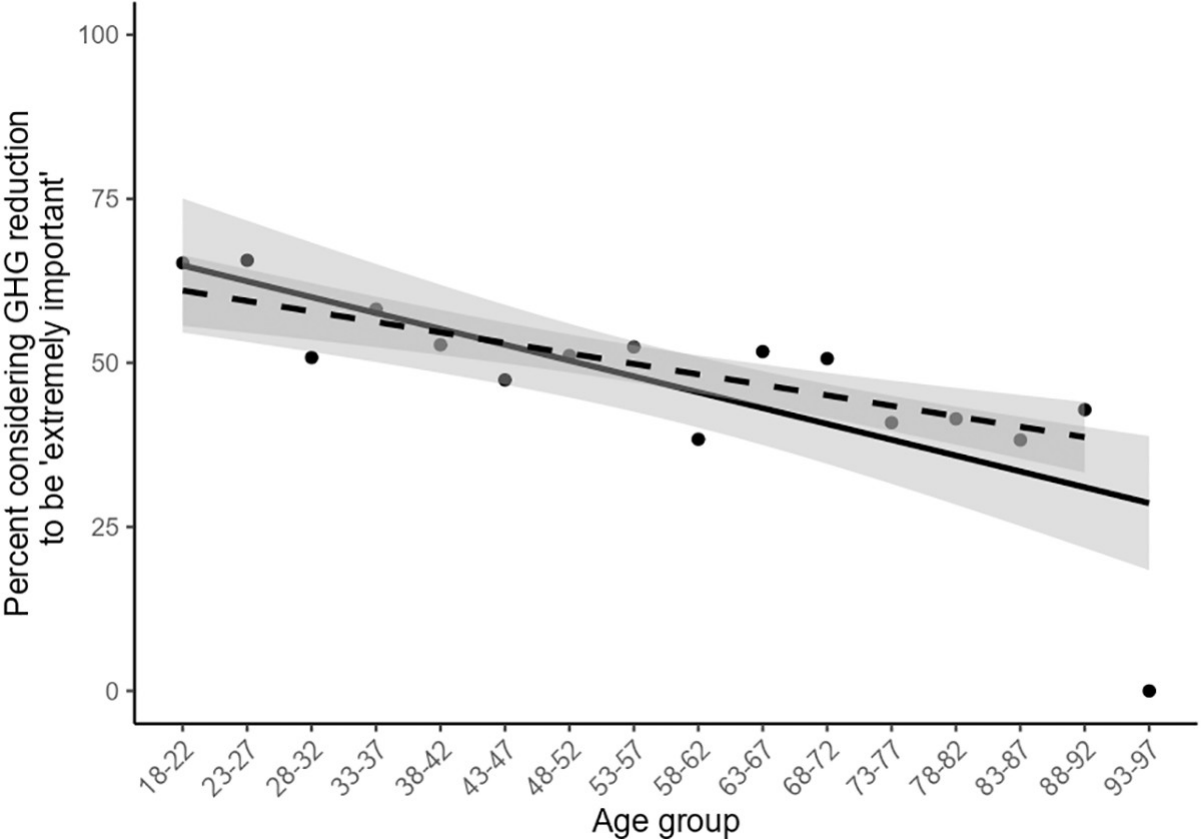
The percent of each age bracket answering ‘extremely important’ to Q1. Solid line indicates linear model including the 93+ age group; dashed line indicates linear model with the 93+ age group excluded. Shading represents standard error of the linear models.

Breaking our sample into 5-year age brackets (18–22; 23–27 etc.) we identified the proportion of each 5-year age group that considers GHG emissions reduction to be extremely important. We then applied these opinion data to the ABS population projections at five yearly intervals starting in 2024 through to 2064. For scenario 1, we assumed all future young people will hold views in line with 2019’s young people. For scenario 2, we assumed future young people will hold views proportionately stronger than 2019’s young people. To apply a reasonable proportion for our projections in scenario 2, we conducted linear regression on the percent of each age bracket answering ‘extremely important’ to Q1, predicted by age (% of age bracket = 73.5293–0.4829x, where x = the youngest age in the age bracket (i.e. 18 for 18–22), p < 0.001, DF = 14, R^2^ = 0.5869, Fig 8). We used the slope coefficient from our linear model to project that the percent of future young people holding opinion aligned with ‘extremely important’ on Q1 would increase by 2.4% for each new group of young people (18–22 year olds) entering the Australian voting population at 5-yearly intervals (0.4829(5) = 2.4145).

A complication for this analysis was that the proportion of respondents in the 93+ age group (9 respondents) who selected ‘extremely important’ was zero. We re-ran the linear regression with the 93+ age group excluded, yielding a slope of 0.3194, which over 5 years equals 1.597 (compared to 2.4145 with the age group included). The results outlined below note differences between the results when the 93+ were included versus excluded, when a difference is evident.

**Fig 9:**
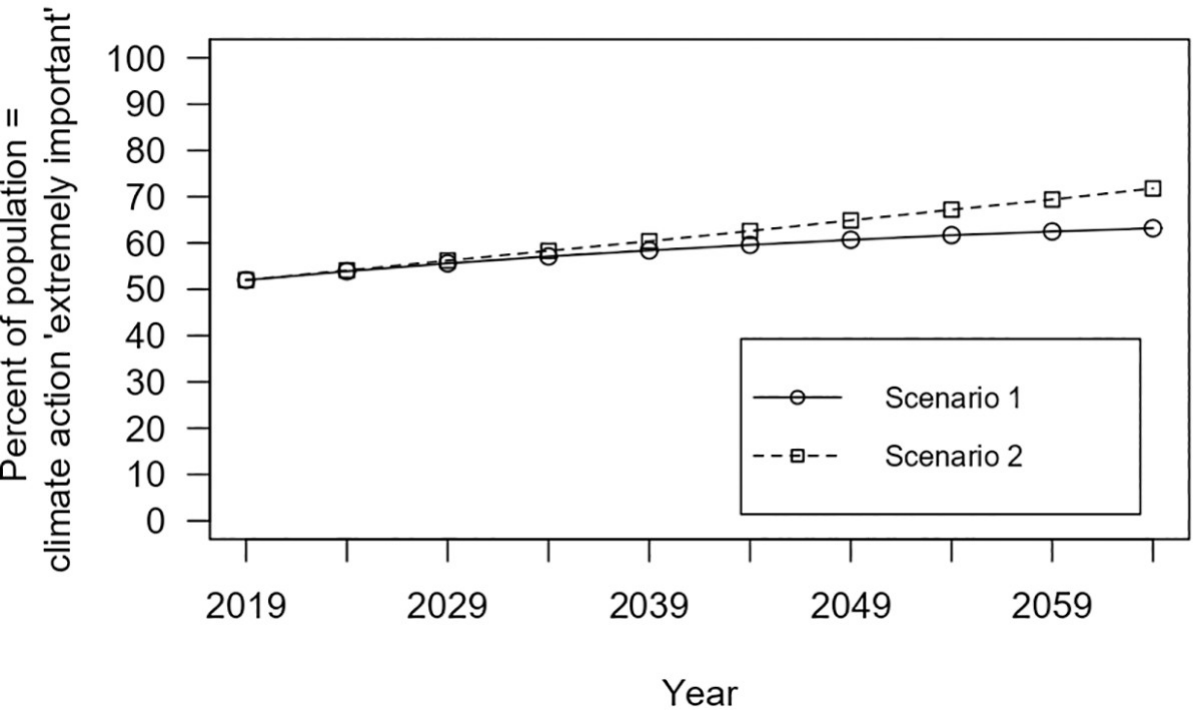
Basic populating ageing scenarios showing future share of Australian public opinion that climate action is ‘extremely important’. These basic scenarios consider influence from entry of younger and exit of older voters, with 2019 levels of public opinion projected onto future age distributions. Scenario 2 displays projections based on calculations including the 93+ age group (scenario 2 projections excluding the 93+ age group would fall between the two lines plotted in the figure).

The results of our basic population ageing scenarios show that within the next decade, we can expect to see an increase from 52% in 2019 to 56% in 2029 of the Australian population who consider GHG emissions reduction ‘extremely important’ (regardless of which scenario is used). By 2049 this figure rises to 61–65% depending on scenario (Fig 9). Thus, the impact of ageing on aggregate attitudes would be around 4% per decade.

Projecting further into the future shows more pronounced change, and greater differences between the scenarios. To the extent of our analysis (following availability of ABS population projection data), in 2064 our analysis indicates we would see 63% of the population considering GHG emissions reduction to be ‘extremely important’ if 2019’s young people are indicative of future young people and cohorts did not change their respective views over time.

If, however, future young people hold views stronger than 2019’s young people, in line with age-related patterns of opinion observed in 2019, then by 2064 our analysis indicates 69–72% of the population may consider GHG emissions reduction ‘extremely important’ (69% based on projections excluding the 93+ age group from the slope, 72% with the 93+ age group included).

## Discussion

Our analysis of climate change opinion data in the context of the May 2019 Australian federal election shows a large majority of voters feel that it is important for Australia to reduce greenhouse gas emissions. However, *how* important is action on climate change is consistently affected by political party preference and educational attainment. We found that progressive voters (Greens and Labor) were consistently more likely than conservative voters (Liberal, Nationals, and Liberal-National Party) to 1) think climate action is extremely important, 2) have considered climate change in their voting decision at the 2019 federal election, and 3) to be willing to incur a personal cost to address climate change. In addition, higher levels of educational attainment predicted the same pattern, but to a lesser magnitude.

These findings indicate that the Australian population, in aggregate, will support climate action. This is the case, too, for the expansion of renewable energy in Australia’s energy mix. Although divisions along lines of political party affiliation remain potent [e.g. 4, 5], these divisions are mostly in the *degree of support* for climate action, not categorical terms of support or opposition.

Our findings also suggest that we can expect the proportion of the Australian population which considers climate action to be important or decisive will increase into the future, given that younger voters exhibit stronger support for reducing emissions, reflecting the findings of recent research by Tranter [53]. However, our findings indicate that the change that is set to arise purely from demography is slow. Assuming that cohorts retain their attitudes through time, a population ageing scenario suggests that strong support for climate action would increase by about four percentage points over the coming decade as younger voters replace the old.

Hence, a large and rapid change in attitudes of the population overall would require existing voting-age people to change their mind. This appears to be happening. Comparing our results to an existing Australian longitudinal study of Australian public opinion on climate change [17] suggests that support for climate action over the last six years rose by two percentage points per year, about four times faster than would be expected on the basis of demographic change alone.

The fact that age is highly significant in univariate analysis, but loses significance due to high covariance with political party preference suggests there is an age cohort divide on climate change opinion in Australia that coincides with differing party political attitudes between older and younger voters [53]. This in turn raises the question of whether we are seeing the emergence of a new generation expressing strong pro-climate action and progressive political attitudes *that will persist over time*. Evidence from studies of voting behaviour and social change would support such an interpretation, particularly due to the influence of social-political events during early adulthood in shaping enduring attitudes [56–62]. But, such questions are highly complex and there is evidence to the contrary, for example indicating political conservatism (and perhaps therefore anti-climate action attitudes) increases over the life course [63, 64], that factors such as race and identity can be more significant than age in shaping attitudes toward contentious social-political issues [65], and that political orientations and identity in youth are fluid compared to mid- and old-age [66, 67].

Our sample is somewhat skewed towards older respondents (see SM Table 2 in S1 File). Given the tendency for younger people to indicate higher levels of support for climate change action, this implies that the results overall indicate somewhat lower levels of support for climate action than a ‘true’ average across the Australian population. However, this should not substantially affect the age-related analyses where we analyse attitudes separately for each age cohort, and extrapolate on the basis of cohort sizes across the population. A bias towards older respondents may exist within each sample cohort, however this is likely a minor effect. Our findings broadly reflect that of recent research which interrogated young Australians’ climate attitudes. Tranter’s [53] analysis of young Australians likewise found that young people tend to hold stronger pro-climate attitudes than older people, though political preference remains the most significant factor. Overall, our data suggest that Australia’s 2019 election was not a ‘climate election’, as was frequently portrayed in the lead up to polling day. This raises important questions for climate policy advocates about whether framing elections as akin to referenda on climate action is an effective strategy. Our findings indicate that the vast majority of Australians support climate action but do not vote according to climate action as a priority. Only very small proportions of Coalition voters considered climate change important to their vote. When elections are won by the party without ambitious climate policies it may serve as a symbolic warrant for governing parties to ignore an otherwise compelling demand for climate action expressed through aggregate public opinion. As the ‘climate election’ framing gains prominence internationally [e.g. 68–72], this will be a significant caution to consider.

There are also questions about whether significant events following the 2019 election will prompt further change to aggregate public opinion about climate change in Australia. The public debate about Australia’s 2019–2020 bushfires clearly implicated climate change in the broader bushfire narrative [10], though the 2019 loss of the ‘climate election’ also underpinned counter-narratives [e.g. 73]. This is a notable shift given public debate about Australia’s devastating Black Saturday fires in 2009 largely ignored climate change [74]. The influence of experiencing extreme weather on attitudes about climate change carries mixed evidence, generally indicating at most a small impact [44, 75, 76]. However, it is perhaps unprecedented to have not only fires of the ferocity and spatial and temporal extent of Australia’s in the 2019–2020 summer, but for those to be accompanied by clear scientific statements on climate influence [77] and powerful institutional acknowledgement of the role of climate change [78].

Just as our results have shown that climate change is not a monolithic issue in voters’ political decisions, any impact of the 2019–2020 bushfires on voters’ attitudes will occur with the broader social context, made eminently more complex due to the impact of COVID-19. Some polling undertaken in 2020 finds, for the first time since 2012, a small decrease in the proportion of Australians supporting urgent action on climate change [16]. These findings implicate COVID-19 and the widespread disruption it has caused in the dip to concern about climate change, aligning with other research findings [79].

## Conclusion

We analysed the determinants of Australian voters’ attitudes toward climate change in the context of the 2019 federal election. We found a large majority of Australians consider emissions reduction important, but *how* important is consistently affected by political party preference and educational attainment. For climate policy in Australia, the key implication of the present research is that Australian political leaders could pursue a climate action agenda knowing that the majority of the voting population is likely to broadly support this course of action, regardless of which party is in power. Our findings suggest that support for strong policy action may be limited by voters’ preparedness to incur personal costs, especially among conservative voters. This highlights the importance of the stated positions on climate action of conservative political actors, as political leadership is an important influence on public opinion [39, 42]. Divisive politics that promote climate delay, familiar in the Australian political context [1, 6], may have a limited shelf life as the majority of voters, at the aggregate national level, accept the need for climate action.

## Supporting information

S1 File (PDF)

S1 Data (XLSX)
