# Supplementary material for: Australian voters’ attitudes to climate action and their social-political determinants
Source: PLoS One. 2021 Mar 24;16(3):e0248268. doi: 10.1371/journal.pone.0248268 (PMC7990191; doi:10.1371/journal.pone.0248268)
Supplement: S1 File — (PDF) [file pone.0248268.s001.pdf]

Supporting information: Australian voters' attitudes to climate action and their social-political determinants

A: Survey instrument

SM Table 1: Survey instrument, including exact phrasing of all questions and all response options.

Q1. In your view, how important or unimportant is it that Australia takes action to reduce greenhouse gas emissions in order to help limit future climate change?	
-99	Refused
-98	Don't know
1	Extremely important
2	Important
3	Unimportant
4	Extremely unimportant
Q2. In your view, which energy source or mix of energy sources should provide Australia's electricity in 2050?	
-99	Refused
-98	Don't know
1	All fossil fuels (coal and gas)
2	A relatively even mixture of fossil fuels and renewables
3	Mostly renewables, with some fossil fuels
4	All renewables
5	All nuclear power
Q3. To what extent are you prepared to accept a personal cost in order to support action to reduce Australia's emissions?	
-99	Refused
-98	Don't know
1	I am prepared to accept a significant personal cost in the interest of reduced emissions
2	I am prepared to accept a small personal cost in the interest of reduced emissions
3	I am not prepared to accept any personal cost in the interest of reduced emissions, but I think others should
4	I am not prepared to accept any personal cost in the interest of reduced emissions, and I think others should not either
Q4. How much did the issue of climate change influence your vote in the 2019 Federal election? For you personally, would you say climate change was ... ?	
-99	Refused
-98	Don't know
1	The most important issue
2	An important issue
3	Not very important
4	Not at all important
5	Did not consider climate change when voting
6	Did not vote
Q5 In the Federal election for the House of Representatives on Saturday 18 May, which party did you vote for first in the House of Representatives?	
-99	Refused
-98	Don't know
-97	Did not vote
-96	Not eligible to vote
1	Liberal
2	Labor

3	National
4	Greens
5	Other party (please specify)
6	No party
7	Australian Democrats
8	Christian Democratic Party
9	Citizens Electoral Council
10	Family First Party
11	Pauline Hanson's One Nation
12	Republican Party (replaced by Republican Party of Australia)
13	Shooters, Fishers and Farmers Party
14	Fishing Party
15	United Australia Party (formerly Palmer's United Party)
16	Katter's Australia Party
17	Liberal Democrats
18	Motoring Enthusiasts Party
19	Australian Sports Party (dissolved in 2015)
20	Reason Party (formerly The Australian Sex Party)
21	The Wikileaks Party (dissolved in 2015)
22	Australian Christians
23	Derryn Hinch's Justice Party
24	Centre Alliance (formerly Nick Xenophon Team)
25	Rise Up Australia
26	Science Party
27	Australian Liberty Alliance
28	Pirate Party
29	Citizens Electoral Council
30	Jacquie Lambie Network
31	Arts Party
32	Animal Justice Party
33	Australian Cyclists Party
34	Health Australia Party
35	Affordable Housing Party
36	Australia First Party
37	Australian Better Families
38	Australian Conservatives
39	Australian People's Party
40	Australian Progressives
41	Australian Workers Party
42	Child Protection Party
43	Climate Action! Immigration Action! Accountable Politicians!
44	Country Liberals (NT)
45	Democratic Labour Party
46	Fraser Anning's Conservative National Party
47	Help End Marijuana Prohibition (HEMP) Party
48	Independents For Climate Action Now
49	Involuntary Medication Objectors (Vaccination/Fluoride) Party
50	Labour DLP
51	Liberal National Party of Queensland
52	Love Australia or Leave
53	Non-Custodial Parents Party (Equal Parenting)
54	Secular Party of Australia
55	Seniors United Party of Australia

56	Socialist Alliance
57	Socialist Equality Party
58	Sustainable Australia
59	The Australian Mental Health Party
60	The Great Australian Party
61	The Small Business Party
62	The Together Party
63	Victorian Socialists
64	VOTEFLUX.ORG | Upgrade Democracy!
65	WESTERN AUSTRALIA PARTY
66	Yellow Vest Australia
97	Independent
Q6. Which party did you vote for in the Senate election?	
-99	Refused
-98	Don't know
-97	Did not vote
-96	Not eligible to vote
1	Liberal
2	Labor
3	National
4	Greens
5	Other party (please specify)
6	No party
7	Australian Democrats
8	Christian Democratic Party
9	Citizens Electoral Council
10	Family First Party
11	Pauline Hanson's One Nation
12	Republican Party (replaced by Republican Party of Australia)
13	Shooters, Fishers and Farmers Party
14	Fishing Party
15	United Australia Party (formerly Palmer's United Party)
16	Katter's Australia Party
17	Liberal Democrats
18	Motoring Enthusiasts Party
19	Australian Sports Party (dissolved in 2015)
20	Reason Party (formerly The Australian Sex Party)
21	The Wikileaks Party (dissolved in 2015)
22	Australian Christians
23	Derryn Hinch's Justice Party
24	Centre Alliance (formerly Nick Xenophon Team)
25	Rise Up Australia
26	Science Party
27	Australian Liberty Alliance
28	Pirate Party
29	Citizens Electoral Council
30	Jacquie Lambie Network
31	Arts Party
32	Animal Justice Party
33	Australian Cyclists Party
34	Health Australia Party
35	Affordable Housing Party
36	Australia First Party

37	Australian Better Families
38	Australian Conservatives
39	Australian People's Party
40	Australian Progressives
41	Australian Workers Party
42	Child Protection Party
43	Climate Action! Immigration Action! Accountable Politicians!
44	Country Liberals (NT)
45	Democratic Labour Party
46	Fraser Anning's Conservative National Party
47	Help End Marijuana Prohibition (HEMP) Party
48	Independents For Climate Action Now
49	Involuntary Medication Objectors (Vaccination/Fluoride) Party
50	Labour DLP
51	Liberal National Party of Queensland
52	Love Australia or Leave
53	Non-Custodial Parents Party (Equal Parenting)
54	Secular Party of Australia
55	Seniors United Party of Australia
56	Socialist Alliance
57	Socialist Equality Party
58	Sustainable Australia
59	The Australian Mental Health Party
60	The Great Australian Party
61	The Small Business Party
62	The Together Party
63	Victorian Socialists
64	VOTEFLUX.ORG | Upgrade Democracy!
65	WESTERN AUSTRALIA PARTY
66	Yellow Vest Australia
97	Independent
Can you please tell me which state or territory you live in?	
1	NSW
2	VIC
3	QLD
4	SA
5	WA
6	TAS
7	NT
8	ACT
Capital city / rest of state	
-97	Unable to establish
1	Capital City
2	Rest of State
Capital city / rest of state by state	
-97	Unable to establish
1	Greater Sydney
2	Rest of NSW
3	Greater Melbourne
4	Rest of Vic.
5	Greater Brisbane
6	Rest of Qld
7	Greater Adelaide

8	Rest of SA
9	Greater Perth
10	Rest of WA
11	Greater Hobart
12	Rest of Tas.
13	Greater Darwin
14	Rest of NT
15	Australian Capital Territory
Socio-Economic Indexes for Areas [*Note: This variable is the respondent's residence in a neighbourhood categorised by presence of extent of local socio-economic disadvantage. I.e., not a measure of disadvantage for the respondent, but of their residence in an area with disadvantage*.]	
-97	Unable to establish
1	Quintile 1 - Most disadvantage
2	Quintile 2
3	Quintile 3
4	Quintile 4
5	Quintile 5 - Least disadvantage
What is your gender?	
-99	Refused
-98	Don't know
-97	Not stated / Unknown
1	Male
2	Female
3	Other
Age as of 31st July 2019	
Numeric value	
-97	Unknown
Age group as of 31st July 2019	
-97	Unknown
1	18-24 years
2	25-34 years
3	35-44 years
4	45-54 years
5	55-64 years
6	65-74 years
7	75 or more years
Respondent country of birth grouping	
-99	Refused
-98	Don't know
-97	Not stated / Unknown
1	Australian born
2	Mainly Non-English speaking background
3	Mainly English speaking background
Are you an Australian citizen?	
-99	Refused
-98	Don't know
-97	Not stated / Unknown
1	Yes
2	No
Do you speak a language other than English at home?	
-99	Refused
-98	Don't know
-97	Not stated / Unknown

1	Yes
2	No
What is the total of all income you usually receive?	
-99	Refused
-98	Don't know
-97	Not stated / Unknown
1	$156,000 or more per year ($3,000 or more per week)
2	$104,000 to $155,999 per year ($2,000 - $2,999 per week)
3	$91,000 to $103,999 per year ($1,750 - $1,999 per week)
4	$78,000 to $90,999 per year ($1,500 - $1,749 per week)
5	$65,000 to $77,999 per year ($1,250 - $1,499 per week)
6	$52,000 to $64,999 per year ($1,000 - $1,249 per week)
7	$41,600 to $51,999 per year ($800 - $999 per week)
8	$33,800 to $41,599 per year ($650 - $799 per week)
9	$26,000 - $33,799 per year ($500 - $649 per week)
10	$20,800 to $25,999 per year ($400 - $499 per week)
11	$15,600 to $20,799 per year ($300 - $399 per week)
12	$7,800 to $15,599 per year ($150 - $299 per week)
13	Less than $7,800 per year ($1 - $149 per week)
14	Nil
15	Negative income
Are you of Aboriginal or Torres Strait Islander origin?	
-99	Refused
-98	Don't know
-97	Not stated / Unknown
1	Yes; Aboriginal
2	Yes; Torres Strait Islander
3	Yes; both
4	No
Highest educational qualification	
-97	Not stated / Unknown
1	Postgraduate Degree Level
2	Graduate Diploma and Graduate Certificate Level
3	Bachelor Degree Level
4	Advanced Diploma and Diploma Level
5	Certificate III & IV Level
6	Secondary Education - Year 12
7	Secondary Education - Years 10 and 11
8	Certificate I & II Level
9	Secondary Education - Years 9 and below

B: Extended methods: data analysis

All analyses were performed using R (R Core Team, 2013).

Descriptive summaries of response variables

First, responses to each question were summarised into descriptive tables (SM Tables 3 – 6) and charts (SM Figures 15 –18). An example of the development of these descriptives is presented in SM Box 1.

SM Box 1: Example of preparation of descriptive summaries of each variable.

```
#### Descriptives ANU Q1 ####
#How important for Australia to reduce GHG emissions to limit future climate
change?

# Extremely important (1)
ghg.eximp <- length(which(dat$ANU_Q1 == 1))
ghg.eximp.p <- (ghg.eximp / length(dat$ANU_Q1)) * 100
# Important (2)
ghg.imp <- length(which(dat$ANU_Q1 == 2))
ghg.imp.p <- (ghg.imp / length(dat$ANU_Q1)) * 100
# Unimportant (3)
ghg.unimp <- length(which(dat$ANU_Q1 ==3))
ghg.unimp.p <- (ghg.unimp / length(dat$ANU_Q1)) * 100
# Extremely unimportant (4)
ghg.exunimp <- length(which(dat$ANU_Q1 ==4))
ghg.exunimp.p <- (ghg.exunimp / length(dat$ANU_Q1)) * 100
# Don't know / not sure (98)
ghg.dk <- length(which(dat$ANU_Q1 == 98))
ghg.dk.p <- (ghg.dk / length(dat$ANU_Q1)) * 100
# Refused / prefer not to say (99)
ghg.ref <- length(which(dat$ANU_Q1 == 99))
ghg.ref.p <- (ghg.ref / length(dat$ANU_Q1)) * 100

# Combined important
ghg.imp.all <- ghg.eximp + ghg.imp
ghg.imp.all.p <- (ghg.imp.all / length(dat$ANU_Q1)) * 100

#Combined unimportant
ghg.unimp.all <- ghg.unimp + ghg.exunimp
ghg.unimp.all.p <- (ghg.unimp.all / length(dat$ANU_Q1)) * 100

### Plot Q1 - importance of GHG reduction
## Create data frame to hold summarised data from which to build plots

# 4 cateogies (i.e. not aggregated)
ghg.imp.df=data.frame(Opinion=c("Extremely important", "Important",
"Unimportant", "Extremely unimportant"), Count=c(ghg.eximp, ghg.imp, ghg.unimp,
ghg.exunimp))
ghg.imp.df$Opinion <- factor(ghg.imp.df$Opinion, levels = c("Extremely
important", "Important", "Unimportant", "Extremely unimportant"), ordered=TRUE)

ghg.imp.p.df=data.frame(Opinion=c("Extremely important", "Important",
"Unimportant", "Extremely unimportant"), Percent=c(ghg.eximp.p, ghg.imp.p,
ghg.unimp.p, ghg.exunimp.p))
ghg.imp.p.df$Opinion <- factor(ghg.imp.p.df$Opinion, levels = c("Extremely
important", "Important", "Unimportant", "Extremely unimportant"), ordered=TRUE)

# 2 categories (i.e. aggregated)
ghg.imp.all.df=data.frame(Opinion=c("All important", "All Unimportant"),
Count=c(ghg.imp.all, ghg.unimp.all))
ghg.imp.all.df$Opinion <- factor(ghg.imp.all.df$Opinion, levels = c("All
important", "All Unimportant"), ordered=TRUE)

ghg.imp.all.p.df=data.frame(Opinion=c("All important", "All Unimportant"),
Percent=c(ghg.imp.all.p, ghg.unimp.all.p))
ghg.imp.all.p.df$Opinion <- factor(ghg.imp.all.p.df$Opinion, levels = c("All
important", "All Unimportant"), ordered=TRUE)


# Create bar plot - counts - 4 cats
ghg.imp.barplot <- ggplot(ghg.imp.df, aes(x=Opinion, y=Count, fill=Opinion)) +
geom_bar(stat = "identity") +
  scale_fill_viridis_d() +
  theme(axis.text.x=element_text(angle=45, hjust=1)) +
  theme(legend.position = "none") +
  coord_cartesian(ylim=c(0, 1500)) +
  labs(x = "Opinion", y = "Count") +
```

```
  theme(axis.line = element_line(colour = "black"),
        panel.grid.major = element_blank(),
        panel.grid.minor = element_blank(),
        panel.background = element_blank())

# Create bar plot - counts - 2 cats
ghg.imp.all.barplot <- ggplot(ghg.imp.all.df, aes(x=Opinion, y=Count,
fill=Opinion)) + geom_bar(stat = "identity") +
  scale_fill_viridis_d() +
  theme(axis.text.x=element_text(angle=45, hjust=1)) +
  theme(legend.position = "none") +
  coord_cartesian(ylim=c(0, 1500)) +
  labs(x = "Opinion", y = "Count") +
  theme(axis.line = element_line(colour = "black"),
        panel.grid.major = element_blank(),
        panel.grid.minor = element_blank(),
        panel.background = element_blank())

# Create bar plot - percentages - 4 cats
ghg.imp.p.barplot <- ggplot(ghg.imp.p.df, aes(x=Opinion, y=Percent,
fill=Opinion)) + geom_bar(stat = "identity") +
  scale_fill_viridis_d() +
  theme(axis.text.x=element_text(angle=45, hjust=1)) +
  theme(legend.position = "none") +
  coord_cartesian(ylim=c(0, 100)) +
  labs(x = "Importance of Australia reducing greenhouse gas emissions", y =
"Percent") +
  theme(axis.line = element_line(colour = "black"),
        panel.grid.major = element_blank(),
        panel.grid.minor = element_blank(),
        panel.background = element_blank())

# Create bar plot - percentages - 2 cats
ghg.imp.all.p.barplot <- ggplot(ghg.imp.all.p.df, aes(x=Opinion, y=Percent,
fill=Opinion)) + geom_bar(stat = "identity") +
  scale_fill_viridis_d() +
  theme(axis.text.x=element_text(angle=45, hjust=1)) +
  theme(legend.position = "none") +
  coord_cartesian(ylim=c(0, 100)) +
  labs(x = "Opinion", y = "Percent") +
  theme(axis.line = element_line(colour = "black"),
        panel.grid.major = element_blank(),
        panel.grid.minor = element_blank(),
        panel.background = element_blank())
```

Ordinal logistic regression on ordinal dependent variables (Qs 1, 3, 4)

Next, ordinal logistic regression (OLR) was conducted on all ordinal questions (Qs 1, 3, 4) using *plor* in the *MASS* package. Ordinal logistic regression is an approach for analysing ordinal data that neither assumes equal intervals (as in the case of using standard parametric analyses for Likert-type data) nor treats the ordered categories simply as categories (as in the case of using more common analysis techniques such as ANOVA or chi-squared test of independence). Accordingly, it is the correct analysis to use for data such as ours, which contain valuable information both with regard to the differences in magnitude between the categories, and the order of the categories (Liddell and Kruschke, 2018). For example, OLR has been applied extensively by Tranter's research on climate opinion in Australia (Tranter, 2011; Tranter, 2013; Tranter, 2014; Tranter and Booth, 2015; Tranter, 2017; Tranter, 2019; Tranter and Foxwell-Norton, 2020; Tranter et al., 2020).

The OLR produces two key output values of interest, the odds ratio and its p-value. First, the odds ratio is a value that describes a) the extent to which an increase in a **continuous** independent variable predicts an increase in a dependent variable, or b) whether the difference between a baseline category of a **categorical** independent variable and another category of that same categorical variable predicts

an increase in the dependent variable. The p-value relates to a null hypothesis in which the odds ratio is equal to 1 (in other words, the null hypothesis tells us there is no relationship between the variables).

The OLR can be used with both continuous and categorical independent variables, however the nature of interpretation differs between the two data types. Dependent variables are always ordinal.

For continuous independent variables, the odds ratio describes the odds of an increase in the dependent variable based on a *one unit increase* in the independent variable. For example, if the independent continuous variable is age measured in units of 1 year, then an odds ratio of 1.1 indicates that the dependent variable is 1.1 times *more likely to be a higher value* for each additional 1 year of age. Multiple unit increases in the continuous independent variable therefore can be measured by taking the odds ratio to the power of the number of intervals. So, again using the age example, an odds ratio of 1.1 for a 1 year unit increase can be taken to the power of 10 to identify the odds ratio of an increase in the dependent variable based on a 10 year increase in age ($OR_{1\ year}$ 1.1 ^ 10 = $OR_{10\ years}$ 2.54).

For categorical independent variables, the odds ratio describes the odds of an increase in the dependent variable based on the difference between a *baseline category* and the category of comparison. All categorical independent variables therefore have one category selected as the baseline, and all other categories are compared against this baseline. For example, if 'male' is selected as the default category in a case where gender is represented as binary, then the odds ratio will indicate the likelihood of an increase in the dependent variable based on the difference between females compared to males (e.g. OR_{female} = 2.5 indicates females are 2.5 times more likely than males to provide a higher response on the dependent variable). In a case where gender is represented as more than two categories and male is selected as the baseline category, then all other categories (e.g. female, non-binary, intersex) are compared to male. Each non-male category therefore has its own odds ratio comparing that category to the male, baseline category. In treatment-control experiments, the baseline will be the control against which the treatment is compared. In observational studies, such as the present research, the baseline will be whichever category is listed first in the variable. However, whenever possible the baseline should ideally be selected according to theoretical logic. OLR with categorical independent variables can be repeated with different baselines, producing odds ratios of differing values due to the changing baseline for comparison. However, although the odds ratios may change, the absolute differences will remain constant. As a result, especially when a baseline variable must be selected somewhat arbitrarily, it is necessary to emphasise the comparison between the categories in interpretation of the data (i.e. $OR_{females}$ 2.5 compared to male baseline), rather than presenting the odds ratio as a value out of its comparative context.

We ran the OLR analysis in two stages (following Park et al., 2015). First, a series of univariate analyses for each dependent variable, and second, a combined, multivariate analysis:

- Univariate analyses examined each independent variable for its predictive value on each of the dependent variables taking no account for the influence of other independent variables (i.e. without the presence of the other variables).
- Multivariate analysis then selected all independent variables that were significant under univariate analysis and built a multivariate model to examine the significance of each of these independent variables in the presence of each other. The multivariate analysis explains what are the most influential independent variables on the dependent variable, taking into account covariance between the multiple independent variables. The independent variables that were significant under univariate analysis but no longer significant under multivariate analysis are explained by high covariance between the independent variables, with the multivariate model retaining significance of only the most influential of the independent variables with covariance.

To prepare the data for OLR analysis, we first converted the responses into ordinal, categorical, or continuous variables. In OLR, dependent variables can be ordinal, but independent variables must be categorical or continuous. For each ordinal variable, we converted the response data to ordered factors. For each non-ordinal categorical variable, we converted the response data to named categories. For continuous variables, we converted the data to numeric values. We treated three ordinal independent variables as continuous, rather than categorical, in the analysis (SEIFA (social disadvantage index), 5 levels; income, 15 levels; education, 9 levels) as this proved more useful than treating the variables as un-ordered categories. But, we recognise this creates limitations in terms of treating ordinal variables as interval data. In our view, this was the most useful compromise given OLR cannot accommodate ordinal independent variables. For all question types, we changed non-ordered response values to NA and excluded them from the analysis (e.g. refused to answer, don't know, response values with too few responses for inclusion in the analysis). Example of data conversion for OLR is presented in SM Box 2.

SM Box 2: Example of data conversion for OLR, for both SEIFA (social disadvantage index) & climate Q1 (importance of GHG reductions).

```
levels(dat$p_seifa_m)[levels(dat$p_seifa_m)==-97] <-  NA
levels(dat$p_seifa_m)[levels(dat$p_seifa_m)==1] <- "Q1"
levels(dat$p_seifa_m)[levels(dat$p_seifa_m)==2] <- "Q2"
levels(dat$p_seifa_m)[levels(dat$p_seifa_m)==3] <- "Q3"
levels(dat$p_seifa_m)[levels(dat$p_seifa_m)==4] <- "Q4"
levels(dat$p_seifa_m)[levels(dat$p_seifa_m)==5] <- "Q5"
dat$p_seifa_m <- factor(dat$p_seifa_m, levels = c("Q5","Q4","Q3","Q2","Q1"),
ordered=TRUE)

dat$ANU_Q1_m <- as.factor (dat$ANU_Q1)
levels(dat$ANU_Q1_m)[levels(dat$ANU_Q1_m)==-98] <- NA
levels(dat$ANU_Q1_m)[levels(dat$ANU_Q1_m)==-99] <- NA
levels(dat$ANU_Q1_m)[levels(dat$ANU_Q1_m)==1] <- "Extremely important"
levels(dat$ANU_Q1_m)[levels(dat$ANU_Q1_m)==2] <- "Important"
levels(dat$ANU_Q1_m)[levels(dat$ANU_Q1_m)==3] <- "Unimportant"
levels(dat$ANU_Q1_m)[levels(dat$ANU_Q1_m)==4] <- "Extremely unimportant"
# Order such that higher values = more importance for GHG reduction (more
intuituve interpretation)
dat$ANU_Q1_m <- factor(dat$ANU_Q1_m, levels = c("Extremely unimportant",
"Unimportant", "Important", "Extremely important"), ordered=TRUE)
```

Our OLR analyses used the following independent variables:

- SEIFA (social disadvantage): continuous (most disadvantage → least disadvantage)
- Income: continuous (least income → most income)
- Age: continuous (youngest → oldest)
- Education: continuous (least education → most education)
- Gender: categorical (male, female)
- State of residence: categorical (NSW, VIC, QLD, SA, WA, TAS, NT, ACT)
- Residence in capital city v. regional area: categorical (Capital city, rest of state)
- Country of birth: categorical (Australian born, mainly non-English speaking background, mainly English speaking background)
- Main language spoken at home: categorical (English only, language other than English)
- First preference in the House of Representatives at the May 2019 federal election: categorical (Liberal Party, Australian Labor Party, The Greens, Liberal-National party (QLD only), The Nationals)

To recap, for continuous independent variables, OLR provides a result that indicates the impact of a one unit increase in the independent variable on the likelihood of an increase in the ordinal categories of the dependent variable. For categorical independent variables, OLR provides a visually similar

output, but it differs in the nature of interpretation. In analysis of categorical independent variables in OLR, the first listed category is selected as a 'baseline' against which all other categories are compared. Therefore, for comparison across multiple (3 or more) categories within a categorical variable, the OLR must be run multiple times with each category selected as the baseline for one of the runs.

In the univariate OLR analysis, we examined the odds ratio and the p-value of the odds ratio to determine whether there was an influence of the independent variable on the dependent variable. As the OLR is a logistic method, we take the exponent of the coefficient to determine the odds ratio. SM Box 3 presents an example of the univariate OLR in R.

SM Box 3: Example of univariate OLR examining influence of gender on Q1 (importance of GHG reduction) responses.

```
Q1.gen.results <- round(Q1.gen.coef[1,], digits=4)
# OLR for ANU_Q1 predicted by gender ####
#Run OLR model
Q1.gen <- polr (ANU_Q1_m ~ p_gender, data=dat, Hess=T)
# summary(Q1.gen)
# To get p-values: store coeffiencts
Q1.gen.coef <- coef(summary(Q1.gen))
## calculate and prepare to store p values
Q1.gen.p <- (pnorm(abs(Q1.gen.coef[, "t value"]), lower.tail = FALSE) * 2)
## Calculate and prepare to store odds ratios
Q1.gen.or1 <- (exp(Q1.gen.coef[, "Value"]))
## Calculate and prepare to store inverse odds ratios
Q1.gen.or2 <- (1/(exp(Q1.gen.coef[, "Value"])))
## Calculate and prepare to store confidence interval for odds ratios (not
inverse) IGNORE FOR CUTPOINTS
Q1.gen.orci <-  exp(confint(Q1.gen, level = 0.95))
Q1.gen.ci25 <- Q1.gen.orci["2.5 %"]
Q1.gen.ci975 <- Q1.gen.orci["97.5 %"]
## Calculate and prepare to store CI for INVERSE odds radios IGNORE FOR CUTPOINTS
Q1.gen.iorci <- 1/(exp(confint(Q1.gen, level = 0.95)))
Q1.gen.ici25 <- Q1.gen.iorci["2.5 %"]
Q1.gen.ici975 <- Q1.gen.iorci["97.5 %"]
## combined table w stored values
Q1.gen.coef <- cbind(Q1.gen.coef, "p value" = Q1.gen.p)
Q1.gen.coef <- cbind(Q1.gen.coef, "Odds ratio" = Q1.gen.or1)
Q1.gen.coef <- cbind(Q1.gen.coef, "Inverse odds ratio" = Q1.gen.or2)
Q1.gen.coef <- cbind(Q1.gen.coef, "CI 2.5% OR" = Q1.gen.ci25)
Q1.gen.coef <- cbind(Q1.gen.coef, "CI 97.5% OR" = Q1.gen.ci975)
Q1.gen.coef <- cbind(Q1.gen.coef, "CI 2.5% Inv. OR" = Q1.gen.ici25)
Q1.gen.coef <- cbind(Q1.gen.coef, "CI 97.5% Inv. OR" = Q1.gen.ici975)
#Remove intercept/cutpoints as irrelevant to interpretation and reporting, save
variable of interest in vector
Q1.gen.results <- round(Q1.gen.coef[1,], digits=4)
Q1.gen.results
             Value         Std. Error           t value            p value
            0.5179             0.0839            6.1737             0.0000
        Odds ratio Inverse odds ratio        CI 2.5% OR        CI 97.5% OR
            1.6785             0.5958            1.4244             1.9790
   CI 2.5% Inv. OR   CI 97.5% Inv. OR
            0.7021             0.5053
```

For each ordinal dependent variable (Qs 1, 3, 4), we then selected all independent variables that yielded a significant p-value (at $\alpha = 0.05$) in the univariate analyses for inclusion in a multivariate OLR model. SM Box 4 presents an example of the multivariate OLR model.

SM Box 4: Example of the multivariate OLR model, incorporating all variables found to be significant in the univariate analysis.

```
#### MULTIVARIATE OLR Q1 FOR ALL VARIABLES SIGNIFICANT AS UNIVARATE ####
Q1.multi <- polr (ANU_Q1_m ~ p_age + p_gender + p_education_m2 + p_state_m +
p_cob_group_m + p_seifa_m2 + as.factor(CSES_Q15_mx), data=dat, Hess=T,
method=c("probit"))
summary(Q1.multi)
# To get p-values: store coeffiencts
Q1.multi.coef <- coef(summary(Q1.multi))
## calculate and prepare to store p values
Q1.multi.p <- (pnorm(abs(Q1.multi.coef[, "t value"]), lower.tail = FALSE) * 2)
## Calculate and prepare to store odds ratios
Q1.multi.or1 <- (exp(Q1.multi.coef[, "Value"]))
## Calculate and prepare to store confidence interval for odds ratios (not
inverse) IGNORE FOR CUTPOINTS
Q1.multi.orci <-  exp(confint(Q1.multi, level = 0.95))
Q1.multi.ci25 <- Q1.multi.orci[,"2.5 %"]
Q1.multi.ci975 <- Q1.multi.orci[,"97.5 %"]
## combined table w stored values
Q1.multi.coef <- cbind(Q1.multi.coef, "p value" = Q1.multi.p)
Q1.multi.coef <- cbind(Q1.multi.coef, "Odds ratio" = Q1.multi.or1)
Q1.multi.coef <- cbind(Q1.multi.coef, "CI 2.5% OR" = Q1.multi.ci25)
Q1.multi.coef <- cbind(Q1.multi.coef, "CI 97.5% OR" = Q1.multi.ci975)
#Remove intercept/cutpoints as irrelevant to interpretation and reporting, save
variable of interest in vector
Q1.multi.results <- round(Q1.multi.coef[1:17,], digits=4)
Q1.multi.results
                                      Value Std. Error t value
p_age                               -0.0018     0.0019 -0.9567
p_gender                             0.3049     0.0595  5.1278
p_education_m2                       0.0470     0.0145  3.2463
p_state_mVIC                        -0.0682     0.0826 -0.8252
p_state_mQLD                        -0.1611     0.0932 -1.7281
p_state_mSA                          0.0139     0.1143  0.1215
p_state_mWA                         -0.0018     0.1070 -0.0169
p_state_mTAS                        -0.2007     0.2009 -0.9991
p_state_mNT                          0.1681     0.4414  0.3809
p_state_mACT                         0.1307     0.2144  0.6099
p_cob_group_mMainly NESB background -0.0171     0.0914 -0.1873
p_cob_group_mMainly ESB background   0.1090     0.0996  1.0943
p_seifa_m2                          -0.0348     0.0226 -1.5427
as.factor(CSES_Q15_mx)ALP            0.8328     0.0699 11.9075
as.factor(CSES_Q15_mx)Nat           -0.1554     0.1387 -1.1202
as.factor(CSES_Q15_mx)Grn            1.4953     0.1139 13.1231
as.factor(CSES_Q15_mx)LNP           -0.1011     0.1520 -0.6653
                                    p value Odds ratio
p_age                                0.3387     0.9982
p_gender                             0.0000     1.3565
p_education_m2                       0.0012     1.0482
p_state_mVIC                         0.4093     0.9341
p_state_mQLD                         0.0840     0.8512
p_state_mSA                          0.9033     1.0140
p_state_mWA                          0.9865     0.9982
p_state_mTAS                         0.3177     0.8181
p_state_mNT                          0.7033     1.1831
p_state_mACT                         0.5419     1.1397
p_cob_group_mMainly NESB background  0.8514     0.9830
p_cob_group_mMainly ESB background   0.2738     1.1151
p_seifa_m2                           0.1229     0.9658
as.factor(CSES_Q15_mx)ALP            0.0000     2.2997
as.factor(CSES_Q15_mx)Nat            0.2626     0.8561
as.factor(CSES_Q15_mx)Grn            0.0000     4.4605
as.factor(CSES_Q15_mx)LNP            0.5059     0.9038
                                    CI 2.5% OR CI 97.5% OR
p_age                                   0.9946      1.0019
p_gender                                1.2073      1.5242
p_education_m2                          1.0188      1.0784
```

```
p_state_mVIC                          0.7944      1.0983
p_state_mQLD                          0.7092      1.0220
p_state_mSA                           0.8110      1.2695
p_state_mWA                           0.8097      1.2316
p_state_mTAS                          0.5533      1.2166
p_state_mNT                           0.5119      2.9128
p_state_mACT                          0.7547      1.7509
p_cob_group_mMainly NESB background   0.8222      1.1764
p_cob_group_mMainly ESB background    0.9182      1.3566
p_seifa_m2                            0.9240      1.0095
as.factor(CSES_Q15_mx)ALP             2.0056      2.6382
as.factor(CSES_Q15_mx)Nat             0.6523      1.1237
as.factor(CSES_Q15_mx)Grn             3.5780      5.5941
as.factor(CSES_Q15_mx)LNP             0.6710      1.2176
```

Due to the nature of OLR analysis, we ran 13 replications of each of the three multivariate models. This relates to the inclusion of categorical independent variables with more than two levels. Because the OLR selects the first listed category as the baseline category (similar to a dummy variable), OLR results provide odds ratios for all other categories in that variable in relation to the baseline, but not to each other. The model results, as a whole, are insightful and robust, but the ability to draw conclusions about the differences between categories within the categorical variables are limited. As such, we elected to run the multivariate OLR models a number of times such that each category within categorical variables with two or more categories was selected as the baseline for one of the model runs. We included all variables selected for inclusion in the multivariate models (i.e. those significant in the univariate analyses) for these modified replications. We report in detail on only the first multivariate OLR model as this offers insight into the relationships between variables (results tables in SM Tables 7, 11, 15). However, we summarise comparisons between each of the categories via matrices reporting ORs and p-values in SM Tables 8-10, 12-14, 16-18.

We also prepared forest plots to visualise the results of the OLR analyses, however, we prepared these for the models with the default baseline only (rather than preparing a series of multiple forest plots for each unique potential baseline) (SM Figures 19-21). Forest plots are commonly used to illustrate the findings of meta-analyses, but are also suited to illustrating odds ratios and their confidence intervals. Due to the difference in nature of the OLR using continuous and categorical independent variables, we developed separate forest plots for the two independent data types to aid interpretation. SM Box 5 presents an example of the development of the forest plots.

SM Box 5: Example of the development of the forest plots for OLR using both continuous and categorical independent variables, and univariate and multivariate analyses.

```
# Build forest plot containing JUST continuous vars from univariate analysis ####
# Build data frame containing all OR and CI from Q1 - this can then be used for
creating the forestplot
Q1.cont.variables <- c("Age", "Education", "Income", "Disadvantage")
Q1.cont.variables <- factor(Q1.variables, ordered=TRUE)
Q1.cont.levels <- c("Age" ,"Education" ,"Income","Disadvantage")
Q1.cont.OR <- c(Q1.age.results["Odds ratio"],Q1.education.results["Odds
ratio"],Q1.income.results["Odds ratio"],Q1.seifa.results["Odds ratio"])
Q1.cont.25CI <- c(Q1.age.results["CI 2.5% OR"],Q1.education.results["CI 2.5%
OR"],Q1.income.results["CI 2.5% OR"],Q1.seifa.results["CI 2.5% OR"])
Q1.cont.975CI <- c(Q1.age.results["CI 97.5% OR"],Q1.education.results["CI 97.5%
OR"],Q1.income.results["CI 97.5% OR"],Q1.seifa.results["CI 97.5% OR"])
Q1.cont.pvalue <-  c(Q1.age.results["p value"],Q1.education.results["p
value"],Q1.income.results["p value"],Q1.seifa.results["p value"])

Q1.cont.summary <- data.frame(Q1.cont.variables, Q1.cont.levels, Q1.cont.OR,
Q1.cont.25CI, Q1.cont.975CI, Q1.cont.pvalue)

fp.Q1.cont <- ggplot(data=Q1.cont.summary, aes(x=Q1.cont.levels, y=Q1.cont.OR,
ymin=Q1.cont.25CI, ymax=Q1.cont.975CI)) +
  geom_pointrange() +
```

```
  geom_hline(yintercept=1, lty=2, colour="grey") +
  xlab("") + ylab("Odds ratio (95% CI)") +
  theme_bw() +
  theme(panel.grid.major = element_blank(), panel.grid.minor = element_blank()) +
  scale_y_log10(breaks=c(0.9,1,1.1), labels=c(0.9,1,1.1)) +
  coord_flip()
print(fp.Q1.cont)

# Build forest plot containing JUST categorical vars from univariate analysis
####
# Build data frame containing all OR and CI from Q1 - this can then be used for
creating the forestplot
Q1.cat.variables <- c("Political preference (Lib)", "Political preference (Lib)",
"Political preference (Lib)", "Political preference (Lib)", "Gender (male)",
"State (NSW)","State (NSW)","State (NSW)","State (NSW)","State (NSW)","State
(NSW)","State (NSW)", "Region (capital city)", "Country of birth (Australian
born)","Country of birth (Australian born)", "Language (English only)")
Q1.cat.variables <- factor(Q1.cat.variables, ordered=TRUE)
Q1.cat.levels <- c("ALP", "Nationals", "Greens", "LNP", "Female","VIC",
"QLD","SA","WA","TAS","NT","ACT","Non-capital city","Other, non-English
speaking","Other, English speaking","Language (non-English)")
Q1.cat.levels <- factor(Q1.cat.levels, levels=c("Language (non-English)", "Other,
non-English speaking","Other, English speaking","Non-capital
city","ACT","NT","TAS","WA","SA","QLD","VIC", "Female", "Nationals", "LNP",
"Greens", "ALP"), ordered=TRUE)
Q1.cat.OR <- c(Q1.hor.results[1:4,5], Q1.gen.results["Odds ratio"],
Q1.state.results[1:7,5],Q1.region.results["Odds
ratio"],Q1.cob_group.results[1:2,5],Q1.lote.results["Odds ratio"])
Q1.cat.25CI <- c(Q1.hor.results[1:4,7], Q1.gen.results["CI 2.5% OR"],
Q1.state.results[1:7,7],Q1.region.results["CI 2.5%
OR"],Q1.cob_group.results[1:2,7],Q1.lote.results["CI 2.5% OR"])
Q1.cat.975CI <- c(Q1.hor.results[1:4,8], Q1.gen.results["CI 97.5%
OR"],Q1.state.results[1:7,8],Q1.region.results["CI 97.5%
OR"],Q1.cob_group.results[1:2,8],Q1.lote.results["CI 97.5% OR"])
Q1.cat.pvalue <-  c(Q1.hor.results[1:4,4], Q1.gen.results["p value"],
Q1.state.results[1:7,4],Q1.region.results["p
value"],Q1.cob_group.results[1:2,4],Q1.lote.results["p value"])

Q1.cat.summary <- data.frame(Q1.cat.variables, Q1.cat.levels, Q1.cat.OR,
Q1.cat.25CI, Q1.cat.975CI, Q1.cat.pvalue)

fp.Q1.cat <- ggplot(data=Q1.cat.summary, aes(x=Q1.cat.levels, y=Q1.cat.OR,
ymin=Q1.cat.25CI, ymax=Q1.cat.975CI)) +
  geom_pointrange() +
  geom_hline(yintercept=1, lty=2, colour="grey") +
  xlab("") + ylab("Odds ratio (95% CI)") +
  theme_bw() +
  theme(panel.grid.major = element_blank(), panel.grid.minor = element_blank()) +
  scale_y_log10(breaks=c(0.5,1,3,10,30)) +
  coord_flip()
print(fp.Q1.cat)

# Build forest plot containing JUST continuous vars from multivariate analysis
####
# Build data frame containing all OR and CI from Q1 - this can then be used for
creating the forestplot
Q1.multi.cont.variables <- c("Age", "Education", "Disadvantage")
Q1.multi.cont.variables <- factor(Q1.multi.cont.variables, ordered=TRUE)
Q1.multi.cont.levels <- c("Age" ,"Education" ,"Disadvantage")
Q1.multi.cont.levels <- factor(Q1.multi.cont.levels,
levels=c("Disadvantage","Education","Age"), ordered=TRUE)
Q1.multi.cont.levels <- Q1.multi.cont.levels[!is.na(Q1.multi.cont.levels)]
Q1.multi.cont.OR <-
c(Q1.multi.results[1,5],Q1.multi.results[3,5],Q1.multi.results[13,5])
Q1.multi.cont.25CI <-
c(Q1.multi.results[1,5],Q1.multi.results[3,5],Q1.multi.results[13,5])
Q1.multi.cont.975CI <-
c(Q1.multi.results[1,5],Q1.multi.results[3,5],Q1.multi.results[13,5])
```

```
Q1.multi.cont.pvalue <-
c(Q1.multi.results[1,5],Q1.multi.results[3,5],Q1.multi.results[13,5])

Q1.multi.cont.summary <- data.frame(Q1.multi.cont.variables,
Q1.multi.cont.levels, Q1.multi.cont.OR, Q1.multi.cont.25CI, Q1.multi.cont.975CI,
Q1.multi.cont.pvalue)

fp.multi.cont.Q1 <- ggplot(data=Q1.multi.cont.summary,
aes(x=Q1.multi.cont.levels, y=Q1.multi.cont.OR, ymin=Q1.multi.cont.25CI,
ymax=Q1.multi.cont.975CI)) +
  geom_pointrange() +
  geom_hline(yintercept=1, lty=2, colour="grey") +
  xlab("") + ylab("Odds ratio (95% CI)") +
  theme_bw() +
  theme(panel.grid.major = element_blank(), panel.grid.minor = element_blank()) +
  scale_y_log10(breaks=c(0.98,1,1.02,1.04)) +
  coord_flip()
print(fp.multi.cont.Q1)

# Build forest plot containing JUST categorical vars from multivariate analysis
####
# Build data frame containing all OR and CI from Q1 - this can then be used for
creating the forestplot
Q1.multi.cat.variables <- c("Political preference (Lib)", "Political preference
(Lib)", "Political preference (Lib)", "Political preference (Lib)", "Gender
(male)", "State (NSW)","State (NSW)","State (NSW)","State (NSW)","State
(NSW)","State (NSW)","State (NSW)", "Country of birth (Australian born)","Country
of birth (Australian born)")
Q1.multi.cat.variables <- factor(Q1.multi.cat.variables, ordered=TRUE)
Q1.multi.cat.levels <- c("ALP", "Nationals", "Greens", "LNP", "Female","VIC",
"QLD","SA","WA","TAS","NT","ACT","Other, non-English speaking","Other, English
speaking")
Q1.multi.cat.levels <- factor(Q1.multi.cat.levels, levels=c("Other, non-English
speaking","Other, English speaking","ACT","NT","TAS","WA","SA","QLD","VIC",
"Female", "Nationals", "LNP", "Greens", "ALP"), ordered=TRUE)
Q1.multi.cat.levels <- Q1.multi.cat.levels[!is.na(Q1.multi.cat.levels)]
Q1.multi.cat.OR <-
c(Q1.multi.results[14:17,5],Q1.multi.results[2,5],Q1.multi.results[4:10,5],
Q1.multi.results[11:12,5])
Q1.multi.cat.25CI <-
c(Q1.multi.results[14:17,6],Q1.multi.results[2,6],Q1.multi.results[4:10,6],
Q1.multi.results[11:12,6])
Q1.multi.cat.975CI <-
c(Q1.multi.results[14:17,7],Q1.multi.results[2,7],Q1.multi.results[4:10,7],
Q1.multi.results[11:12,7])
Q1.multi.cat.pvalue <-
c(Q1.multi.results[14:17,4],Q1.multi.results[2,4],Q1.multi.results[4:10,4],
Q1.multi.results[11:12,4])

Q1.multi.cat.summary <- data.frame(Q1.multi.cat.variables, Q1.multi.cat.levels,
Q1.multi.cat.OR, Q1.multi.cat.25CI, Q1.multi.cat.975CI, Q1.multi.cat.pvalue)

fp.multi.cat.Q1 <- ggplot(data=Q1.multi.cat.summary, aes(x=Q1.multi.cat.levels,
y=Q1.multi.cat.OR, ymin=Q1.multi.cat.25CI, ymax=Q1.multi.cat.975CI)) +
  geom_pointrange() +
  geom_hline(yintercept=1, lty=2, colour="grey") +
  xlab("") + ylab("Odds ratio (95% CI)") +
  theme_bw() +
  theme(panel.grid.major = element_blank(), panel.grid.minor = element_blank()) +
  scale_y_log10(breaks=c(0.5,1,3,10,30)) +
  coord_flip()
print(fp.multi.cat.Q1)
```

ANOVA and chi-squared analysis on categorical variable (Q2)

Question 3 was a categorical question, including archetypes of energy mix preferences. A perfect question would have allowed participants to nominate what proportion of Australia's energy supply would be supplied by each energy type (fossil fuels, renewables, nuclear). However, we desired to keep the survey instrument streamlined and accessible to participants who would lack (or perceive that they lack) the knowledge to answer a more complicated question (i.e. involving specifying proportions across energy types to describe their preferred energy mix). As such, we presented response options that, in our view, reflected archetypical preferences for Australia's future energy supply promoted in civic, media, and political debate. Accordingly, the categorical response options for question 2 were (excluding don't know, refused):

- All fossil fuels (coal and gas)
- A relatively even mixture of fossil fuels and renewables
- Mostly renewables, with some fossil fuels
- All renewables
- All nuclear power

We analysed responses to this question against all demographic variables (as with Qs 1, 3, 4). We analysed categorical independent variables using a chi-square test of independence. We analysed continuous independent variables using an analysis of variance.

For the categorical independent variables, the analyses yield a p-value for the chi-square test of independence. If this p-value is significant at $\alpha = 0.05$, then we extracted the chi-square contingency table to view the residuals. We then noted the residuals with large differences from zero to identify the combination of characteristics and energy preferences that contributed most to the significant test result. These same combination of energy preferences (e.g. all renewables) and characteristics (e.g. female) are those that we determine have an association of statistical significance beyond chance.

For the continuous independent variables, the ANOVA yield a p-value for the test of differences of means between the groups. In this case, the groups are each energy preference option. The means, therefore, are the means of the continuous variables. When a significant p-value was returned (at $\alpha = 0.05$), we conducted Tuckey's post-hoc test to identify those groups that presented a statistically significant difference. We summarised results in SM Table 19.

Age and opinion projections

We prepared our simplified age projections using Australian Bureau of Statistics (ABS) population projections for 2017-2066 (Australian Bureau of Statistics, 2018b). These are official population growth projections (including future age profile) produced and published by the Australian Government. We used the age distribution of opinion on Q1 (importance of Australia reducing GHG emissions) and weighted this across the ABS population projections.

For this, from our results we took the proportion of each 5-year age bracket that selected 'extremely important' as their response to Q1. We then applied this proportion to the corresponding age bracket in the ABS population data, starting with 2019. Next, we examined how future trends in opinion, with specific regard to the proportion of the population that considers action on climate change to be 'extremely important', would change based on natural ageing, including entry and exit of voters. We did this by attaching the proportion of 'extremely important' on Q1 to the group in each age bracket, and then shifting that proportion with the group as it aged.

We prepared two scenarios:

- Future young generations are equally as concerned as current young generations

- Future young generations are more concerned than current young generations, with an increase per 5 years equivalent to slope coefficient from linear regression of existing age opinion gradient.

Based on the population projections, we then combined the proportion of each age bracket that considers climate change to be 'extremely important', weighted by the proportion of the total Australian population that age bracket represents.

We ran these simplified scenarios using Excel. The extended results below contain both the ABS population projections and our opinion future scenarios.

C: Extended results

Descriptive summaries of sample demographics

Summary charts describing our sample are included below in SM Figures 1 – 14. A comparative summary of our sample, the panel from which it was drawn, and the Australian population is presented in SM Table 2.

SM Table 2: Demographic summary of sample, including data for the panel, those panel members who participated in the survey, and comparative figures for the Australian population. Data provided by The Social Research Centre.

Subgroup	Panel composition (%)	Survey respondents (%)	Australian population comparison (%)
**Base (n)**	**2,839**	**2,033**	
**Gender**			
Male	46.6	47.0	49.1
Female	53.0	52.7	50.9
**Age**			
18-24 years	7.1	4.2	12.2
25-34 years	13.9	12.2	19.3
35-44 years	15.3	14.5	17.1
45-54 years	18.0	17.9	16.5
55-64 years	18.2	19.0	14.9
65-74 years	18.4	21.0	11.5
75+ years	8.9	11.0	8.7
Median age	N/A	55	45
**Location**			
Sydney	16.7	16.3	20.7
Rest of NSW	12.5	12.8	11.3
Melbourne	18.4	17.2	19.8
Rest of VIC	7.3	7.4	6.3
Brisbane	10.8	11.9	9.6
Rest of QLD	8.2	8.0	10.2
Adelaide	7.3	7.8	5.5
Rest of SA	1.3	1.7	1.6
Perth	9.3	9.2	8.1

Rest of WA	2.1	1.7	2.2
Hobart	1.4	1.2	0.9
Rest of TAS	1.2	1.2	1.2
Darwin	0.8	0.5	0.6
Rest of NT	0.2	0.1	0.4
ACT	2.4	2.8	1.7

Note: Australian population figures from the Australian Bureau of Statistics (2018a).

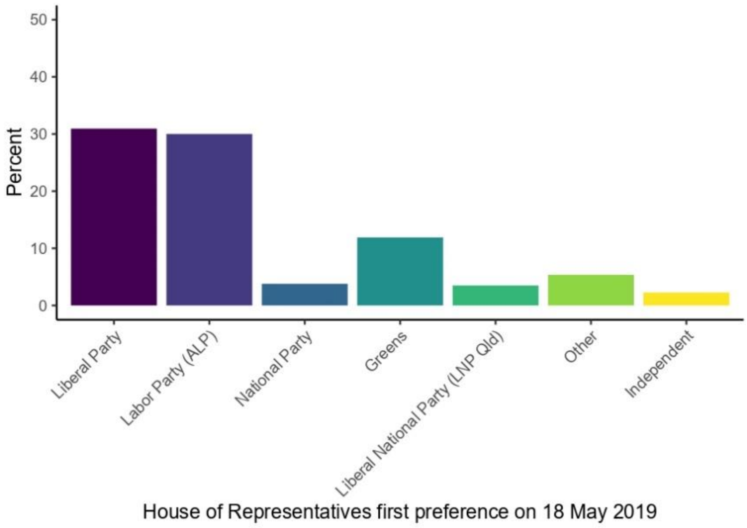

SM Figure 1: House of representative first preference vote.

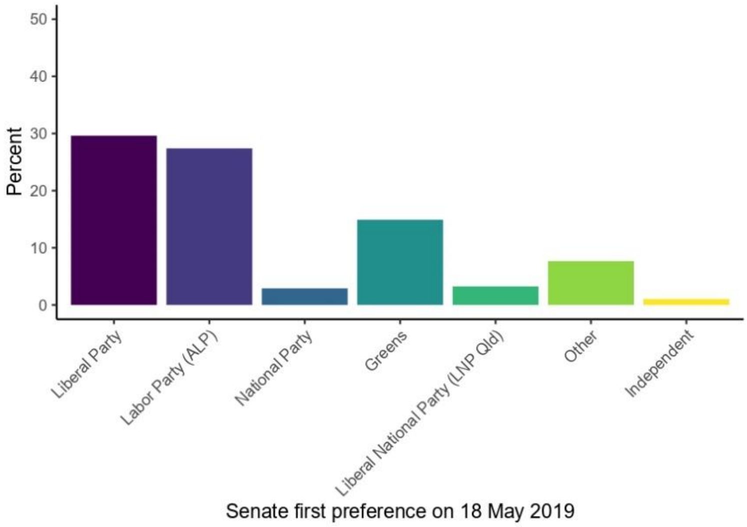

SM Figure 2: Senate first preference vote.

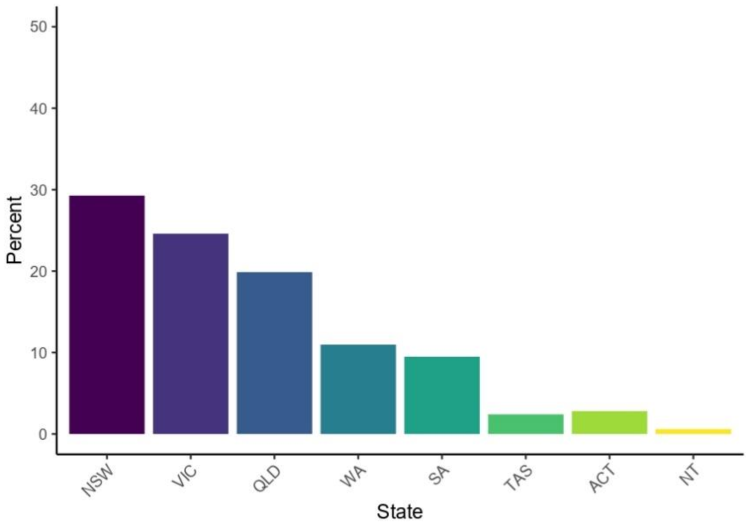

SM Figure 3: State of residence.

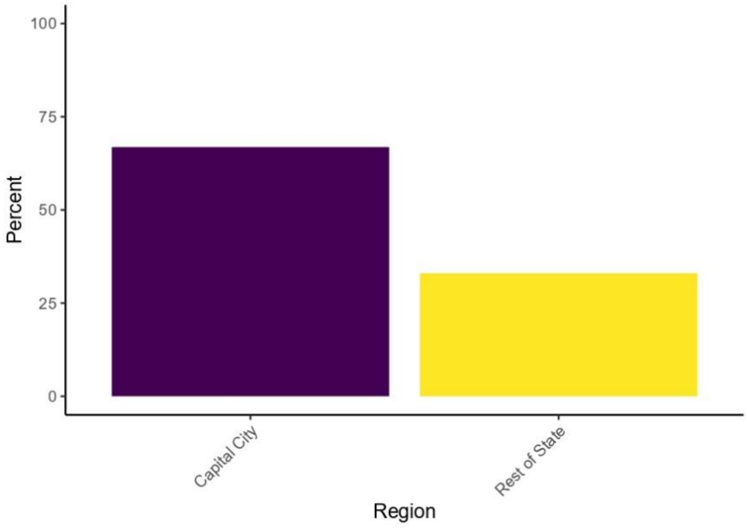

SM Figure 4: Regional status.

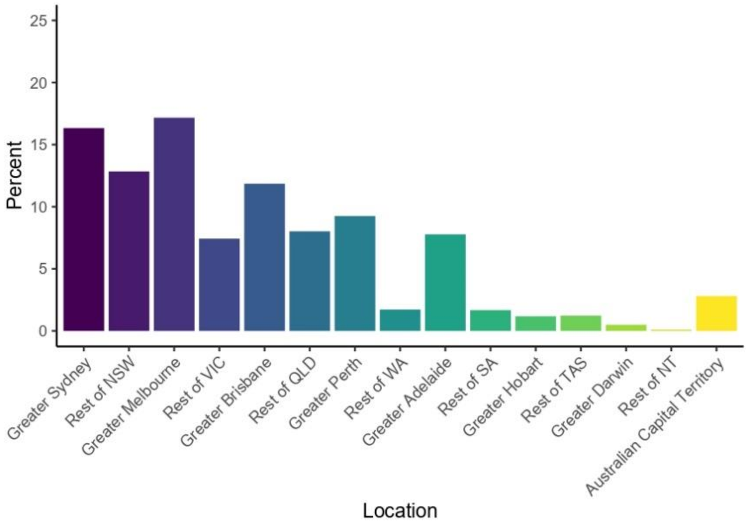

SM Figure 5: Geographical location.

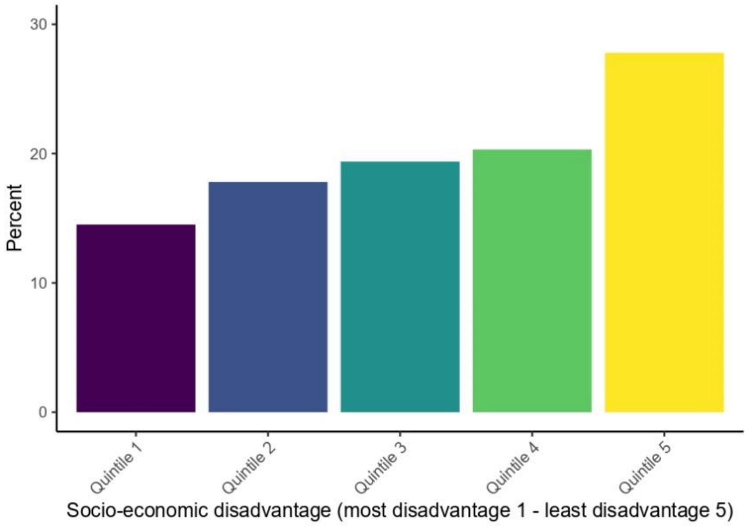

SM Figure 6: Residence in a neighbourhood categorised by presence of extent of local socio-economic disadvantage. I.e., not a measure of disadvantage for the respondent, but of the level of disadvantage of the area in which they reside.

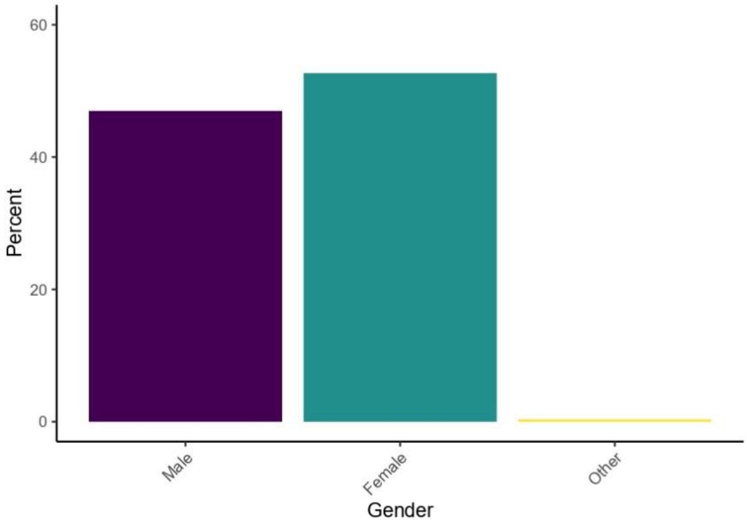

SM Figure 7: Gender.

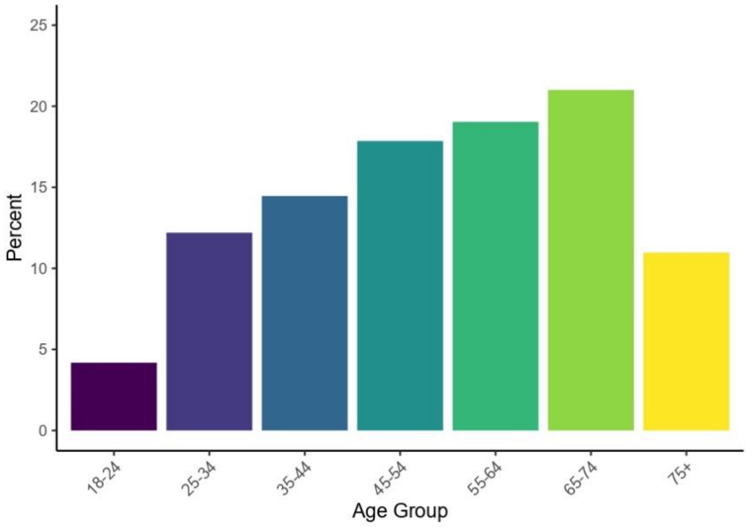

SM Figure 8: Age group distribution.

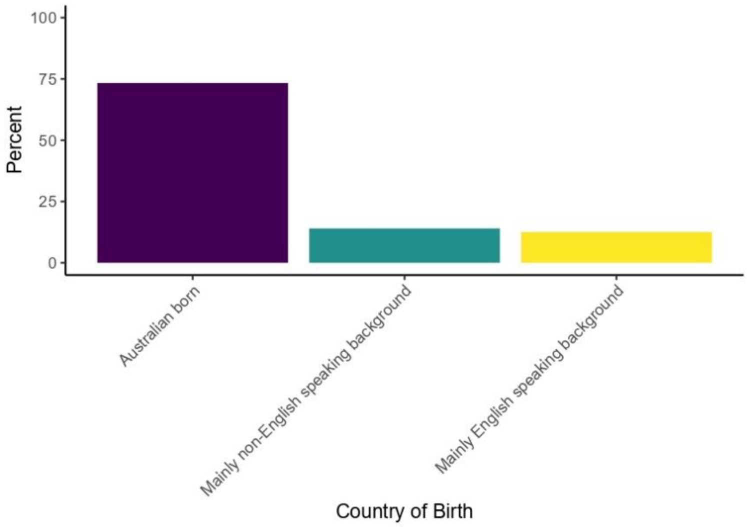

SM Figure 9: Country of birth.

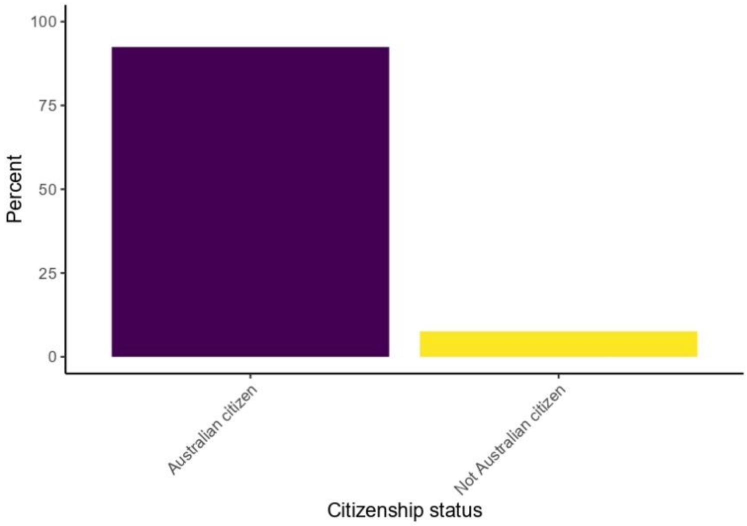

SM Figure 10: Australian citizenship status.

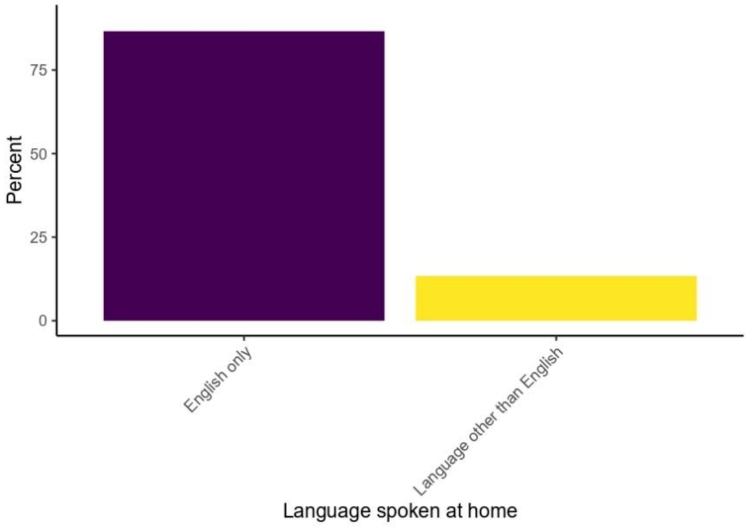

SM Figure 11: Main language spoken at home.

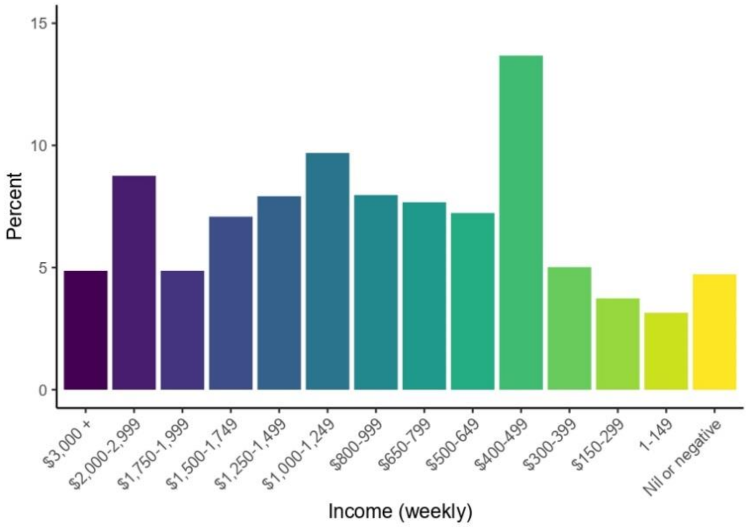

SM Figure 12: Weekly income.

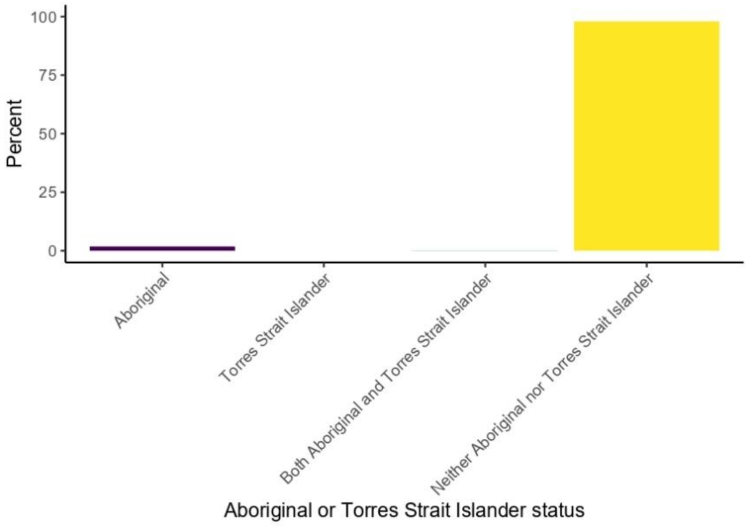

SM Figure 13: Aboriginal and/or Torres Strait Islander origin. *Note that due to the small number of respondents who recorded having Aboriginal or Torres Strait Islander origin (or both), we were not able to include this variable in subsequent analyses.*

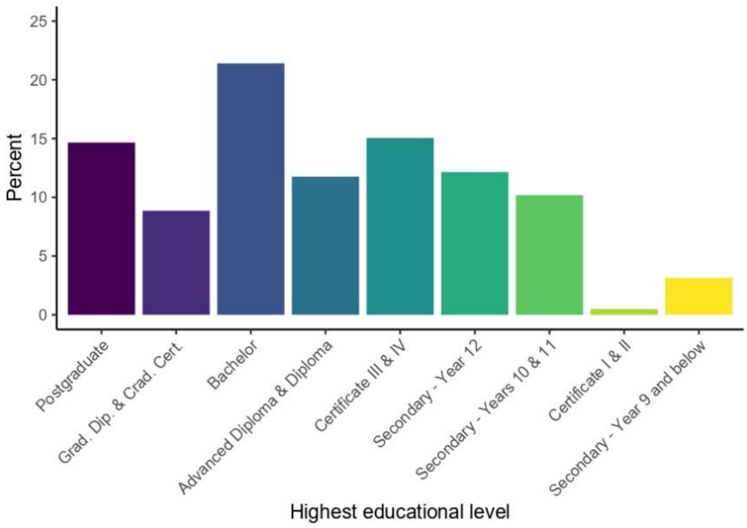

SM Figure 14: Level of highest educational attainment.

Descriptive summaries of key question responses

Q1. In your view, how important or unimportant is it that Australia takes action to reduce greenhouse gas emissions in order to help limit future climate change?

SM Table 3: Summary of Q1 responses.

	Extremely important	Important	Unimportant	Extremely unimportant
Count	1013	647	206	160
Percent	49.8	31.8	10.1	7.9
	Total extremely important & important		Total extremely unimportant & unimportant	
Count	1660		366	
Percent	81.7		18.1	

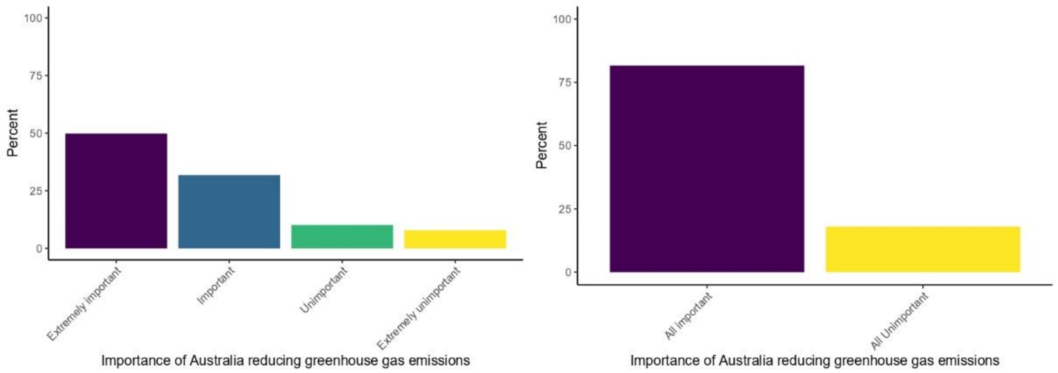

SM Figure 15: Summary of disaggregated and aggregated Q1 responses.

Q2. In your view, which energy source or mix of energy sources should provide Australia's electricity in 2050?

SM Table 4: Summary of Q2 responses.

	All renewables	Mostly renewables with some fossil fuels	Relatively even mix of fossil fuels & renewables	All fossil fuels (coal and gas)	All nuclear power
Count	713	601	444	43	212
Percent	35.1	29.6	21.8	2.1	10.4
	At minimum approx. half renewables				
Count	1758				
Percent	86.5				

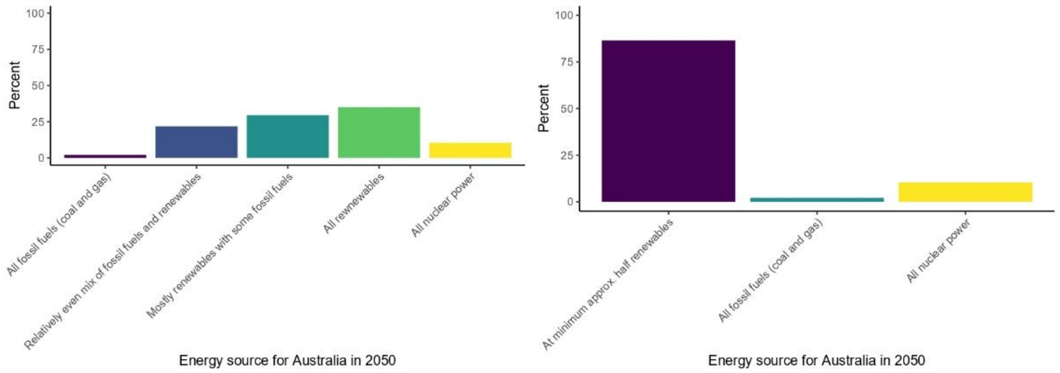

SM Figure 16: Summary of disaggregated and aggregated Q2 responses.

Q3. To what extent are you prepared to accept a personal cost in order to support action to reduce Australia's emissions?

SM Table 5: Summary of Q3 responses.

	Significant personal cost	Small personal cost	Not personally willing, but others should	Not personally willing, and others should not
Count	310	1159	114	434
Percent	15.2	57	5.6	21.3
	Total willing to accept a personal cost		Total unwilling to accept a personal cost	
Count	1469		548	
Percent	72.3		27	

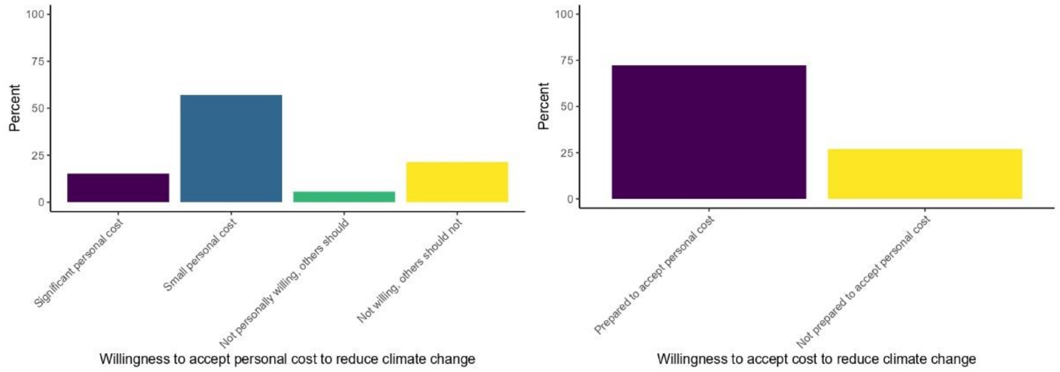

SM Figure 17: Summary of disaggregated and aggregated Q3 responses.

Q4. How much did the issue of climate change influence your vote in the 2019 Federal election? For you personally, would you say climate change was...?

SM Table 6: Summary of Q4 responses.

	The most important issue	One of the important issues	Not very important	Not at all important	Did not consider climate change when voting
Count	275	791	282	209	371
Percent	13.5	38.9	13.9	10.3	18.2
	Total important		Total not important		
Count	1066		862		
Percent	52.4		41.4		

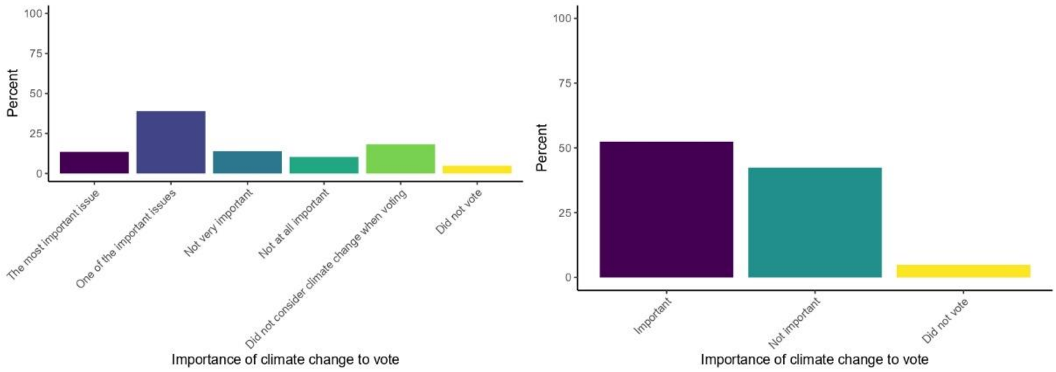

SM Figure 18: Summary of disaggregated and aggregated Q4 responses.

Ordinal logistic regression (Qs 1, 3, 4)

For the three ordinal response questions, we conducted first a series of univariate ordinal logistic regression (OLR) analyses, and then built a multivariate OLR model using all the variables that were significant in the univariate analyses. While univariate analyses can identify a range of influential variables that may be of interest, the multivariate analysis will examine interactions and covariance between the variables in order to determine those which are the most important social cleavages when viewed in their interactive context. Accordingly, the multivariate analyses offer a closer representation of the real social context that shapes attitudes toward climate change.

Q1. In your view, how important or unimportant is it that Australia takes action to reduce greenhouse gas emissions in order to help limit future climate change?

Our multivariate analysis identified the key factors driving differences in responses to Q1 (importance of GHG emissions reductions) when examined in the context of interactions with other drivers. Under multivariate analysis, the significant variables ($\alpha = 0.05$) predicting a difference in response to Q1 are:

- **Political preference at 2019 Federal election:** Greens & ALP voters were more likely than Lib, Nat, LNP voters to consider GHG emissions reductions to be important. Green voters were more likely than ALP voters to consider GHG reductions more important. Lib, Nat & LNP voters did not differ.
- **Gender:** women were more likely than men to consider GHG emissions reductions to be important.
- **Educational attainment:** people with higher educational attainment were more likely than those with lower educational attainment to consider GHG emissions reductions to be important.

In addition to the variables significant in the multivariate analysis, further variables were significant under univariate analysis only. In the univariate analysis, the following additional variables were significant in their influence on responses to Q1 (importance of GHG emissions reductions):

- **Age:** younger people were more likely than older people to consider GHG emissions reductions to be important.
- **Social disadvantage:** people living in areas with less social disadvantage were more likely than people living in areas with more social disadvantage to consider GHG emissions reductions to be important.
- **Political preference at 2019 Federal election (additional within-variables differences):** Lib voters were more likely than Nat & LNP voters to consider GHG emissions reductions to be important (however all were still less likely than Green and ALP voters, and Green voters were more likely than ALP voters).
- **State of residence:** Those who live in the ACT were more likely than those who live in every other state/territory to consider GHG emissions reductions to be important.
- **Country of birth:** Those born outside of Australia in an English speaking country were more likely than those born in Australia and those born outside of Australia in a non-English speaking country to consider GHG emissions reductions to be important.

Generational comparison:

PEW generational categories by years of birth

Generation	Start year	End year	Years in range	Mid-year of range
Silent	1928	1945	17	1936
Baby boomers	1946	1964	18	1955
Generation X	1965	1980	15	1972
Millennials (Generation Y)	1981	1996	15	1988
Generation Z	1997	2012	15	2004

Generation gaps (years average age difference):

	Silent	Baby boomers	Generation X	Millennials (Generation Y)	Generation Z
**Baby boomers**	19	-	-	-	-
**Generation X**	36	17	-	-	-
**Millennials (Generation Y)**	52	33	16	-	-
**Generation Z**	68	49	32	16	-

Generation gaps (OR of column generation compared to row generation):

Odds ratio based on univariate OR = 0.9866 taken to the power of the average age gap

	Silent	Baby boomers	Generation X	Millennials (Generation Y)
**Baby boomers**	0.77	-	-	-
**Generation X**	0.62	0.80	-	-
**Millennials (Generation Y)**	0.50	0.64	0.81	-
**Generation Z**	0.40	0.52	0.65	0.81

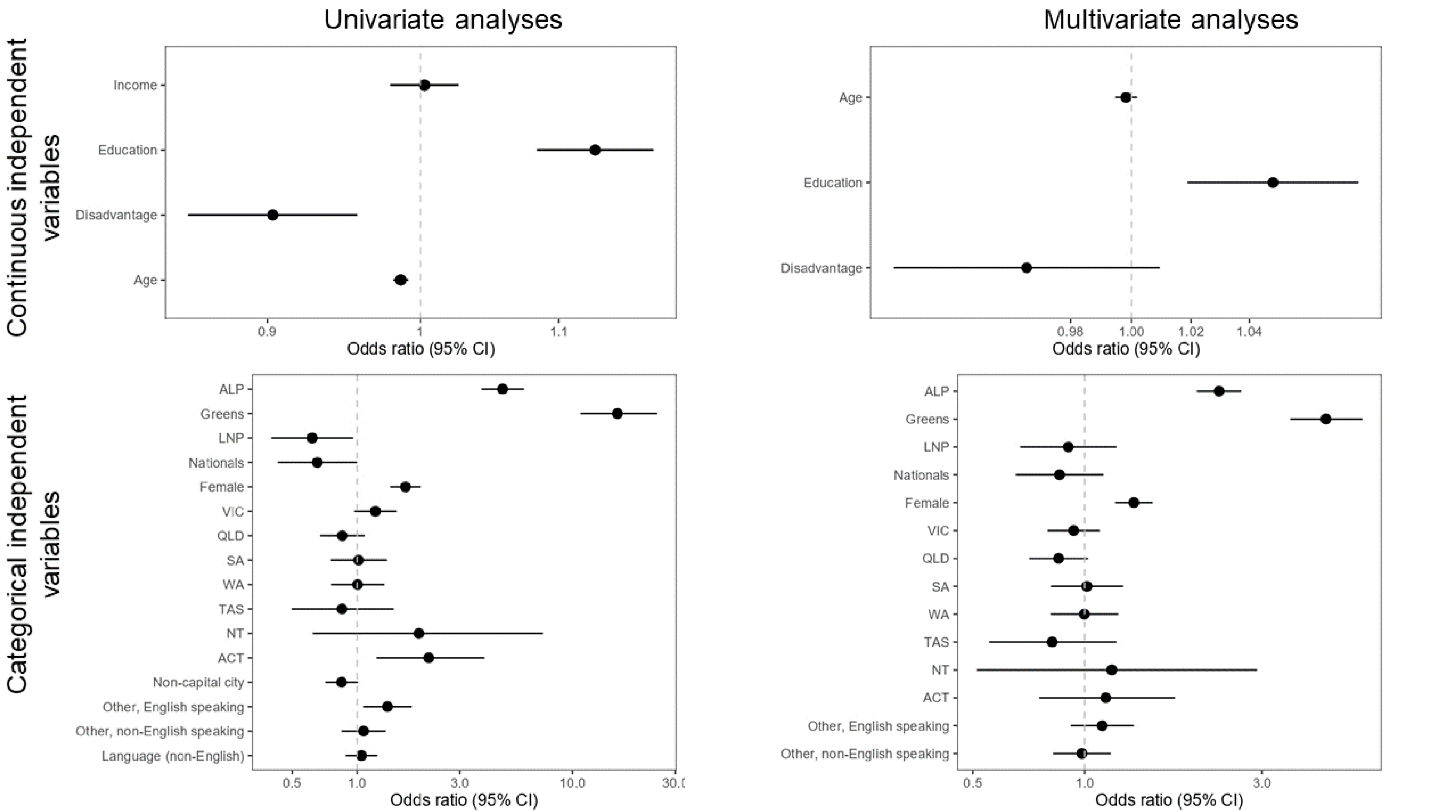

SM Figure 19: Q1 Forest plots illustrating odds ratios and 95% confidence interval of the odds ratio for Q1: "In your view, how important or unimportant is it that Australia takes action to reduce greenhouse gas emissions in order to help limit future climate change?" Left panels are univariate analyses; right panels are multivariate analyses; upper panels are continuous independent variables; lower panels are categorical independent variables. For continuous independent variables, the variables have been modified where necessary so that the OR is measuring more quantity of the variable listed on the y axis (i.e., more income, more education, more disadvantage in the area of residence, more age). For categorical independent variables, comparisons are to the default baseline category only (baseline category not displayed in plots: Liberal; Male; NSW; Capital city; English speaking background; Language (English)). Variables that are statistically significant at ($\alpha = 0.05$) do not cross the OR value of 1, indicated by dashed vertical line, with their 95% confidence interval.

SM Table 7: Q1 Results summary for univariate and multivariate ordinal logistic regression analyses. Results describe models with default baseline only.

		Univariate analyses			Multivariate analysis		
**Variable type**	**Variable**	**Odds Ratio (OR)**	**p-value**	**95% CI OR**	**Odds Ratio (OR)**	**p-value**	**95% CI OR**
**Continuous: results measure having 'more' of each variable against 'less'** (Disadvantage has been reversed from variable order to measure more disadvantage)							
	Age	0.9866	< **0.0001**	0.9817, 0.9915	0.9982	0.3387	0.9946, 1.0019
	Education	1.1279	< **0.0001**	1.0838, 1.1739	1.0482	**0.0012**	1.0188, 1.0784
	Income	0.9972	0.8164	1.0209, 0.9740			
	Disadvantage (reversed var.)	0.9033	**0.0006**	0.8523, 0.9574	0.9658	0.1229	0.9240, 1.0095
**Categorical: results compare a reference group against each other group**							
**Primary vote at 2019 Federal Election**							
Liberal Party	ALP	4.7365	< **0.0001**	3.7780, 5.9562	2.2997	< **0.0001**	2.0056, 2.6382
	Nationals	0.6540	**0.0492**	0.4287, 1.0000	0.8561	0.2626	0.6523, 1.1237
	Greens	16.1656	< **0.0001**	10.9010, 24.7199	4.4605	< **0.0001**	3.5780, 5.5941
	LNP (Qld)	0.6190	**0.0313**	0.4001, 0.9593	0.9038	0.5059	0.6710, 1.2176
**Gender identification**							
Male	Female	1.6785	< **0.0001**	1.4244, 1.9790	1.3565	< **0.0001**	1.2073, 1.5242
**State of Residence**							
NSW	VIC	1.2167	0.0920	0.9688, 1.5292	0.9341	0.4093	0.7944, 1.0983
	QLD	0.8547	0.1951	0.6740, 1.0841	0.8512	0.0840	0.7092, 1.0220
	SA	1.0161	0.9178	0.7515, 1.3774	1.0140	0.9033	0.8110, 1.2695
	WA	1.0054	0.9707	0.7557, 1.3403	0.9982	0.9865	0.8097, 1.2316
	TAS	0.8520	0.5630	0.4970, 1.4767	0.8181	0.3177	0.5533, 1.2166
	NT	1.9362	0.2787	0.6227, 7.2591	1.1831	0.7033	0.5119, 2.9128
	ACT	2.1506	**0.0090**	1.2310, 3.9071	1.1397	0.5419	0.7547, 1.7509
**Regional status**							
Capital city	Non-capital city	0.8483	0.0642	0.7128, 1.0100			
**Country of birth**							
Australia	Not-Australia, mainly English speaking background	1.3832	**0.0140**	1.0699, 1.7960	1.1151	0.2738	0.9182, 1.3566
	Not Australia, mainly non-English speaking background	1.0719	0.5653	0.8469, 1.3598	0.9830	0.8514	0.8222, 1.1764
**Language spoken at home**							
English	Language other than English	1.0481	0.5844	0.8864, 1.2418			

SM Table 8: Q1 Model replications for state of residence to determine multi-category comparative odds ratios and p-values.

**Model run →**	**Default**	**Rep 1**	**Rep 2**	**Rep 3**	**Rep 4**	**Rep 5**	**Rep 7**	**Rep 6**
**Baseline →**	**NSW**	**VIC**	**QLD**	**SA**	**WA**	**TAS**	**ACT**	**NT**
NSW (OR)		1.0706	1.1748	0.9862	1.0018	1.2223	0.8775	0.8452
p-value		0.4093	0.0840	0.9033	0.9864	0.3176	0.5420	0.7031
VIC (OR)	0.9341		1.0974	0.9212	0.9358	1.1418	0.8196	0.7895
p-value	0.4093		0.3339	0.4865	0.5434	0.5148	0.3524	0.5924
QLD (OR)	0.8512	0.9113		0.8395	0.8528	1.0405	0.7469	0.7194
p-value	0.0840	0.3339		0.1600	0.1774	0.8477	0.1827	0.4583
SA (OR)	1.0140	1.0855	1.1912		1.0158	1.2394	0.8897	0.8570
p-value	0.9033	0.4864	0.1600		0.9073	0.3196	0.6135	0.7312
WA (OR)	0.9982	1.0686	1.1727	0.9844		1.2201	0.8759	0.8436
p-value	0.9865	0.5434	0.1774	0.9073		0.3552	0.5559	0.7035
TAS (OR)	0.8181	0.8759	0.9612	0.8068	0.8197		0.7179	0.6915
p-value	0.3177	0.5151	0.8479	0.3197	0.3554		0.2462	0.4417
ACT (OR)	1.1397	1.2202	1.3389	1.1239	1.1417	1.3931		0.9632
p-value	0.5419	0.3522	0.1827	0.6134	0.5559	0.2461		0.9381
NT (OR)	1.1831	1.2667	1.3900	1.1668	1.1851	1.4460	1.0382	
p-value	0.7033	0.5925	0.4583	0.7313	0.7039	0.4418	0.9382	

SM Table 9: Q1 Model replications for country of birth to determine multi-category comparative odds ratios and p-values.

**Model run →**	**Default**	**Rep 8**	**Rep 9**
**Baseline →**	**Australian born**	**Other, mainly ESB**	**Other, mainly non-ESB**
Australian born (OR)		0.8968	1.0173
p-value		0.2739	0.8514
Other, mainly ESB (OR)	1.1151		1.1344
p-value	0.2738		0.3195
Other, mainly non-ESB (OR)	0.9830	0.8816	
p-value	0.8514	0.3195	

SM Table 10: Q1 Model replications for political party preference in the House of Representatives in the May 2019 Australian federal election to determine multi-category comparative odds ratios and p-values.

**Model run →**	**Default**	**Rep 10**	**Rep 11**	**Rep 12**	**Rep 13**
**Baseline →**	**Lib**	**ALP**	**Nats**	**Green**	**LNP**
Lib (OR)		0.4348	1.1682	0.2242	1.1064
p-value		< **0.0001**	0.2626	< **0.0001**	0.5059
ALP (OR)	2.2997		2.6864	0.5156	2.5444
p-value	< **0.0001**		< **0.0001**	< **0.0001**	< **0.0001**
Nats (OR)	0.8561	0.3722		0.1919	0.9472
p-value	0.2626	< **0.0001**		< **0.0001**	0.7814
Green (OR)	4.4605	1.9396	5.2105		4.9352
p-value	< **0.0001**	< **0.0001**	< **0.0001**		< **0.0001**
LNP (OR)	0.9038	0.3930	1.0558	0.2026	
p-value	0.5059	< **0.0001**	0.7814	< **0.0001**	

Q3. To what extent are you prepared to accept a personal cost in order to support action to reduce Australia's emissions?

Our multivariate analysis identified the key factors driving differences in responses to Q3 (willingness to accept a personal cost) when examined in the context of interactions with other drivers. Under multivariate analysis, the significant variables ($\alpha = 0.05$) predicting a difference in response to Q3 are:

- **Political preference at 2019 Federal election:** Greens & ALP voters were more likely than Lib, Nat & LNP voters to be willing to accept a personal cost. Green voters were more likely than ALP voters to be willing to accept a personal cost. Lib, Nat & LNP voters did not differ.
- **Educational attainment:** People with higher education were more likely than those with low education to be willing to accept a personal cost.
- **State of residence:** Residents of QLD were less likely than residents of NSW to be willing to accept a personal cost. There were no differences between all other states/territories (including with both QLD and NSW).
- **Social disadvantage:** people living in areas with less social disadvantage were more willing than people living in areas with more disadvantage to accept a personal cost.

In addition to the variables significant in the multivariate analysis, further variables were significant under univariate analysis only. In the univariate analysis, the following additional variables were significant in their influence on responses to Q3 (willingness to accept a personal cost):

- **Age:** Younger people were more willing than older people to accept a personal cost.
- **State of residence (additional within-variable differences):** Residents of QLD were less likely than residents of NSW, the NT and the ACT to be willing to accept a personal cost. Residents of the ACT were more likely than residents of all states/territories other than the NT to be willing to accept a personal cost. QLD, VIC, SA, WA, TAS did not differ significantly. Additionally, NSW, VIC, SA, WA, TAS, and the NT did not differ significantly. While the NT had the largest OR, it also had the largest 95% confidence interval, indicating high variability in the small number of NT responses.
- **Income:** People with greater income were more likely than people with less income to be willing to accept a personal cost.
- **Country of birth:** People born outside of Australia in a predominantly English-speaking country are more likely than people born in Australia and outside of Australia in a predominantly non-English speaking country to be willing to accept a personal cost.

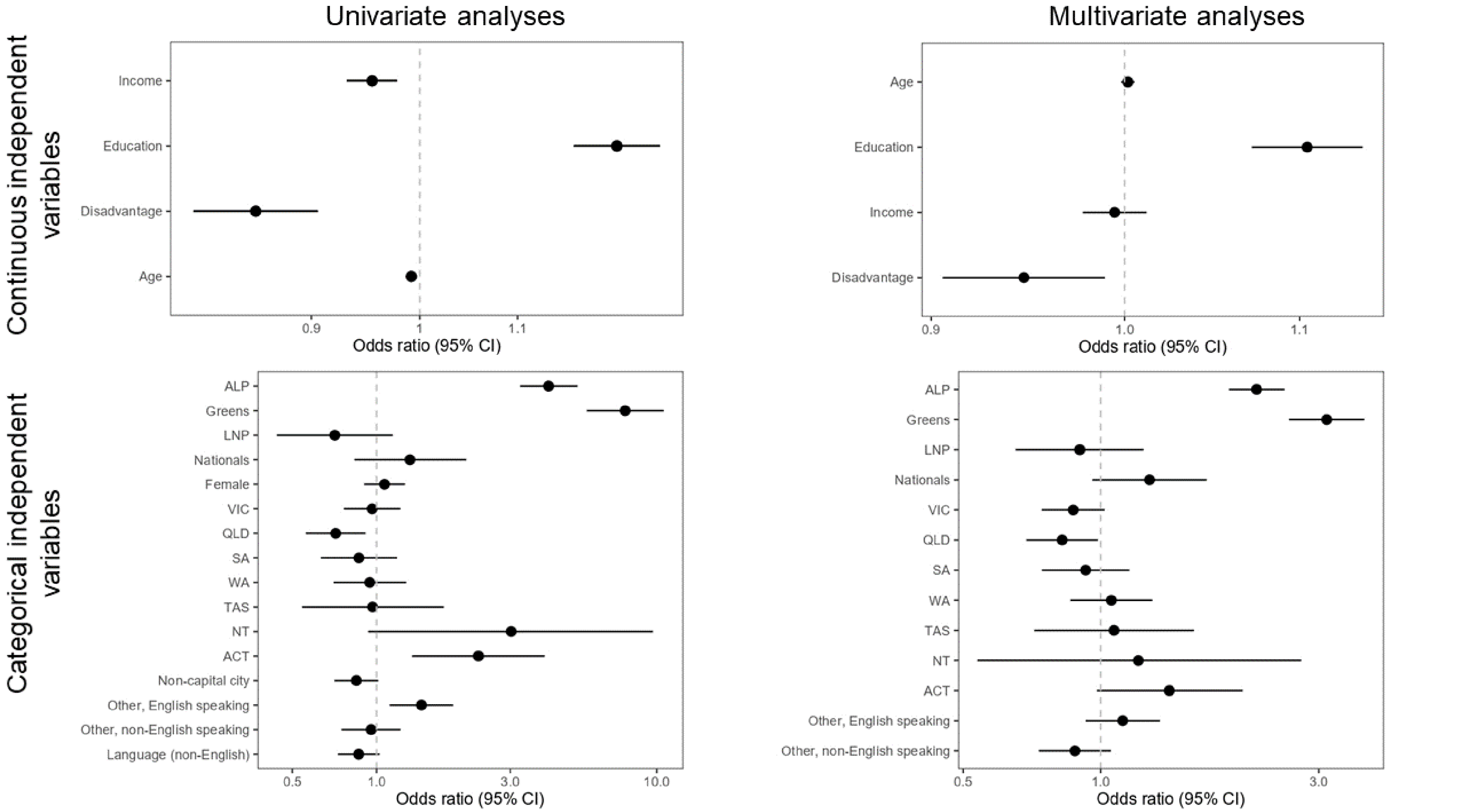

SM Figure 20: Q3 Forest plots illustrating odds ratios and 95% confidence interval of the odds ratio for Q3: "To what extent are you prepared to accept a personal cost in order to support action to reduce Australia's emissions?" Left panels are univariate analyses; right panels are multivariate analyses; upper panels are continuous independent variables; lower panels are categorical independent variables. For continuous independent variables, the variables have been modified where necessary so that the OR is measuring more quantity of the variable listed on the y axis (i.e., more income, more education, more disadvantage in the area of residence, more age). For categorical independent variables, comparisons are to the default baseline category only (baseline category not displayed in plots: Liberal; Male; NSW; Capital city; English speaking background; Language (English)). Variables that are statistically significant at ($\alpha = 0.05$) do not cross the OR value of 1, indicated by dashed vertical line, with their 95% confidence interval.

SM Table 11: Q3 results summary for univariate and multivariate ordinal logistic regression analyses. Results describe models with default baseline only.

Variable type	Variable	Univariate analyses			Multivariate analysis		
		Odds Ratio (OR)	p-value	95% CI OR	Odds Ratio (OR)	p-value	95% CI OR
**Continuous: results measure having 'more' of each variable against 'less'** (Disadvantage has been reversed from variable order to measure more disadvantage)							
	Age	0.9919	**0.0015**	0.9869, 0.9969	1.0017	0.3699	0.9980, 1.0053
	Education	1.2117	**< 0.0001**	1.1623, 1.2635	1.1045	**< 0.0001**	1.0717, 1.1384
	Income	1.0473	**0.0002**	1.0732, 1.0220	0.9945	0.5331	0.9774, 1.0119
	Disadvantage (reversed var.)	0.8525	**< 0.0001**	0.8024, 0.9055	0.9466	**0.0148**	0.9056, 0.9893
**Categorical: results compare a reference group against each other group**							
**Primary vote at 2019 Federal Election**							
Liberal Party	ALP	4.1124	**< 0.0001**	3.2508, 5.2212	2.1937	**< 0.0001**	1.9081, 2.5238
	Nationals	1.3154	0.2420	0.8335, 2.0908	1.2784	0.0949	0.9582, 1.7052
	Greens	7.7158	**< 0.0001**	5.6334,10.6061	3.1224	**< 0.0001**	2.5837, 3.7778
	LNP (Qld)	0.7093	0.1564	0.4405, 1.1419	0.8995	0.5199	0.6509, 1.2407
**Gender identification**							
Male	Female	1.0673	0.4466	0.9029, 1.2630			
**State of Residence**							
NSW	VIC	0.9639	0.7575	0.7632, 1.2174	0.8703	0.0863	0.7425, 1.0200
	QLD	0.7143	**0.0073**	0.5585, 0.9133	0.8230	**0.0338**	0.6875, 0.9851
	SA	0.8648	0.3650	0.6320, 1.1848	0.9266	0.4983	0.7432, 1.1552
	WA	0.9455	0.7125	0.7019, 1.2746	1.0547	0.6148	0.8571, 1.2980
	TAS	0.9672	0.9106	0.5420, 1.7367	1.0686	0.7467	0.7145, 1.5990
	NT	3.0229	0.0612	0.9324, 9.7023	1.2081	0.6495	0.5371, 2.7484
	ACT	2.3104	**0.0026**	1.3400, 3.9808	1.4131	0.0652	0.9800, 2.0446
**Regional status**							
Capital city	Non-capital city	0.8473	0.0708	0.7078, 1.0140			
**Country of birth**							
Australia	Not-Australia, mainly English speaking background	1.4465	**0.0056**	1.1133, 1.8770	1.1170	0.2472	0.9262, 1.3473
	Not Australia, mainly non-English speaking background	0.9564	0.7200	0.7487, 1.2191	0.8777	0.1582	0.7321, 1.0520
**Language spoken at home**							
English	Language other than English	0.8634	0.0922	0.7280, 1.0246			

SM Table 12: Q3 Model replications for state of residence to determine multi-category comparative odds ratios and p-values.

**Model run →**	**Default**	**Rep 1**	**Rep 2**	**Rep 3**	**Rep 4**	**Rep 5**	**Rep 7**	**Rep 6**
**Baseline →**	**NSW**	**VIC**	**QLD**	**SA**	**WA**	**TAS**	**ACT**	**NT**
NSW (OR)		1.1490	1.2150	1.0792	0.9575	0.9332	0.7118	0.8136
p-value		0.0863	**0.0338**	0.4983	0.6768	0.7329	0.0690	0.6192
VIC (OR)	0.8703		1.0575	0.9392	0.8234	0.8025	0.6121	0.6996
p-value	0.0863		0.5546	0.5881	0.0672	0.2829	**0.0085**	0.3898
QLD (OR)	0.8230	0.9457		0.8882	0.7885	0.7685	0.5862	0.6700
p-value	**0.0338**	0.5546		0.3332	**0.0394**	0.2063	**0.0055**	0.3378
SA (OR)	0.9266	1.0647	1.1259		0.8768	0.8546	0.6519	0.7450
p-value	0.4983	0.5881	0.3332		0.3164	0.4685	**0.0369**	0.4866
WA (OR)	1.0547	1.2119	1.2815	1.1382		0.9746	0.7434	0.8497
p-value	0.6148	0.0754	**0.0336**	0.3317		0.9053	0.1366	0.6985
TAS (OR)	1.0686	1.2279	1.2984	1.1532	1.0260		0.7628	0.8718
p-value	0.7467	0.3234	0.2162	0.5166	0.9053		0.3113	0.7638
ACT (OR)	1.2081	1.6237	1.7171	1.5250	1.3451	1.3110		
p-value	0.6495	**0.0096**	**0.0051**	**0.0407**	0.1366	0.3113		
NT (OR)	1.4131	1.3881	1.4679	1.3037	1.1769	1.1470	0.8750	1.1429
p-value	0.0652	0.4310	0.3594	0.5321	0.6985	0.7638	0.7651	0.7651

SM Table 13: Q3 Model replications for country of birth to determine multi-category comparative odds ratios and p-values.

**Model run →**	**Default**	**Rep 8**	**Rep 9**
**Baseline →**	**Australian born**	**Other, mainly ESB**	**Other, mainly non-ESB**
Australian born (OR)		0.8953	1.1394
p-value		0.2472	0.1582
Other, mainly ESB (OR)	1.1170		1.2727
p-value	0.2472		0.0537
Other, mainly non-ESB (OR)	0.8777	0.7858	
p-value	0.1582	0.0537	

SM Table 14: Q3 Model replications for political party preference in the House of Representatives in the May 2019 Australian federal election to determine multi-category comparative odds ratios and p-values.

**Model run →**	**Default**	**Rep 10**	**Rep 11**	**Rep 12**	**Rep 13**
**Baseline →**	**Lib**	**ALP**	**Nats**	**Green**	**LNP**
Lib (OR)		0.4558	0.7822	0.3203	1.1118
p-value		**< 0.0001**	0.0949	**< 0.0001**	0.5199
ALP (OR)	2.1937		1.7160	0.7026	2.4389
p-value	**< 0.0001**		**0.0003**	**0.0001**	**< 0.0001**
Nats (OR)	1.2784	0.5827		0.4094	1.4213
p-value	0.0949	**0.0003**		**< 0.0001**	0.0946
Green (OR)	3.1224	1.4234	2.4425		3.4715
p-value	**< 0.0001**	**0.0001**	**< 0.0001**		**< 0.0001**
LNP (OR)	0.8995	0.4100	0.7036	0.2881	
p-value	0.5199	**< 0.0001**	0.0946	**< 0.0001**	

Q4. How much did the issue of climate change influence your vote in the 2019 Federal election? For you personally, would you say climate change was...?

Our multivariate analysis identified the key factors driving differences in responses to Q4 (importance of climate change to voting decision) when examined in the context of interactions with other drivers. Under multivariate analysis, the significant variables ($\alpha = 0.05$) predicting a difference in response to Q4 are:

- **Political preference at 2019 Federal election:** Greens & ALP voters were more likely than Lib, Nat & LNP voters to consider climate change in their voting decision. Green voters were more likely than ALP voters. Lib, Nat & LNP voters did not differ.
- **Educational attainment:** People with higher education were more likely than those with low education to consider climate change in their voting decision.
- **State of residence:** Residents of QLD were less likely than residents of NSW to consider climate change in their voting decision. There were no differences between all other states/territories (including with both QLD and NSW).
- **Social disadvantage:** people living in areas with less disadvantage were more willing than people living in areas with more disadvantage to accept a personal cost.

In addition to the variables significant in the multivariate analysis, further variables were significant under univariate analysis only. In the univariate analysis, the following additional variables were significant in their influence on responses to Q4 (importance of climate change to voting decision):

- **Age:** Younger people were more likely than older people to consider climate change in their voting decision.
- **Regional status:** People living in capital cities were more likely that people living outside of capital cities to consider climate change in their voting decision.

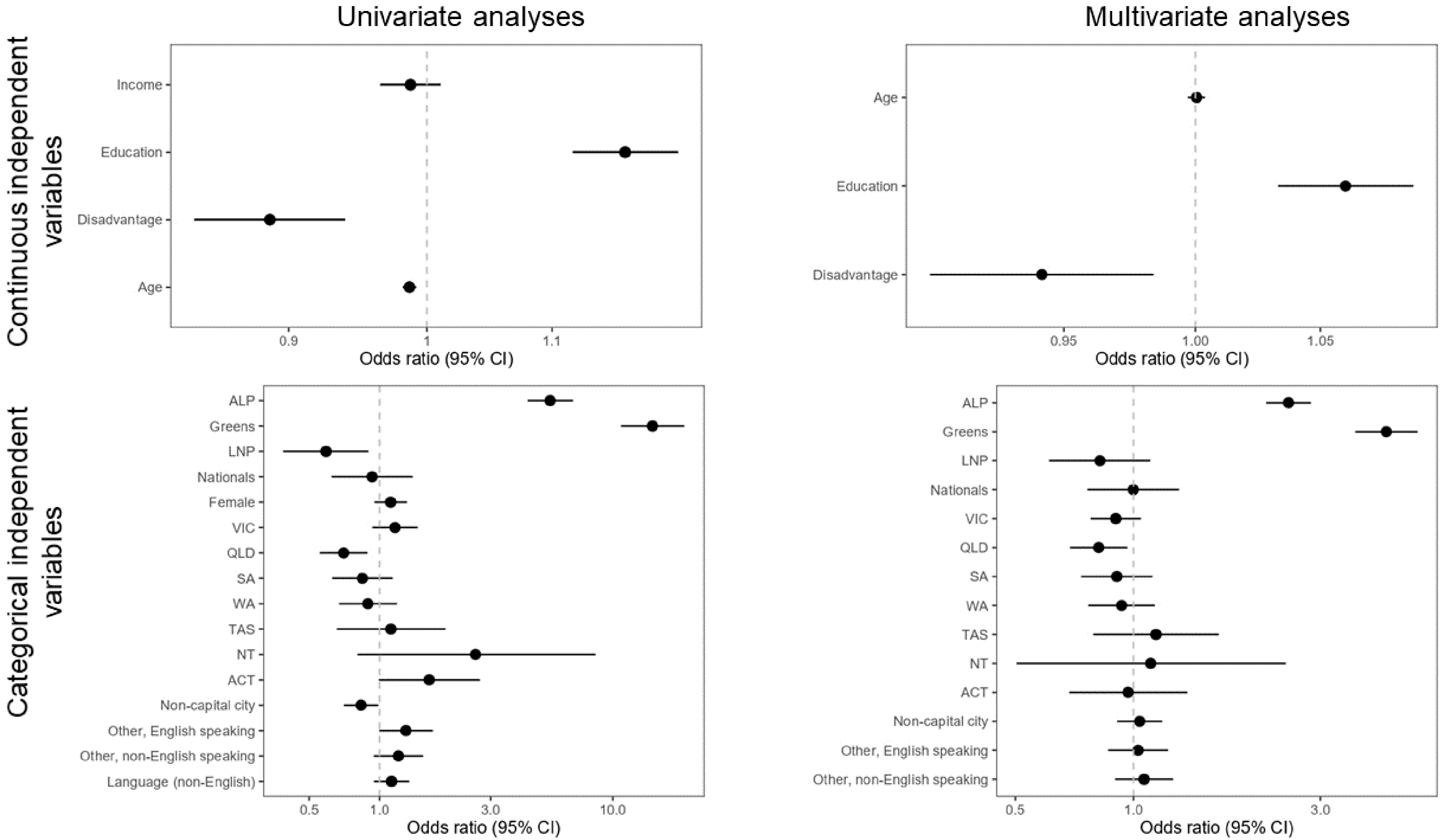

SM Figure 21: Q4 Forest plots illustrating odds ratios and 95% confidence interval of the odds ratio for Q4: "How much did the issue of climate change influence your vote in the 2019 Federal election? For you personally, would you say climate change was...?" Left panels are univariate analyses; right panels are multivariate analyses; upper panels are continuous independent variables; lower panels are categorical independent variables. For continuous independent variables, the variables have been modified where necessary so that the OR is measuring more quantity of the variable listed on the y axis (i.e., more income, more education, more disadvantage in the area of residence, more age). For categorical independent variables, comparisons are to the default baseline category only (baseline category not displayed in plots: Liberal; Male; NSW; Capital city; English speaking background; Language (English)). Variables that are statistically significant at ($\alpha = 0.05$) do not cross the OR value of 1, indicated by dashed vertical line, with their 95% confidence interval.

SM Table 15: Q4 Results summary for univariate and multivariate ordinal logistic regression analyses. Results describe models with default baseline only.

		Univariate analyses			Multivariate analysis		
**Variable type**	**Variable**	**Odds Ratio (OR)**	**p-value**	**95% CI OR**	**Odds Ratio (OR)**	**p-value**	**95% CI OR**
**Continuous: results measure having 'more' of each variable against 'less'** (Disadvantage has been reversed from variable order to measure more disadvantage)							
	Age	0.9868	**< 0.0001**	0.9819, 0.9916	1.0005	0.7893	0.9971, 1.0038
	Education	1.1629	**< 0.0001**	1.1174, 1.2106	1.0603	**< 0.0001**	1.0327, 1.0888
	Income	1.0127	0.2805	1.0362, 0.9897			
	Disadvantage (reversed var.)	0.8872	**< 0.0001**	0.8378, 0.9394	0.9419	**0.0072**	0.9017, 0.9839
**Categorical: results compare a reference group against each other group**							
**Primary vote at 2019 Federal Election**							
Liberal Party	ALP	5.3900	**< 0.0001**	4.3122, 6.7574	2.4877	**< 0.0001**	2.1828, 2.8364
	Nationals	0.9294	0.7207	0.6219, 1.3896	1.0000	0.9999	0.7640, 1.3083
	Greens	14.8001	**< 0.0001**	10.854, 20.272	4.4142	**< 0.0001**	3.6765, 5.3053
	LNP (Qld)	0.5900	**0.0135**	0.3869, 0.8956	0.8216	0.1950	0.6100, 1.1054
**Gender identification**							
Male	Female	1.1169	0.1752	0.9521, 1.3108			
**State of Residence**							
NSW	VIC	1.1661	0.1763	0.9334, 1.4573	0.9021	0.1719	0.7781, 1.0458
	QLD	0.7022	**0.0031**	0.5554, 0.8876	0.8157	**0.0184**	0.6886, 0.9661
	SA	0.8443	0.2667	0.6263, 1.1386	0.3641	0.9074	0.7356, 1.1192
	WA	0.8919	0.4327	0.6704, 1.1872	0.9337	0.4909	0.7682, 1.1349
	TAS	1.1188	0.6806	0.6568, 1.9172	1.1419	0.4805	0.7901, 1.6519
	NT	2.5787	0.1120	0.8031, 8.4402	1.1067	0.8013	0.5035, 2.4473
	ACT	1.6334	0.0538	0.9935, 2.6961	0.9696	0.8616	0.6859, 1.3724
**Regional status**							
Capital city	Non-capital city	0.8332	**0.0367**	0.7021, 0.9887	1.0374	0.5850	0.9093, 1.1837
**Country of birth**							
Australia	Not-Australia, mainly English speaking background	1.2981	0.0532	0.9969, 1.6921	1.0281	0.7581	0.8619, 1.2264
	Not Australia, mainly non-English speaking background	1.2053	0.1303	0.9467, 1.5359	1.0648	0.4686	0.8985, 1.2619
**Language spoken at home**							
English	Language other than English	1.1259	0.1801	0.9470, 1.3396			

SM Table 16: Q4 Model replications for state of residence to determine multi-category comparative odds ratios and p-values.

**Model run →**	**Default**	**Rep 1**	**Rep 2**	**Rep 3**	**Rep 4**	**Rep 5**	**Rep 7**	**Rep 6**
**Baseline →**	**NSW**	**VIC**	**QLD**	**SA**	**WA**	**TAS**	**ACT**	**NT**
NSW (OR)		1.1085	1.2259	1.102	1.071	0.8757	1.0314	0.9037
p-value		0.1719	**0.0184**	0.3641	0.4909	0.4805	0.8613	0.8013
VIC (OR)	0.9021		1.1059	0.9941	0.9661	0.7900	0.9304	0.8152
p-value	0.1719		0.2564	0.9570	0.7321	0.2153	0.6820	0.6118
QLD (OR)	0.8157	0.9042		0.8989	0.8736	0.7143	0.8413	0.7371
p-value	**0.0184**	0.2564		0.3605	0.2190	0.0825	0.3426	0.4515
SA (OR)	0.3641	1.0059	1.1124		0.9718	0.7947	0.9359	0.82
p-value	0.9074	0.957	0.3605		0.8187	0.2561	0.7324	0.6285
WA (OR)	0.9337	1.0351	1.1447	1.029		0.8177	0.9630	0.8438
p-value	0.4909	0.7321	0.2190	0.8187		0.3173	0.8411	0.6769
TAS (OR)	1.1419	1.2658	1.3999	1.2584	1.2230		1.1778	1.0319
p-value	0.4805	0.2152	0.0825	0.2561	0.3173		0.5126	0.943
ACT (OR)	0.9696	1.0749	1.1887	1.0686	1.0384	0.8491		0.8762
p-value	0.8013	0.6817	0.3423	0.7320	0.8409	0.5128		0.7596
NT (OR)	1.1067	1.2267	1.3566	1.2195	1.1852	0.9691	1.1415	
p-value	0.8616	0.6118	0.4515	0.6285	0.6769	0.9430	0.7593	

SM Table 17: Q4 Model replications for country of birth to determine multi-category comparative odds ratios and p-values.

**Model run →**	**Default**	**Rep 8**	**Rep 9**
**Baseline →**	**Australian born**	**Other, mainly ESB**	**Other, mainly non-ESB**
Australian born (OR)		0.9727	
p-value		0.7581	
Other, mainly ESB (OR)	1.0281		0.9391
p-value	0.7581		0.4687
Other, mainly non-ESB (OR)	1.0648	1.0357	0.9655
p-value	0.4686	0.7638	0.7638

SM Table 18: Q4 Model replications for political party preference in the House of Representatives in the May 2019 Australian federal election to determine multi-category comparative odds ratios and p-values.

**Model run →**	**Default**	**Rep 10**	**Rep 11**	**Rep 12**	**Rep 13**
**Baseline →**	**Lib**	**ALP**	**Nats**	**Green**	**LNP**
Lib (OR)		0.4020	1.0000	0.2265	1.2172
p-value		< **0.0001**	0.9999	< **0.0001**	0.1950
ALP (OR)	2.4877		2.4877	0.5636	3.0280
p-value	< **0.0001**		< **0.0001**	< **0.0001**	< **0.0001**
Nats (OR)	1.0000	0.4020		0.2265	1.2172
p-value	0.9999	< **0.0001**		< **0.0001**	0.3120
Green (OR)	4.4142	1.7744	4.4141		5.3729
p-value	< **0.0001**	< **0.0001**	< **0.0001**		< **0.0001**
LNP (OR)	0.8216	0.3303	0.8216	0.1861	
p-value	0.1950	< **0.0001**	0.3120	< **0.0001**	

ANOVA and chi-squared analysis (Q 2)

Q2. In your view, which energy source or mix of energy sources should provide Australia's electricity in 2050?

We found a number of differences in preferences for Australia's future energy mix based on a series of univariate analyses, both ANOVA and Pearson's chi-squared test of independence. All variables except for country of birth and language spoken at home were significantly associated with energy mix preference (to varying extents). Due to the different data types, we cannot compare statistically between each of the variables, so we cannot identify potential covariance and interactions. Nevertheless, we see a strong signal of influence on preferred energy mix from politics, gender, age & education.

- **Support for renewable energy:** Women, younger people, people living in cities, and more educated people show the strongest positive association with support for renewable energy.
- **Support for nuclear energy:** Men, Liberal voters, and less educated people show the strongest positive association with support for nuclear energy.
- **Support for fossil fuels:** Men, people from NSW and QLD, LNP and Liberal voters show the strongest positive association with support for fossil fuels.

Across the independent variables we can note more nuanced findings:

- **Gender:** Men are substantially more likely than women to support fossil fuels and nuclear energy. Women are more likely than men to support renewable energy.
- **Political preference at 2019 Federal election:** Green voters substantially more likely than all other voters to support all renewables. ALP voters still high on all renewables compared to coalition, with Liberal voters strongly opposed to all renewables. Green voters are negatively associated with all options other than all renewables. All fossil fuels more likely to be supported by LNP voters (QLD) and to lesser extent by Liberal voters. Support for all nuclear strongly associated with Liberal voters, strongly opposed by ALP and Green voters.
- **Educational attainment:** Higher education is associated with support for mostly renewables and some fossil fuels and all renewables compared to lower educational attainment support for an equal mix of renewables and fossil fuels. Lower educational attainment is associated with more support for all nuclear power compared to mostly renewables and some fossil fuels and all renewables.
- **Age:** Support for all renewables is associated with younger people compared to older people across all comparisons. Younger people also more likely to support mostly renewables and some fossil fuels compared to equal fossil fuels and renewables.
- **State of residence:** All renewables was most supported in NT, ACT, and VIC, and least supported in SA and QLD. SA was more likely than all others to support nuclear power. NSW and QLD were more likely than all others to support all fossil fuels. WA presented the least support for all fossil fuels.
- **Regional status:** All renewables is more likely to be supported in cities than outside of cities. Residents outside of capital cities are more likely than residents in capital cities to support a mix of renewables and fossil fuels (both even mix and most renewables mix). There was no difference between residents in cities and residents outside of cities with regard to levels of support for all fossil fuels and all nuclear power.
- **Social disadvantage:** People living in areas with less social disadvantage were more likely than people living in areas with more social disadvantage to support all renewables ahead of a relatively equal mix of renewables and fossil fuels.
- **Income**: Support for a relatively equal mix of fossil fuels and renewables associated with lower income compared to higher income support for mostly renewables, some fossil fuels, all renewables, and all nuclear.

SM Table 19: Summary of ANOVA and Pearson's chi-squared tests and post-hoc tests on preferences for Australia's future energy mix.

Variable	Data type	Test	Result (p-value)	Post-hoc approach	Result
Gender	Categorical	Chi-square	< 0.001	View contingency table	• Men substantially more likely than women to support all nuclear power (contribution to chi-square test statistic: 6.0 men; -5.7 women).<br>• Men more likely than women to support all fossil fuels (cont. to CS: men 1.6; women -1.5).<br>• Women more likely than men to support mostly renewables (women 2.1; men -2.2) and all renewables (women 1.4; men -1.5).
State	Categorical	Chi-square	0.01681	View contingency table	• South Australia more likely than all others to support all nuclear power (SA 2.9; all others max |1.2|).<br>• NSW and QLD more likely than all others to support all fossil fuels (NSW 1.2; QLD 1.2), WA least support (-1.3).<br>• All renewables most supported in NT (1.8), ACT (1.7), VIC (1.6). Least support in SA (-1.7) and QLD (-1.2).
Region (capital city v. not)	Categorical	Chi-square	0.006606	View contingency table	• All renewables more likely to be supported in cities than in the rest of the state (cities 1.7; rest of state -2.4).<br>• No difference between cities and rest of state re all fossil fuels and all nuclear power.<br>• Rest of state v. cities more likely to support a mix of renewables and fossil fuels: mostly renewables and some FF (rest of state 1.4; cities -1.0) & even renewables and fossil fuels (rest of state 1.4; cities -1.0).
Country of birth	Categorical	Chi-square	0.09457	NA	
Language spoken at home	Categorical	Chi-square	0.6914	NA	
House of reps first pref	Categorical	Chi-square	< 0.001	View contingency table	• Green voters substantially more likely to support all renewables than all other voters, ALP voters still high compared to coalition, Liberal voters strongly opposed (Green 7.9; ALP 4.4; Lib -7.4; LNP -2.6, Nat -2.6).<br>• Green voters are negatively associated with all options other than all renewables.<br>• All fossil fuels more likely to be supported by LNP voters (QLD) and to lesser extent by Liberal voters (LNP 4.1, Lib 1.7, Nat 0.2, ALP -1.9, Green -2.0).

					• All nuclear strongly associated with Liberal voters, strongly opposed by ALP and Green voters (Lib 4.0; Green -3.1; ALP -3.0; LNP 2.1; Nat 0.6).<br>• Liberal voters and Nationals voters strongly associated with a preference for a fairly equal mix of fossil fuels and renewable sources (Lib 6.3; Nat 3.4; LNP 2.5; ALP -4.6; Green -6.0).
SEIFA	Continuous	ANOVA	0.002812	Tukey's post-hoc	• Only significant difference is between all renewables and equal mix of renewables and fossil fuels (p = 0.0038), with the latter having a higher SEIFA mean, indicating less disadvantage is associated with an attitude supporting an equal mix of renewables and fossil fuels compared to support for all renewables.<br>• No other post-ANOVA associations.
Income	Continuous	ANOVA	0.00151	Tukey's post-hoc	• Equal FF and renewables associated with lower income compared to mostly renewables, some FF (p = 0.017), all renewables (p = 0.019), and all nuclear (p = 0.003).<br>• No other post-ANOVA associations.
Education	Continuous	ANOVA	< 0.001	Tukey's post-hoc	• Higher education is associated with support for mostly renewables and some FF (p < 0.001) and all renewables (p <0.001) compared to an equal mix of renewables and FF.<br>• Lower education is associated with support for all nuclear power compared to mostly renewables and some FF (p = 0.022) and all renewables (p < 0.001).<br>• No other post-ANOVA associations.
Age	Continuous	ANOVA	< 0.001	Tukey's post-hoc	• Support for all renewables associated with younger people across all comparisons: all fossil fuels (p = 0.007); equal FF and renewables (p < 0.001); mostly renewables, some FF (p < 0.001); all nuclear (p < 0.001).<br>• Younger people also more likely to support mostly renewables and some FF compared to equal FF and renewables (p < 0.001).

Age projections

We projected future aggregate Australian public opinion on climate change using the responses to Q1 and official ABS population projections.

First, we split our data into 5-year age brackets, and then for each age bracket recorded the percent of respondents in that age bracket that answered Q1 with ‘extremely important’ (SM Table 20, column 2). For 2019, we then weighted those responses (SM Table 20, column 4) by the proportion of the Australian population made up by each age-bracket (SM Table 20, column 3). We then summed these weighted proportions to calculate an indicator of aggregate public opinion relating to our Q1 (SM Table 20, lowest row).

Then, we took the ABS population projections by age through to 2066, and explored how this measure of aggregate public opinion may change over time. We explored two hypothetical scenarios:

Scenario 1: Future young generations are equally as concerned as current young generations

This scenario ‘attaches’ the % of the age bracket answering ‘extremely important’ to Q1 to the age bracket as a fixed measure which moves with that age bracket over time. As we add new, young people to the voting population, we predict their opinion score as being equivalent to that of young people in our 2019 data. The key question explored in this scenario is therefore ‘If everyone’s views in 2019 remain as they are now into the future, and future young people hold views equal to 2019’s young people, how will aggregate opinion on climate change likely change into the future?’.

Scenario 2: Future young generations are more concerned than current young generations

This scenario adopts the same approach as scenario 1, except in this scenario we project future young people will hold opinion in line with *or stronger than* current levels responding ‘extremely important’ to our Q1. Therefore, we once again ‘attach’ opinion rates as they are in 2019 to each age bracket as they age, but as young people enter the voting population, we predict their views on climate change to be more pronounced than the young people represented in our 2019 data. As a result, this scenario required an additional analytical step.

Using the 2019 data (SM Table 20), we conducted linear regression on the % of each age bracket that answered ‘extremely important’ to Q1, predicted by our 5-year age brackets. Our linear model was: opinion = 73.5293 – 0.4829x, where x = the youngest age in the age bracket (i.e. 18 for 18-22) ($p < 0.001$, DF = 14, $R^2 = 0.5869$, SM Figure 22).

Our linear model therefore tells us that for each *1 year* increase in age, the percent of people in that age group who answer ‘extremely important’ to Q1 decreases by 0.4829. Equating this to the 5-year age brackets that we use in the scenario therefore tells us that for each *5 year* increase in age (i.e. each step increase in our age brackets), the percent answering Q1 with ‘extremely important’ decreases by **2.4145**. Or, put inversely, when the population is broken up into 5-year age brackets, the percent of each age bracket answering ‘extremely important’ to Q1 increases by 2.4% in comparison to the 5-year age bracket immediately older than them.

To return to the age projections scenario, we used this linear regression coefficient to predict potential future aggregate public opinion by *increasing* the percent of future young people answering Q1 with ‘extremely important’ by 2.4145 for each 5-year age bracket in each 5-year time period.

An important point to note is that our % answering ‘extremely important’ to Q1 for the age group 93-97 (our oldest age group) is zero. There were 9 respondents in this age group, so it is not a matter of missing data. One option we considered was to exclude this age group from our regression analysis that yielded us the **2.4145**% 5-yearly increase. However, we decided against this as we did not want to begin excluding data from our analysis when there was no ‘problem’ with the nature of that data.

Perhaps the oldest respondents to the survey simply hold opinion that is non-linearly different from younger age groups.

Although we elected to maintain our age projection analysis including the zero response score for the oldest respondent group, in the interests of transparency we re-ran the linear regression with this age group excluded to show the effect on our coefficient. Linear model with the 93+ respondents excluded: 66.7702 – 0.3194x, where x = the youngest age in the age bracket (i.e. 18 for 18-22) ($p < 0.001$, DF = 13, $R^2 = 0.681$). The difference to the slope coefficient is that with the 93+ age group included, we had a slope of 0.4829. Excluding this age group gives a slope of 0.3194. This slope, 0.3194, over 5 years equals 1.597 (compared to 2.4145).

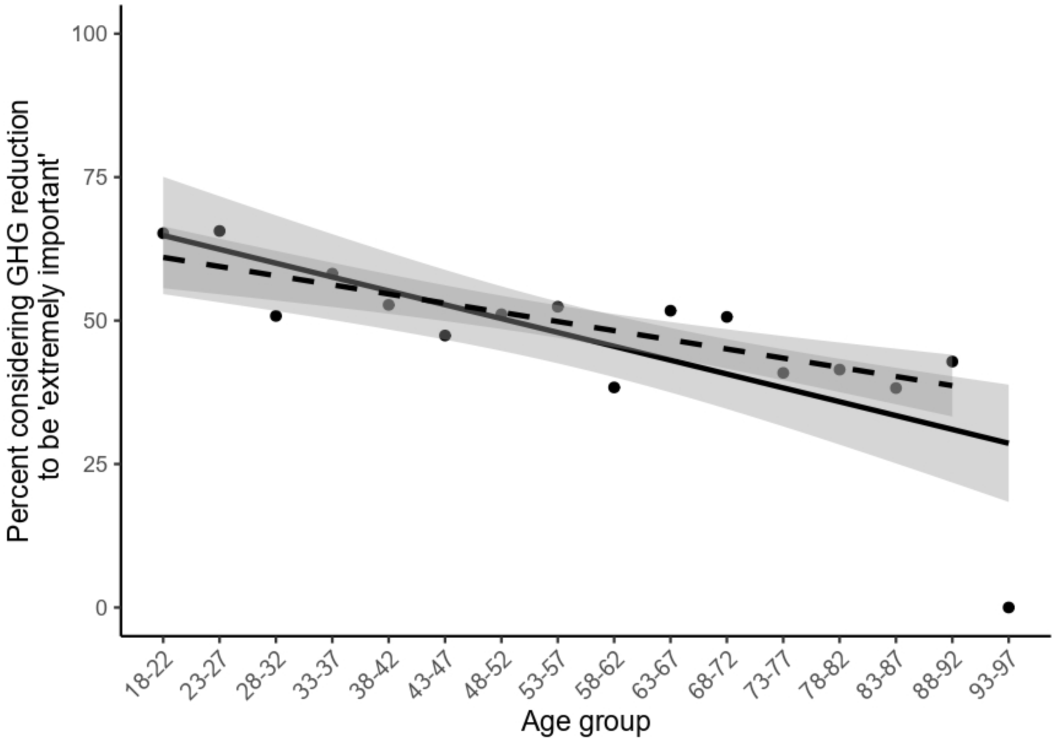

SM Figure 22: Scatterplot showing relationship between age (by 5 year age bracket) and proportion of that age bracket answering Q1 with 'extremely important'. Solid line indicates linear model including the 93+ age group; dashed line indicates linear model with the 93+ age group excluded. Shading represents standard error of the linear models.

Our age projections calculations are shown in SM Tables 20-22. Summary of findings of the age projection analysis is presented in SM Table 23 and SM Figure 23.

SM Table 20: Responses to Q1, by age bracket, with population weightings from ABS population projections (2019 data).

Age bracket	Age bracket as percent of Australian population	Percent answering Q1 with 'extremely important'	Percent answering Q1 with 'extremely important' by age-bracket weighted by relative percent of Australian population
**18-22**	8.3	65.2	5.4
**23-27**	9.5	65.6	6.3
**28-32**	9.7	50.8	4.9
**33-37**	9.4	58.2	5.5
**38-42**	8.3	52.7	4.4
**43-47**	8.3	47.4	3.9
**48-52**	8.1	51.1	4.1
**53-57**	7.8	52.4	4.1
**58-62**	7.4	38.4	2.8
**63-67**	6.5	51.7	3.4
**68-72**	5.9	50.6	3.0
**73-77**	4.3	40.9	1.7
**78-82**	3.0	41.5	1.2
**83-87**	1.9	38.2	0.7
**88-92**	1.1	42.9	0.5
**93-97**	0.4	0.0	0.0
**98+**	0.1	NA	NA
**TOTAL**	**100**	NA	**52.0**

SM Table 21a: Scenario 1 **-** Age projection calculations based on ABS population projections and responses to Q1. Column labels abbreviated to fit data: **% Pop** = Age bracket as percent of Australian population. **% Q1 EI** = Percent answering Q1 with 'extremely important'. **% Q1 W** = Percent answering Q1 with 'extremely important' by age-bracket weighted by relative percent of Australian population.

**Age bracket**	**2024**			**2029**			**2034**			**2039**			**2044**		
	**% Pop**	**% Q1 EI**	**% Q1 W**	**% Pop**	**% Q1 EI**	**% Q1 W**	**% Pop**	**% Q1 EI**	**% Q1 W**	**% Pop**	**% Q1 EI**	**% Q1 W**	**% Pop**	**% Q1 EI**	**% Q1 W**
**18-22**	8.05	65.42	5.27	8.26	65.42	5.40	8.03	65.42	5.25	7.85	65.42	5.13	7.92	65.41	5.18
**23-27**	9.34	65.22	6.09	8.92	65.42	5.84	9.05	65.42	5.92	8.79	65.42	5.75	8.55	65.37	5.59
**28-32**	9.75	65.62	6.40	9.48	65.22	6.18	9.09	65.42	5.95	9.20	65.42	6.02	8.93	65.42	5.84
**33-37**	9.50	50.81	4.83	9.50	65.62	6.24	9.28	65.22	6.05	8.94	65.42	5.85	9.04	65.42	5.91
**38-42**	8.98	58.18	5.22	9.06	50.81	4.60	9.10	65.62	5.97	8.93	65.22	5.82	8.61	65.42	5.63
**43-47**	7.81	52.74	4.12	8.42	58.18	4.90	8.54	50.81	4.34	8.61	65.62	5.65	8.47	65.22	5.52
**48-52**	7.66	47.40	3.63	7.22	52.74	3.81	7.82	58.18	4.55	7.98	50.81	4.05	8.07	65.62	5.29
**53-57**	7.39	51.10	3.78	7.00	47.40	3.32	6.65	52.74	3.51	7.24	58.18	4.21	7.41	50.81	3.76
**58-62**	7.05	52.41	3.69	6.72	51.10	3.43	6.41	47.40	3.04	6.13	52.74	3.23	6.70	58.18	3.90
**63-67**	6.65	38.35	2.55	6.36	52.41	3.33	6.12	51.10	3.13	5.88	47.40	2.79	5.65	52.74	2.98
**68-72**	5.72	51.72	2.96	5.89	38.35	2.26	5.70	52.41	2.99	5.53	51.10	2.83	5.34	47.40	2.53
**73-77**	4.98	50.64	2.52	4.92	51.72	2.54	5.13	38.35	1.97	5.02	52.41	2.63	4.90	51.10	2.51
**78-82**	3.41	40.88	1.40	4.03	50.64	2.04	4.06	51.72	2.10	4.30	38.35	1.65	4.24	52.41	2.22
**83-87**	2.11	41.46	0.87	2.46	40.88	1.00	2.96	50.64	1.50	3.04	51.72	1.57	3.27	38.35	1.25
**88-92**	1.06	38.24	0.40	1.20	41.46	0.50	1.44	40.88	0.59	1.77	50.64	0.90	1.88	51.72	0.97
**93-97**	0.40	42.86	0.17	0.40	38.24	0.15	0.47	41.46	0.19	0.59	40.88	0.24	0.77	50.64	0.39
**98+**	0.14	0.00	0.00	0.15	42.86	0.07	0.16	38.24	0.06	0.19	41.46	0.08	0.26	40.88	0.11
**TOTAL**	**100.00**	NA	**53.91**	**100.00**	NA	**55.62**	**100.00**	NA	**57.10**	**100.00**	NA	**58.41**	**100.00**	NA	**59.59**

Table continues next page.

SM Table 21b: Scenario 1 - Age projection calculations based on ABS population projections and responses to Q1. Column labels abbreviated to fit data: **% Pop** = Age bracket as percent of Australian population. **% Q1 EI** = Percent answering Q1 with ‘extremely important’. **% Q1 W** = Percent answering Q1 with ‘extremely important’ by age-bracket weighted by relative percent of Australian population.

Age bracket	2049			2054			2059			2064		
	% Pop	% Q1 EI	% Q1 W	% Pop	% Q1 EI	% Q1 W	% Pop	% Q1 EI	% Q1 W	% Pop	% Q1 EI	% Q1 W
**18-22**	7.84	65.41	5.13	7.70	65.41	5.04	7.58	65.41	4.96	7.47	65.41	4.88
**23-27**	8.57	65.41	5.61	8.45	65.41	5.53	8.28	65.41	5.41	8.12	65.41	5.31
**28-32**	8.69	65.37	5.68	8.69	65.41	5.69	8.56	65.41	5.60	8.38	65.41	5.48
**33-37**	8.78	65.42	5.75	8.56	65.37	5.59	8.55	65.41	5.60	8.43	65.41	5.51
**38-42**	8.71	65.42	5.70	8.49	65.42	5.55	8.28	65.37	5.41	8.28	65.41	5.42
**43-47**	8.19	65.42	5.36	8.30	65.42	5.43	8.10	65.42	5.30	7.92	65.37	5.18
**48-52**	7.95	65.22	5.19	7.72	65.42	5.05	7.84	65.42	5.13	7.67	65.42	5.01
**53-57**	7.52	65.62	4.93	7.44	65.22	4.85	7.24	65.42	4.73	7.37	65.42	4.82
**58-62**	6.88	50.81	3.49	7.00	65.62	4.59	6.94	65.22	4.53	6.77	65.42	4.43
**63-67**	6.19	58.18	3.60	6.38	50.81	3.24	6.51	65.62	4.27	6.48	65.22	4.22
**68-72**	5.15	52.74	2.72	5.67	58.18	3.30	5.87	50.81	2.98	6.01	65.62	3.94
**73-77**	4.76	47.40	2.26	4.62	52.74	2.44	5.11	58.18	2.98	5.31	50.81	2.70
**78-82**	4.18	51.10	2.14	4.09	47.40	1.94	4.01	52.74	2.11	4.46	58.18	2.60
**83-87**	3.27	52.41	1.71	3.28	51.10	1.68	3.24	47.40	1.54	3.22	52.74	1.70
**88-92**	2.08	38.35	0.80	2.13	52.41	1.11	2.20	51.10	1.12	2.21	47.40	1.05
**93-97**	0.86	51.72	0.45	1.00	38.35	0.38	1.07	52.41	0.56	1.16	51.10	0.59
**98+**	0.38	50.64	0.19	0.48	51.72	0.25	0.62	38.35	0.24	0.75	52.41	0.39
**TOTAL**	**100.00**	NA	**60.69**	**100.00**	NA	**61.66**	**100.00**	NA	**62.47**	**100.00**	NA	**63.24**

SM Table 22a: Scenario 2 - Age projection calculations based on ABS population projections and responses to Q1. Column labels abbreviated to fit data: **% Pop** = Age bracket as percent of Australian population. **% Q1 EI** = Percent answering Q1 with 'extremely important'. **% Q1 Wa** = Percent answering Q1 with 'extremely important' by age-bracket weighted by relative percent of Australian population (93+ age group included). **% Q1 Wb** = Percent answering Q1 with 'extremely important' by age-bracket weighted by relative percent of Australian population (93+ age group excluded).

Age bracket	2024				2029				2034				2039			
	% Pop	% Q1 EI	% Q1 Wa	% Q1 Wb	% Pop	% Q1 EI	% Q1 Wa	% Q1 Wb	% Pop	% Q1 EI	% Q1 Wa	% Q1 Wb	% Pop	% Q1 EI	% Q1 Wa	% Q1 Wb
**18-22**	8.05	67.63	5.45	5.38	8.26	70.05	5.78	5.65	8.03	72.46	5.82	5.62	7.85	74.88	5.87	5.62
**23-27**	9.34	65.22	6.09	6.09	8.92	67.63	6.04	5.96	9.05	70.05	6.34	6.19	8.79	72.46	6.37	6.15
**28-32**	9.75	65.62	6.40	6.40	9.48	65.22	6.18	6.18	9.09	67.63	6.15	6.07	9.20	70.05	6.45	6.30
**33-37**	9.50	50.81	4.83	4.83	9.50	65.62	6.24	6.24	9.28	65.22	6.05	6.05	8.94	67.63	6.05	5.97
**38-42**	8.98	58.18	5.22	5.22	9.06	50.81	4.60	4.60	9.10	65.62	5.97	5.97	8.93	65.22	5.82	5.82
**43-47**	7.81	52.74	4.12	4.12	8.42	58.18	4.90	4.90	8.54	50.81	4.34	4.34	8.61	65.62	5.65	5.65
**48-52**	7.66	47.40	3.63	3.63	7.22	52.74	3.81	3.81	7.82	58.18	4.55	4.55	7.98	50.81	4.05	4.05
**53-57**	7.39	51.10	3.78	3.78	7.00	47.40	3.32	3.32	6.65	52.74	3.51	3.51	7.24	58.18	4.21	4.21
**58-62**	7.05	52.41	3.69	3.69	6.72	51.10	3.43	3.43	6.41	47.40	3.04	3.04	6.13	52.74	3.23	3.23
**63-67**	6.65	38.35	2.55	2.55	6.36	52.41	3.33	3.33	6.12	51.10	3.13	3.13	5.88	47.40	2.79	2.79
**68-72**	5.72	51.72	2.96	2.96	5.89	38.35	2.26	2.26	5.70	52.41	2.99	2.99	5.53	51.10	2.83	2.83
**73-77**	4.98	50.64	2.52	2.52	4.92	51.72	2.54	2.54	5.13	38.35	1.97	1.97	5.02	52.41	2.63	2.63
**78-82**	3.41	40.88	1.40	1.40	4.03	50.64	2.04	2.04	4.06	51.72	2.10	2.10	4.30	38.35	1.65	1.65
**83-87**	2.11	41.46	0.87	0.87	2.46	40.88	1.00	1.00	2.96	50.64	1.50	1.50	3.04	51.72	1.57	1.57
**88-92**	1.06	38.24	0.40	0.40	1.20	41.46	0.50	0.50	1.44	40.88	0.59	0.59	1.77	50.64	0.90	0.90
**93-97**	0.40	42.86	0.17	0.17	0.40	38.24	0.15	0.15	0.47	41.46	0.19	0.19	0.59	40.88	0.24	0.24
**98+**	0.14	0.00	0.00	0.00	0.15	42.86	0.07	0.07	0.16	38.24	0.06	0.06	0.19	41.46	0.08	0.08
**TOTAL**	**100.00**	NA	**54.09**	**54.02**	**100.00**	NA	**56.20**	**55.99**	**100.00**	NA	**58.28**	**57.87**	**100.00**	NA	**60.40**	**59.70**

Table continues next page.

SM Table 22b: Scenario 2 - Age projection calculations based on ABS population projections and responses to Q1. Column labels abbreviated to fit data: **% Pop** = Age bracket as percent of Australian population. **% Q1 EI** = Percent answering Q1 with ‘extremely important’. **% Q1 Wa** = Percent answering Q1 with ‘extremely important’ by age-bracket weighted by relative percent of Australian population (93+ age group included). **% Q1 Wb** = Percent answering Q1 with ‘extremely important’ by age-bracket weighted by relative percent of Australian population (93+ age group excluded).

Age bracket	2044				2049				2054			
	% Pop	% Q1 EI	% Q1 Wa	% Q1 Wb	% Pop	% Q1 EI	% Q1 Wa	% Q1 Wb	% Pop	% Q1 EI	% Q1 Wa	% Q1 Wb
**18-22**	7.92	77.29	6.12	5.80	7.84	79.71	6.25	5.86	7.70	82.12	6.32	5.88
**23-27**	8.55	74.88	6.41	6.13	8.57	77.29	6.62	6.27	8.45	79.71	6.74	6.32
**28-32**	8.93	72.46	6.47	6.25	8.69	74.88	6.51	6.23	8.69	77.29	6.72	6.36
**33-37**	9.04	70.05	6.33	6.18	8.78	72.46	6.36	6.15	8.56	74.88	6.41	6.13
**38-42**	8.61	67.63	5.82	5.75	8.71	70.05	6.10	5.96	8.49	72.46	6.15	5.94
**43-47**	8.47	65.22	5.52	5.52	8.19	67.63	5.54	5.47	8.30	70.05	5.81	5.68
**48-52**	8.07	65.62	5.29	5.29	7.95	65.22	5.19	5.19	7.72	67.63	5.22	5.16
**53-57**	7.41	50.81	3.76	3.76	7.52	65.62	4.93	4.93	7.44	65.22	4.85	4.85
**58-62**	6.70	58.18	3.90	3.90	6.88	50.81	3.49	3.49	7.00	65.62	4.59	4.59
**63-67**	5.65	52.74	2.98	2.98	6.19	58.18	3.60	3.60	6.38	50.81	3.24	3.24
**68-72**	5.34	47.40	2.53	2.53	5.15	52.74	2.72	2.72	5.67	58.18	3.30	3.30
**73-77**	4.90	51.10	2.51	2.51	4.76	47.40	2.26	2.26	4.62	52.74	2.44	2.44
**78-82**	4.24	52.41	2.22	2.22	4.18	51.10	2.14	2.14	4.09	47.40	1.94	1.94
**83-87**	3.27	38.35	1.25	1.25	3.27	52.41	1.71	1.71	3.28	51.10	1.68	1.68
**88-92**	1.88	51.72	0.97	0.97	2.08	38.35	0.80	0.80	2.13	52.41	1.11	1.11
**93-97**	0.77	50.64	0.39	0.39	0.86	51.72	0.45	0.45	1.00	38.35	0.38	0.38
**98+**	0.26	40.88	0.11	0.11	0.38	50.64	0.19	0.19	0.48	51.72	0.25	0.25
**TOTAL**	**100.00**	NA	**62.59**	**61.55**	**100.00**	NA	**64.86**	**63.41**	**100.00**	NA	**67.16**	**65.26**

Table continues next page.

SM Table 22c: Scenario 2 - Age projection calculations based on ABS population projections and responses to Q1. Column labels abbreviated to fit data: **% Pop** = Age bracket as percent of Australian population. **% Q1 EI** = Percent answering Q1 with 'extremely important'. **% Q1 Wa** = Percent answering Q1 with 'extremely important' by age-bracket weighted by relative percent of Australian population (93+ age group included). **% Q1 Wb** = Percent answering Q1 with 'extremely important' by age-bracket weighted by relative percent of Australian population (93+ age group excluded).

**2059**				**2064**			
**% Pop**	**% Q1 EI**	**% Q1 Wa**	**% Q1 Wb**	**% Pop**	**% Q1 EI**	**% Q1 Wa**	**% Q1 Wb**
7.58	84.54	6.41	5.91	7.47	86.95	6.49	5.94
8.28	82.12	6.80	6.32	8.12	84.54	6.86	6.33
8.56	79.71	6.83	6.41	8.38	82.12	6.88	6.40
8.55	77.29	6.61	6.26	8.43	79.71	6.72	6.30
8.28	74.88	6.20	5.93	8.28	77.29	6.40	6.06
8.10	72.46	5.87	5.67	7.92	74.88	5.93	5.67
7.84	70.05	5.49	5.36	7.67	72.46	5.55	5.37
7.24	67.63	4.89	4.83	7.37	70.05	5.16	5.04
6.94	65.22	4.53	4.53	6.77	67.63	4.58	4.53
6.51	65.62	4.27	4.27	6.48	65.22	4.22	4.22
5.87	50.81	2.98	2.98	6.01	65.62	3.94	3.94
5.11	58.18	2.98	2.98	5.31	50.81	2.70	2.70
4.01	52.74	2.11	2.11	4.46	58.18	2.60	2.60
3.24	47.40	1.54	1.54	3.22	52.74	1.70	1.70
2.20	51.10	1.12	1.12	2.21	47.40	1.05	1.05
1.07	52.41	0.56	0.56	1.16	51.10	0.59	0.59
0.62	38.35	0.24	0.24	0.75	52.41	0.39	0.39
**100.00**	NA	**69.43**	**67.03**	**100.00**	NA	**71.78**	**68.84**

SM Table 23: Summary of age projection scenarios and impact on aggregate public opinion associated with percent of population answering ‘extremely important’ to Q1. Scenario B results are displayed according to use of a slope for predicting future aggregate public opinion that either includes or excludes the 93+ age group.

Year	Percent of Australian population of voting age answering ‘extremely important’ to Q1		
	Scenario A	Scenario B (93+ included)	Scenario B (93+ excluded)
**2019**	52.0	52.0	52.0
**2024**	53.9	54.1	54.0
**2029**	55.6	56.2	56.2
**2034**	57.1	58.3	57.9
**2039**	58.4	60.4	59.7
**2044**	59.6	62.6	61.6
**2049**	60.7	64.9	63.4
**2054**	61.7	67.2	65.3
**2059**	62.5	69.4	67.0
**2064**	63.2	71.8	68.9

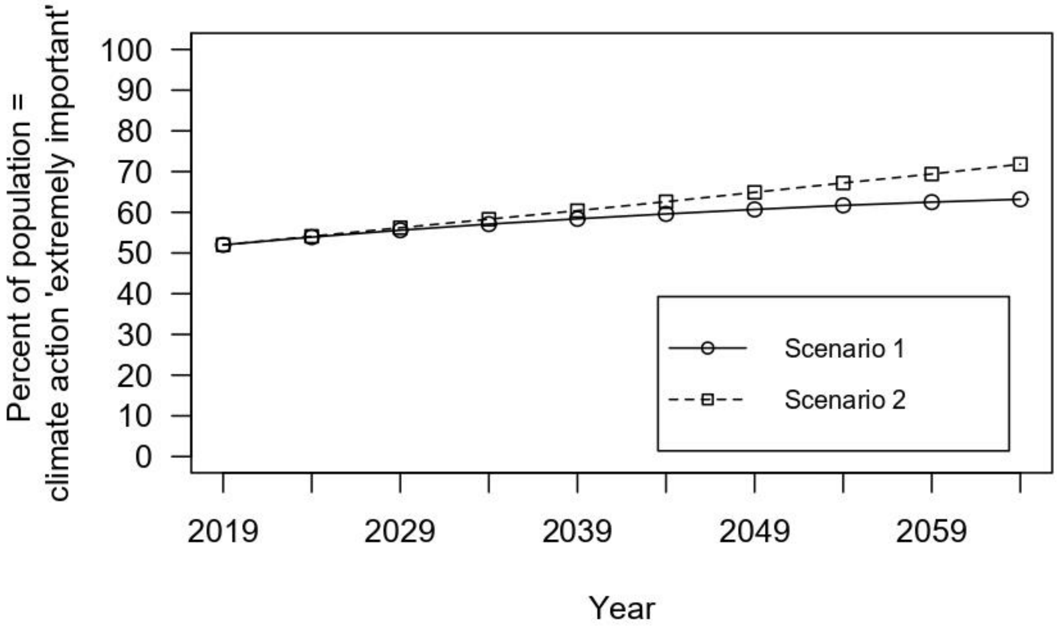

SM Figure 23: Line graph showing two scenarios of projected aggregate public opinion represented as percent of Australians of voting age holding opinion in line with answering ‘extremely important’ to Q1. Scenario 2 includes the 93+ age group (scenario 2 projections excluding the 93+ age group would fall between the two lines plotted in the figure).

Cross tabulations: key questions

We include here a series of cross-tabulations for our 4 climate questions (SM Figures 24-29).

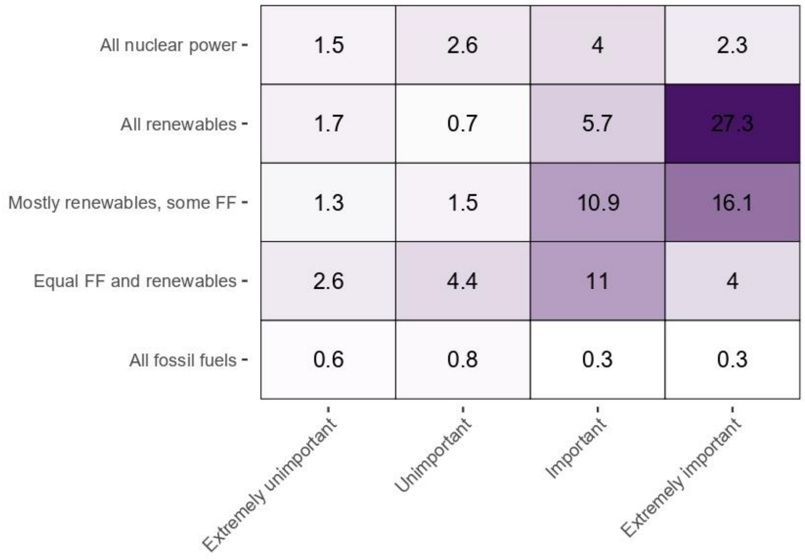

SM Figure 24: Cross tabulation of Q1 (x axis) and Q2 (y axis). (Q1. In your view, how important or unimportant is it that Australia takes action to reduce greenhouse gas emissions in order to help limit future climate change? Q2. In your view, which energy source or mix of energy sources should provide Australia's electricity in 2050?). Figures show percent of all responses.

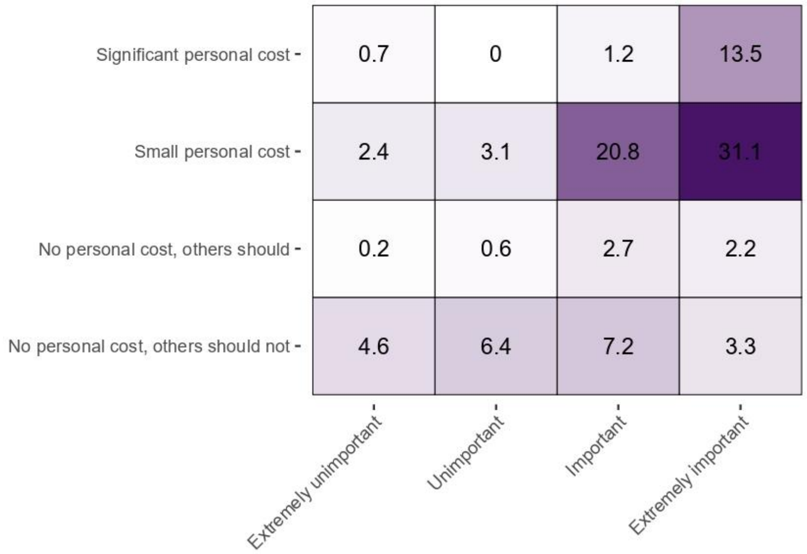

SM Figure 25: Cross tabulation of Q1 (x axis) and Q3 (y axis). (Q1. In your view, how important or unimportant is it that Australia takes action to reduce greenhouse gas emissions in order to help limit future climate change? Q3. To what extent are you prepared to accept a personal cost in order to support action to reduce Australia's emissions?). Figures show percent of all responses.

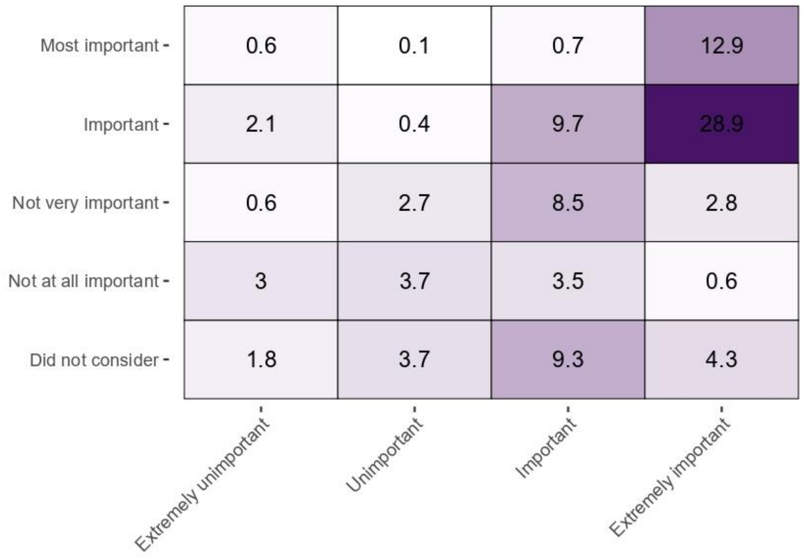

SM Figure 26: Cross tabulation of Q1 (x axis) and Q4 (y axis). (Q1. In your view, how important or unimportant is it that Australia takes action to reduce greenhouse gas emissions in order to help limit future climate change? Q4. How much did the issue of climate change influence your vote in the 2019 Federal election? For you personally, would you say climate change was...?). Figures show percent of all responses.

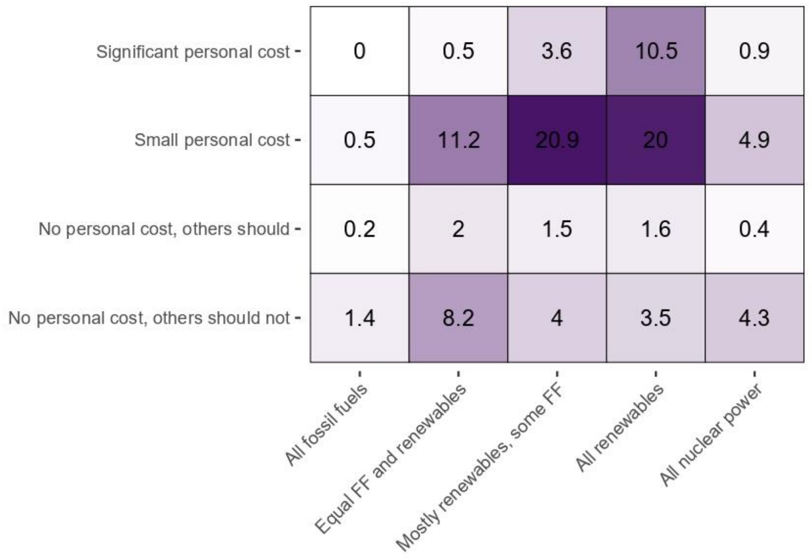

SM Figure 27: Cross tabulation of Q2 (x axis) and Q3 (y axis). (Q2. In your view, which energy source or mix of energy sources should provide Australia's electricity in 2050? Q3. To what extent are you prepared to accept a personal cost in order to support action to reduce Australia's emissions?). Figures show percent of all responses.

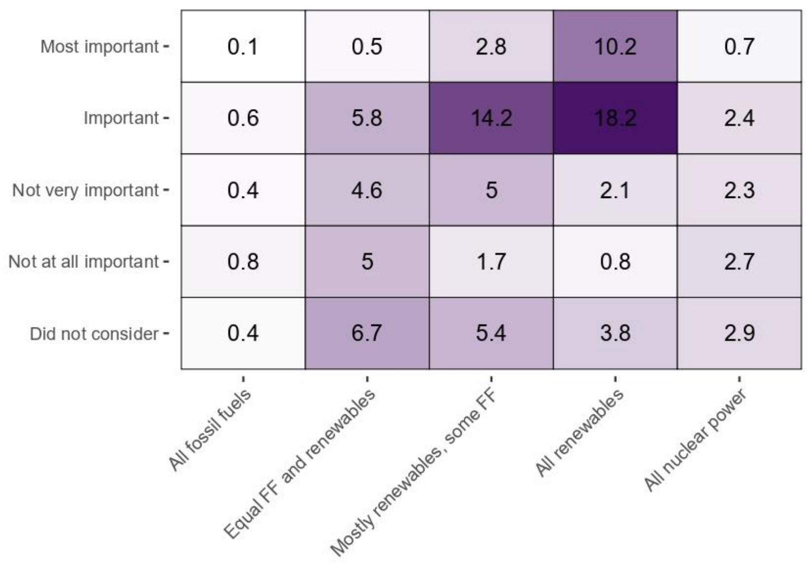

SM Figure 28: Cross tabulation of Q2 (x axis) and Q4 (y axis). (Q2. In your view, which energy source or mix of energy sources should provide Australia's electricity in 2050? Q4. How much did the issue of climate change influence your vote in the 2019 Federal election? For you personally, would you say climate change was...?). Figures show percent of all responses.

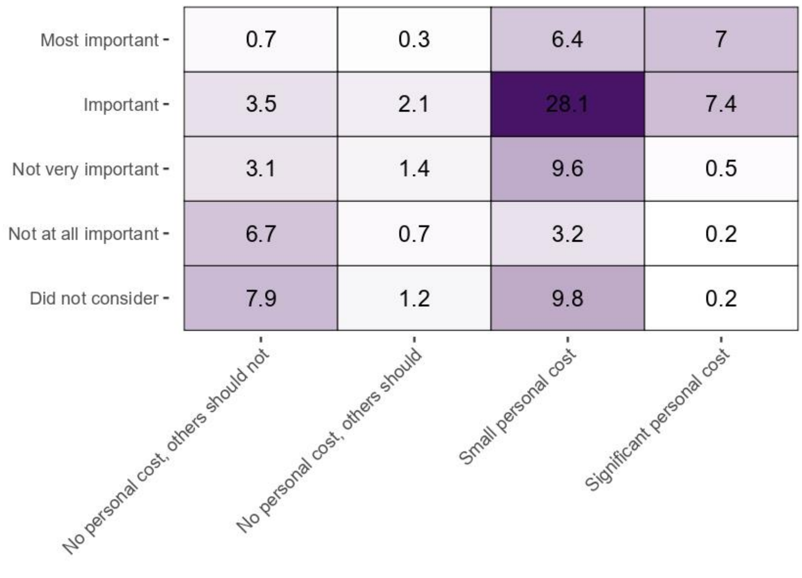

SM Figure 29: Cross tabulation of Q3 (x axis) and Q4 (y axis). (Q3. To what extent are you prepared to accept a personal cost in order to support action to reduce Australia's emissions? Q4. How much did the issue of climate change influence your vote in the 2019 Federal election? For you personally, would you say climate change was...?). Figures show percent of all responses.

References

Australian Bureau of Statistics. 2018a. *3101.0 - Australian Demographic Statistics, Sep 2018* [Online]. Australian Government. Available: https://www.abs.gov.au/AUSSTATS/abs@.nsf/allprimarymainfeatures/3ABD776A23EB5531CA25841E001299D5 [Accessed 11 November 2019].

Australian Bureau of Statistics. 2018b. *Population Projections, Australia, 2017 (base) - 2066, ABS Catalogue No. 3220, November 2018*. Australian Government. Available: http://stat.data.abs.gov.au/Index.aspx?DatasetCode=POP_PROJ_2011.

Liddell, T. M. & Kruschke, J. K. 2018. Analyzing ordinal data with metric models: What could possibly go wrong? *Journal of Experimental Social Psychology,* 79**,** 328-348.

Park, C. S., Chung, W. B., Choi, Y. S., Kim, P. J., Lee, J. M., Baek, K.-H., Kim, H. Y., Yoo, K. D., Song, K.-H., Chung, W. S., Seung, K. B., Lee, M. Y. & Kwon, H.-S. 2015. Acute Myocardial Infarction Is a Risk Factor for New Onset Diabetes in Patients with Coronary Artery Disease. *PLOS ONE,* 10**,** e0136354.

R Core Team 2013. *R: A language and environment for statistical computing*. Vienna, Austria, URL http://www.R-project.org/, R Foundation for Statistical Computing.

Tranter, B. 2011. Political divisions over climate change and environmental issues in Australia. *Environmental Politics,* 20**,** 78-96.

Tranter, B. 2013. The Great Divide: Political Candidate and Voter Polarisation over Global Warming in Australia. *Australian Journal of Politics & History,* 59**,** 397-413.

Tranter, B. 2014. Social and political influences on environmentalism in Australia. *Journal of Sociology,* 50**,** 331-348.

Tranter, B. 2017. It's only natural: conservatives and climate change in Australia. *Environmental Sociology***,** 1-12.

Tranter, B. 2019. Does Public Knowledge of Climate Change Really Matter in Australia? *Environmental Communication***,** 1-18.

Tranter, B. & Booth, K. 2015. Scepticism in a changing climate: A cross-national study. *Global Environmental Change,* 33**,** 154-164.

Tranter, B. & Foxwell-Norton, K. 2020. Only in Queensland? Coal mines and voting in the 2019 Australian federal election. *Environmental Sociology***,** 1-12.

Tranter, B., Skrbiš, Z. & Smith, J. F. 2020. Poles Apart: Political Divisions over Climate Change Among Younger Australians. *Journal of Applied Youth Studies,* 3**,** 255-273.
